# Supplementary material for: hnRNPK Recruits PCGF3/5-PRC1 to the Xist RNA B-Repeat to Establish Polycomb-Mediated Chromosomal Silencing
Source: Mol Cell. 2017 Dec 7;68(5):955–969.e10. doi: 10.1016/j.molcel.2017.11.013 (PMC5735038; doi:10.1016/j.molcel.2017.11.013)
Supplement: Document S1. Figures S1–S7, Table S3, and Table S4 [file mmc1.pdf]

Molecular Cell, Volume *68*

Supplemental Information

hnRNPK Recruits PCGF3/5-PRC1 to the Xist RNA B-Repeat to Establish Polycomb-Mediated Chromosomal Silencing

Greta Pintacuda, Guifeng Wei, Chloë Roustan, Burcu Anil Kirmizitas, Nicolae Solcan, Andrea Cerase, Alfredo Castello, Shabaz Mohammed, Benoît Moindrot, Tatyana B. Nesterova, and Neil Brockdorff

Supplemental Figures

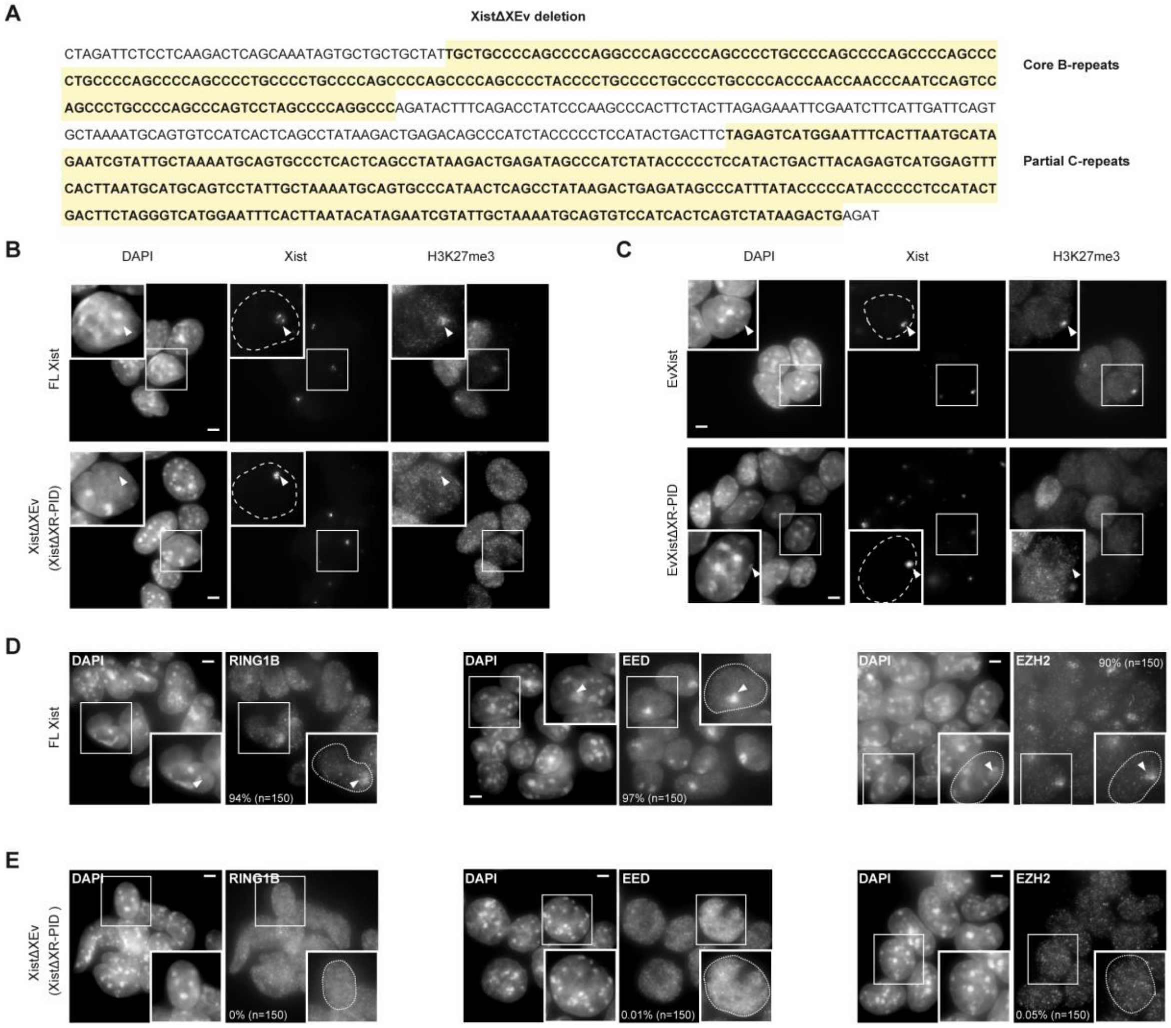

Figure S1. Related to Figure 1. Definition of Xist RNA elements required for Polycomb recruitment.

(A) The Xist sequence deleted in the XistΔXR-PID transgene is shown. The B-repeat and the partial C-repeat sequences are highlighted in yellow.

(B and C) Examples illustrating ImmunoFISH detection of Xist RNA and H3K27me3 after inducing cells with doxycycline for 24 h using the Xist transgene constructs indicated. Wide-field images represent average of 6 consecutive z-sections, cells in boxes were enlarged and represented as single z-sections. Arrows indicate Xist domains. DNA was counterstained with DAPI. Dotted lines in insets indicate outline of DAPI stained nuclei. Scale bar is 5 μm.

(D and E) Examples illustrating immunofluorescence detection of RING1B, EED, and EZH2 after inducing cells with doxycycline for 24 h using the Xist transgene constructs indicated. Scoring was for the number of cells with single nuclear domains. Wide-field images represent the average of 6 consecutive z-sections, cells in boxes were enlarged and represented as single z-sections. Arrows indicate domains of enrichment. DNA was counterstained with DAPI. Dotted lines in insets indicate outline of DAPI stained nuclei. Scale bar is 5 μm.

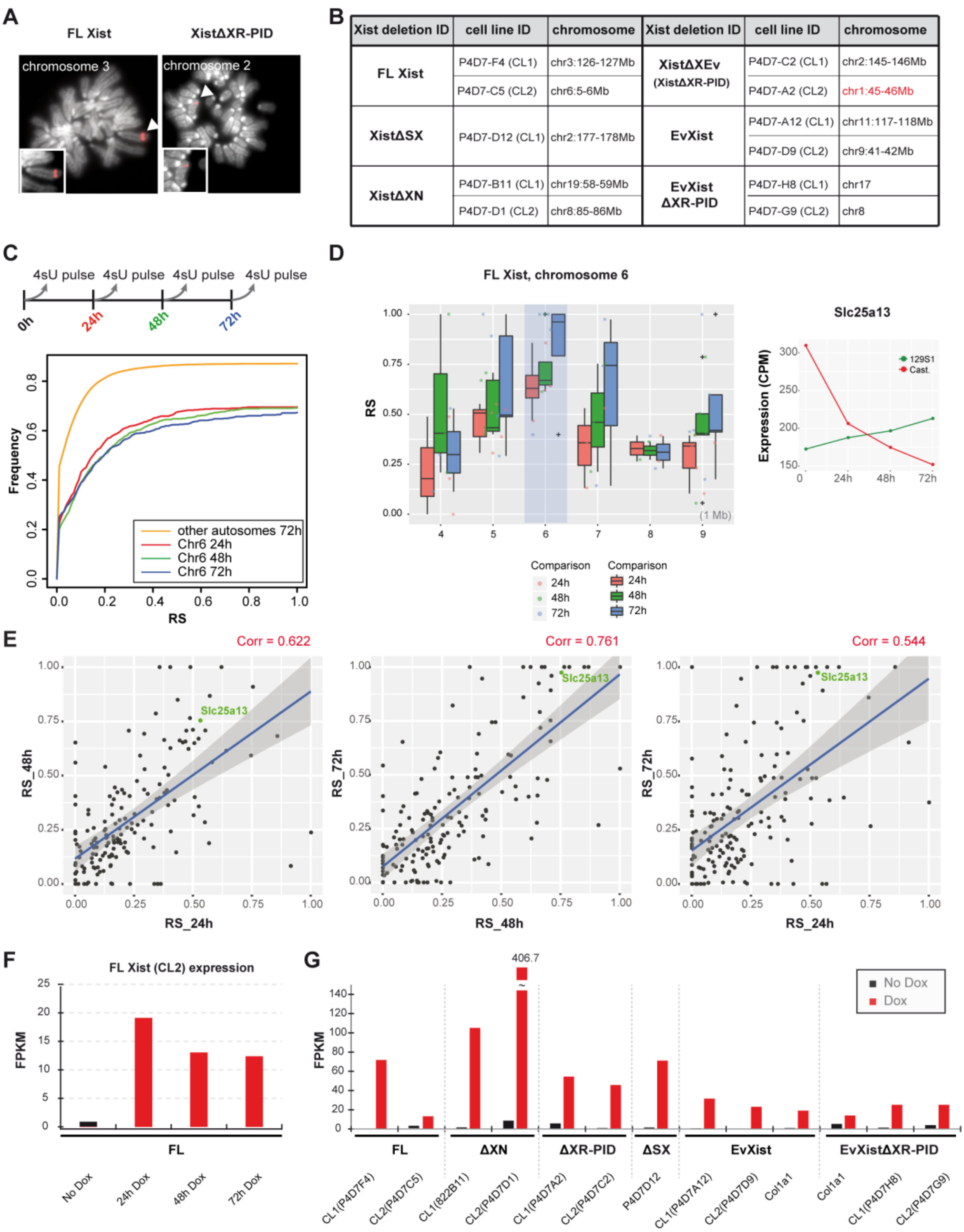

Xist deletion ID	cell line ID	chromosome	Xist deletion ID	cell line ID	chromosome
FL Xist	P4D7-F4 (CL1)	chr3:126-127Mb	XistΔXEv (XistΔXR-PID)	P4D7-C2 (CL1)	chr2:145-146Mb
	P4D7-C5 (CL2)	chr6:5-6Mb		P4D7-A2 (CL2)	chr1:45-46Mb
XistΔSX	P4D7-D12 (CL1)	chr2:177-178Mb	EvXist	P4D7-A12 (CL1)	chr11:117-118Mb
				P4D7-D9 (CL2)	chr9:41-42Mb
XistΔXN	P4D7-B11 (CL1)	chr19:58-59Mb	EvXist ΔXR-PID	P4D7-H8 (CL1)	chr17
	P4D7-D1 (CL2)	chr8:85-86Mb		P4D7-G9 (CL2)	chr8

Figure S2. Related to Figure 2. Gene silencing analysis for Xist transgenes

(A) Examples of DNA-FISH analysis mapping Xist transgenes on metaphase spreads. Inset illustrates zoom in of chromosome with Xist transgene. Arrowhead indicates chromosome

with Xist transgene signal (red). DNA was counterstained with DAPI.

(B) Chromosome location of the mapped Xist transgenes in this study, as revealed by RS analysis and confirmed by DNA FISH. All Xist transgenes were integrated in the Cast allele, except for cell line P4D7-A2 where it is in the 129S1 allele (marked in red).

(C) Cumulative distribution function plot showing the RS distribution for genes on chr6 during a time-course experiment. The curves with red, green, blue colours indicate the RS score for 24 h, 48 h, 72 h, respectively. The orange line shows RS on all other autosomes at 72 h, attributable to stochastic effects.

(D) The boxplot (left) shows RS distributions for genes located in the proximal 10 Mb window of chr6, illustrating that within that window, the 6th Mb region shows the maximum silencing. The allelic expression for Slc25a13 gene through the time-course experiment is illustrated (right). The red and green lines are Cast (Inactive) and 129S1 (Active) allele, respectively.

(E) Scatterplot showing correlation of the RS for genes located in the proximal 20 Mb region of chromosome 6 during the time-course experiment. The 99% confidential interval is highlighted in grey. *Slc25a13* gene is located in the maximal silencing region. Pearson's correlation coefficient for each comparison is shown above.

(F) Xist expression levels during a time-course of doxycycline induction are represented as FPKM for each time point.

(G) Xist expression levels are represented as FPKM for each cell line listed in (B) before and after 72 h doxycycline treatment.

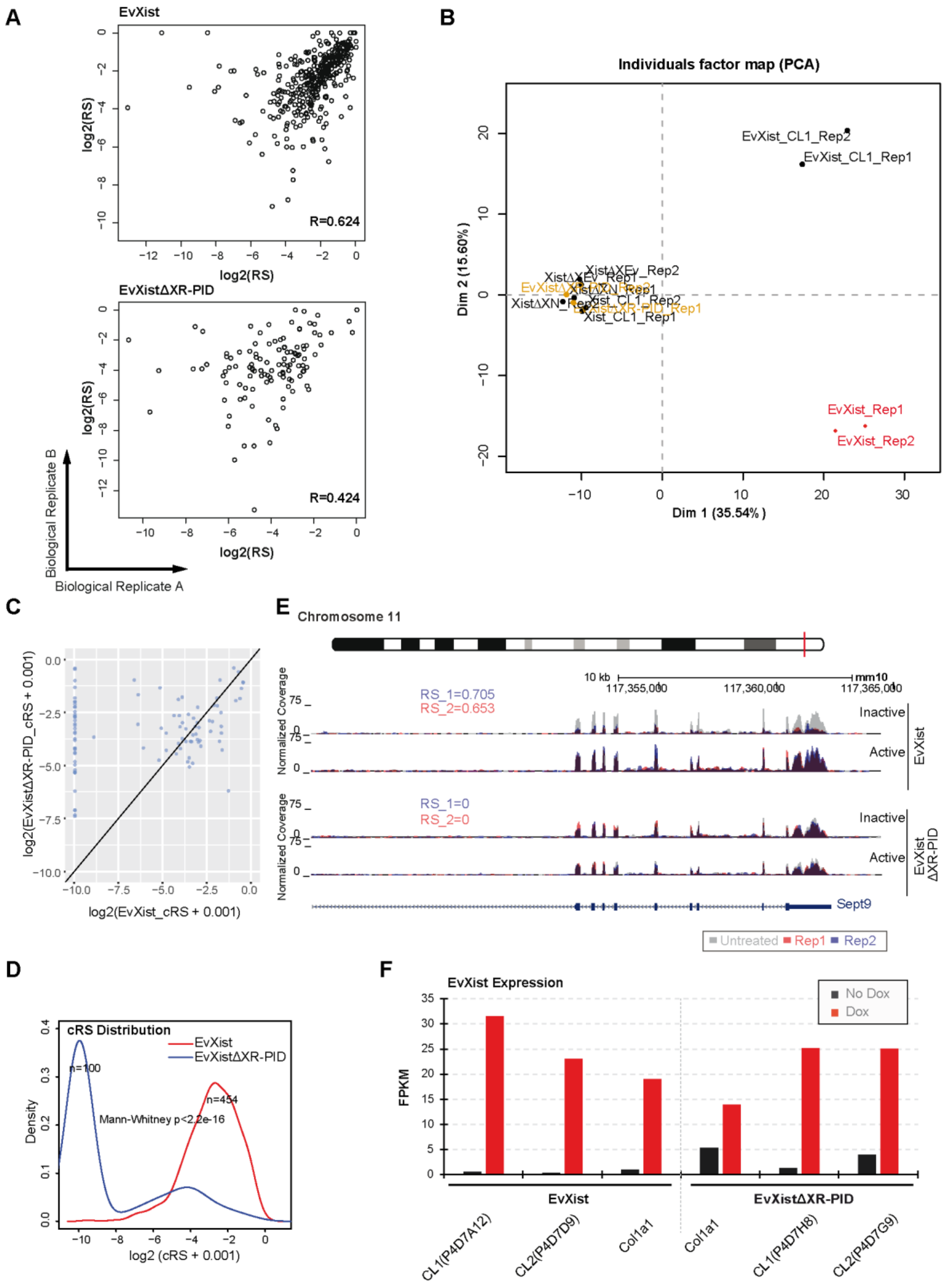
A
EvXist
R=0.624
log2(RS)
EvXistΔXR-PID
R=0.424
Biological Replicate A
Biological Replicate B
B
Individuals factor map (PCA)
Dim 1 (35.54%)
Dim 2 (15.60%)
EvXist_CL1_Rep2
EvXist_CL1_Rep1
EvXist_Rep1
EvXist_Rep2
C
log2(EvXist_cRS + 0.001)
log2(EvXistΔXR-PID_cRS + 0.001)
D
cRS Distribution
EvXist
EvXistΔXR-PID
n=100
n=454
Mann-Whitney p<2.2e-16
Density
log2 (cRS + 0.001)
E
Chromosome 11
RS_1=0.705
RS_2=0.653
RS_1=0
RS_2=0
Inactive
Active
Normalized Coverage
Sept9
Untreated
Rep1
Rep2
F
EvXist Expression
No Dox
Dox
FPKM
EvXist
EvXistΔXR-PID
CL1(P4D7A12)
CL2(P4D7D9)
Col1a1
CL1(P4D7H8)
CL2(P4D7G9)

Figure S3. Related to Figure 3. Chromosome 11 silencing by targeted EvXist and EvXistΔXR-PID transgenes

(A) RS correlation between two biological replicates. Upper: EvXist; Bottom: EvXistΔXR-PID. X and Y axis indicate log2-transformed RS.

(B) PCA Analysis of RS for distal 60 Mb genes on chromosome 11. EvXist and EvXistΔXR-PID cells are indicated by red and yellow dots respectively. Another independent cell line, with an insertion of EvXist (EvXist_CL1) in a different region of chromosome 11 is also shown for comparison.

(C) Scatterplot showing differences of the calibrated (c) RS between EvXist and EvXistΔXR-PID for the 100 genes which are silenced in EvXistΔXR-PID cells. Axis values indicate log2-transformed cRS.

(D) The overall calibrated RS density distribution for genes silenced by EvXist (red curve) or EvXistΔXR-PID (blue curve). The x-axis represents the log2-transformed cRS.

(E) The overlay genome browser tracks for the *Sept9* locus, indicated by red lines in the upper chromosome ideogram, is shown. RS and q-value for EvXist and EvXistΔXR-PID is indicated in the Figure. Q-values are 0 and 0.612 respectively. The grey tracks represent control condition without dox treatment, red and blue are two biological replicates of 72 h dox treatment. The active allele (129S1) and inactive allele (Cast) are indicated (right).

(F) Xist expression levels are represented as FPKM for each cell line before and after 72 h doxycycline treatment. For comparison, Xist expression level is represented for all cell lines harbouring EvXist or EvXistΔXR-PID, either randomly integrated in the genome (P4D7A12, P4D7D9, P4D7H8, P4D7G9), or targeted to the *Col1a1* locus.

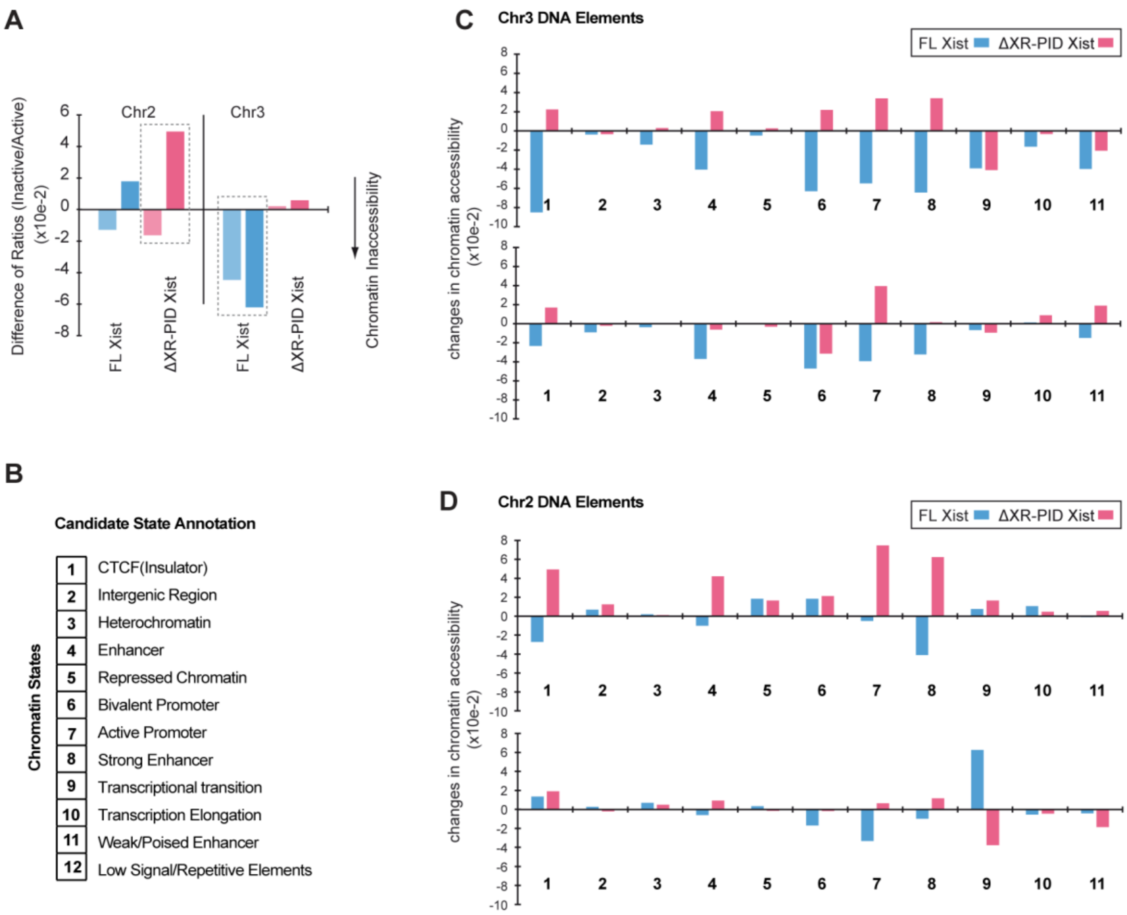

Figure S4. Related to Figure 4. XR-PID is specifically required for reduced chromatin accessibility of open chromatin regions over Xist domains

(A) Whole chromosome differences of allelic ratios (Inactive/Active) between Dox and NoDox cells expressing Xist (chr3) and XistΔXR-PID (chr2). Negative values indicate Xist-induced chromatin inaccessibility.

(B) 12 classes of ChromHMM states of mouse embryonic stem cells were employed to calculate the chromatin accessibility changes in each *cis*-element category upon Xist or XistΔXR-PID induction.

(C and D) Comparison of chromatin accessibility changes in DNA *cis*-elements on chr3 (C) and chr2 (D). The top and bottom part of each panel represents independent biological replicates. Blue and Red indicate FL Xist (integrated in chr3) and ΔXR-PID Xist expressing cells (integrated in chr2).

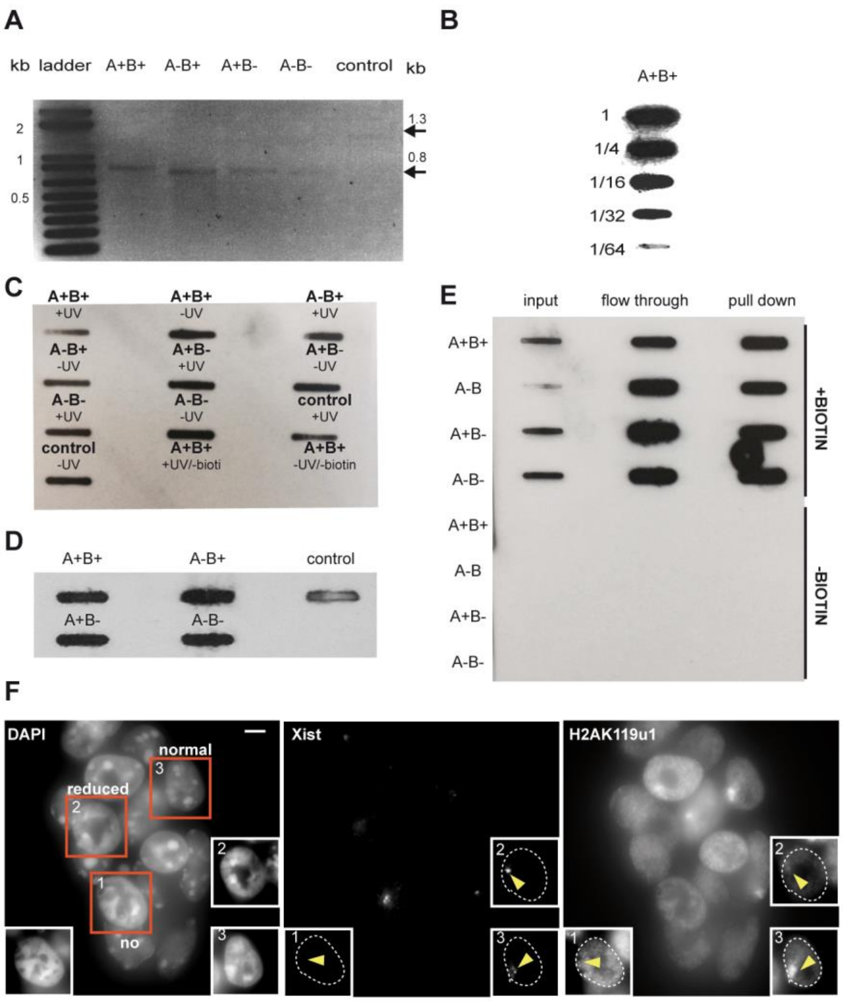

Figure S5. Related to Figure 5. Identification of hnRNPK as candidate B-repeat binding protein

(A) *In vitro* transcription products for each construct (labelled as in Fig.5A), analysed on denaturing gel (0.8% agarose). 1.3 kb FLuc RNA was used as a control.

(B) Titration of dUTP-biotin (starting concentration of 2.5 mM) to determine optimal incorporation for labelling of the same amount of *in vitro* transcribed Xist RNA, here shown for Xist A+B+. A 1/32 concentration was used for subsequent experiments.

(C) Slot blot assay of 1μg of RNA of each construct using biotin antibody. Biotin incorporation and detection was checked in the presence or absence of UV crosslinking. FLuc RNA was used as a control. Xist A+B+ without biotin was used as a negative control.

(D) Slot blot assay of constructs (named as in Fig.5A) using a biotin antibody for detection in the experimental conditions tested in B. 1 μg of RNA was loaded.

(E) Recovery of transcribed RNAs was verified by slot blot. 1 μg of the input, 1/10 of the flow-through, and 1/50 of the pull-down were loaded, determined by analysis of biotin levels. Non-biotinylated constructs were used as negative controls for the assay.

(F) Example of ImmunoFISH analysis of Xist RNA and H2AK119u1 after 24 h siRNA mediated knockdown of hnRNPK siRNA. Wide-field images represent average of 10 stacks. Insets show enlarged single stacks. Arrows point to Xist domains. Each box represents a different category of H2AK119ub1 domains as indicated, and quantified in Figure 5F. Scalebar is 5 μm.

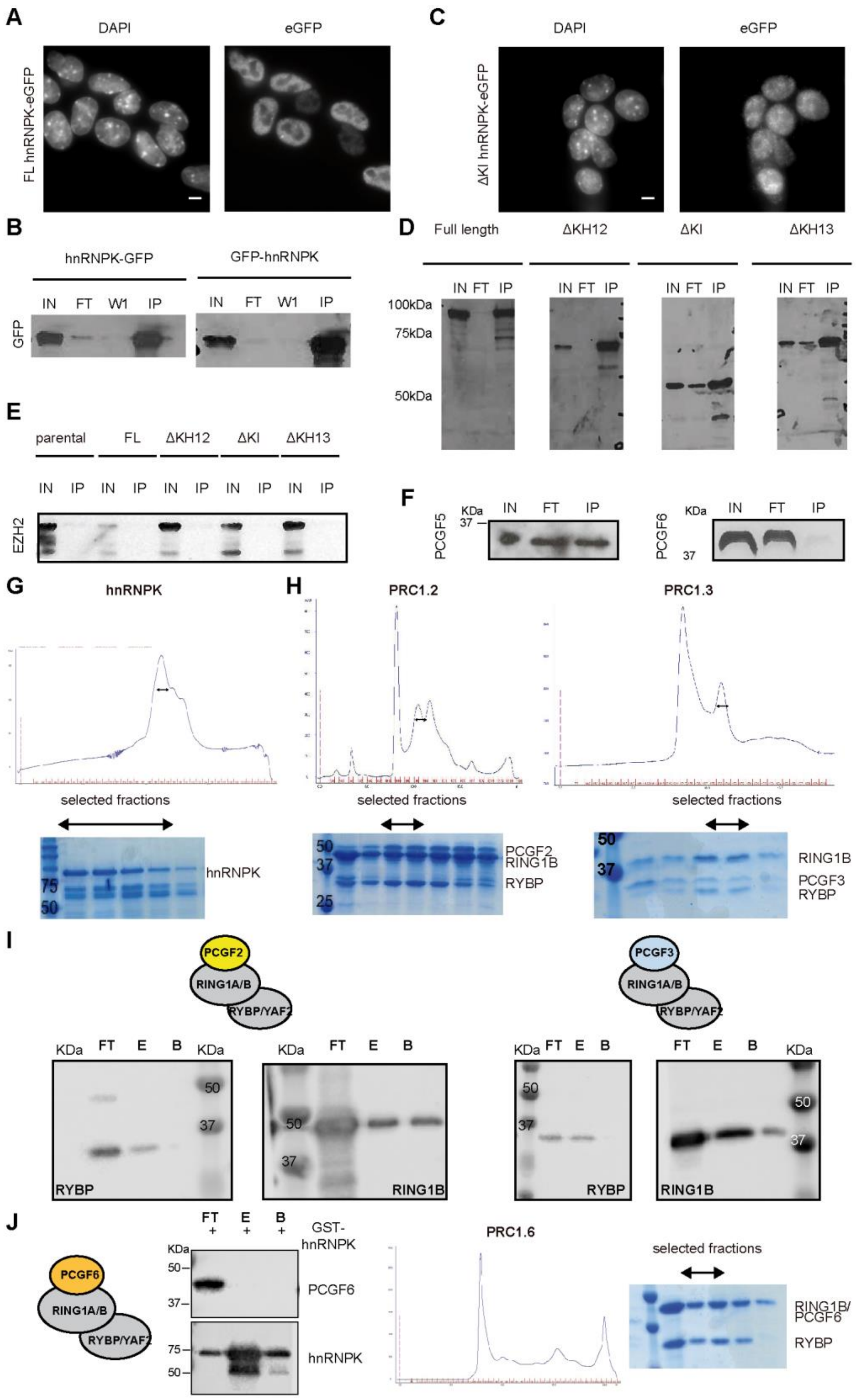
A
DAPI
eGFP
FL hnRNPK-eGFP
C
DAPI
eGFP
ΔKI hnRNPK-eGFP
B
hnRNPK-GFP
GFP-hnRNPK
IN FT W1 IP
IN FT W1 IP
GFP
D
Full length
ΔKH12
ΔKI
ΔKH13
IN FT IP
100kDa
75kDa
50kDa
E
parental
FL
ΔKH12
ΔKI
ΔKH13
IN IP IN IP IN IP IN IP IN IP
EZH2
F
KDa
37
IN FT IP
PCGF5
PCGF6
37
G
hnRNPK
selected fractions
hnRNPK
75
50
H
PRC1.2
PRC1.3
selected fractions
50
37
25
PCGF2
RING1B
RYBP
RING1B
PCGF3
RYBP
I
PCGF2
RING1A/B
RYBP/YAF2
PCGF3
KDa FT E B KDa
50
37
RYBP
RING1B
J
PCGF6
RING1A/B
RYBP/YAF2
FT E B
GST-hnRNPK
KDa
50
37
75
PCGF6
hnRNPK
PRC1.6
selected fractions
RING1B/ PCGF6
RYBP

Figure S6. Related to Figure 6. hnRNPK interacts with PCGF3/5-PRC1.

(A) Example illustrating detection of hnRNPK-eGFP expression in stable mESC cell line. DNA was counterstained with DAPI. Images represent single stacks. Scale bar is 5 μm.

(B) IP of C- and N-terminus eGFP tagged hnRNPK mESCs using a GFP antibody. 0.1% of the input (IN), 0.1% of the flow-through (FT), 0.1% of the first wash (W1), and 10% of the pull down (IP) were loaded.

(C) As in (A) for ΔKIhnRNPK-eGFP transfected mESC cell line.

(D) IP of hnRNPK and deletion constructs in mESCs using a GFP antibody. 0.1% of the input (IN), 0.1% of the flow-through (FT), and 10% of the pull down (IP) were loaded for each of the named constructs.

(E) Control CoIP showing PRC2 protein EZH2 does not interact with hnRNPK or mutant hnRNPK proteins. Loading was as in (D).

(F) CoIP of PCGF5 by hnRNPK-eGFP. PCGF6 was used as a negative control (see also below). Loading was as in (D).

(G and H) Examples of gel filtration of recombinant hnRNPK-GST (G) and PCGF2-PRC1 and PCGF3-PRC1 (H). A280 traces are shown above with CBB stained gels highlighting proteins of interest below.

(I) Western blots illustrating presence of RING1B and RYBP in the pull-down of recombinant PCGF3-PRC1 but not PCGF2-PRC1, using recombinant GST-hnRNPK. 0.1% of flow through (FT), 20% of eluate (E) and 33% of bead bound (B) were loaded.

(J) Purification of PRC1.6 and western blot showing absence of interaction with hnRNPK-GST as in (G-H) above, and in Figure 6C. Loading of the western blot was as in Figure 6C.

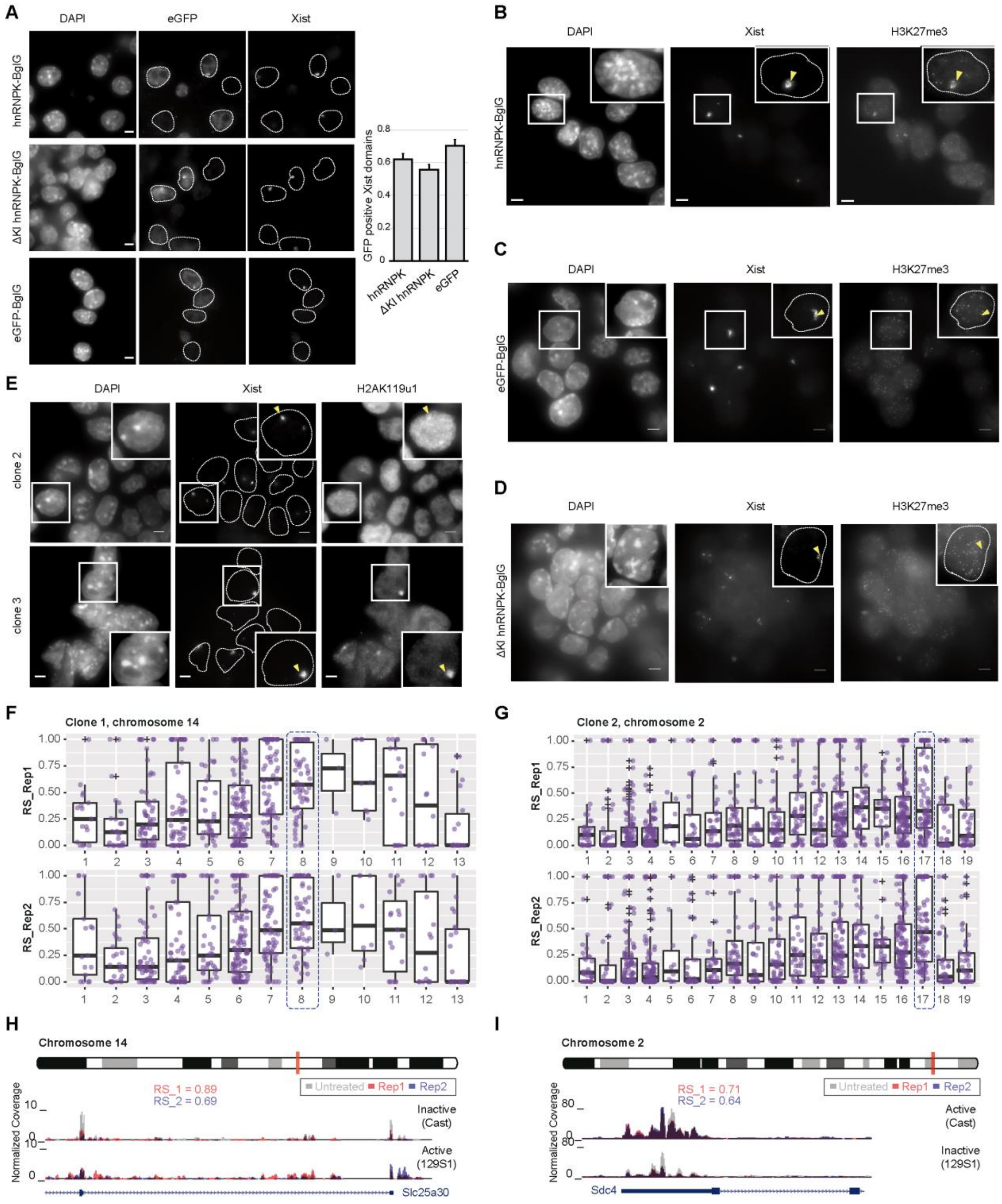

Figure S7. Related to Figure 7. Tethering hnRNPK is sufficient for Xist-dependent Polycomb recruitment

(A) Examples showing ImmunoFISH analysis of Xist RNA and eGFP-BglG fusion proteins 24 h after induction, illustrating that each of the named constructs accumulates over the

inactive chromosome. Wide-field images are average of 6 consecutive z-sections. DNA was counterstained with DAPI. Scale bar is 5 μm. Bar blots represent quantification for individual cell-lines, error bars represent sd across 3 biological replicates (n>100).

(B-D) Examples of ImmunoFISH detection of Xist and H3K27me3 with constructs expressing BglG-hnRNPK-eGFP (B), BglG-eGFP (C), BglG-ΔKIhnRNPK-eGFP (D). Wide-field images represent average of 6 consecutive z-sections. Arrows indicate Xist domains. Individual cells are shown enlarged as individual z-sections. Scale bar is 5 μm.

(E) Examples illustrating H2AK119u1 foci were detected in two independent cell lines (clone 2 and 3), expressing the BglG-hnRNPK-eGFP construct. Wide-field images represent average of 6 consecutive z-sections. Arrows indicate Xist domains. Individual cells are shown enlarged as individual z-sections. Scale bar is 5 μm. DNA was counterstained with DAPI.

(F-G) RS distribution in continuous 10 Mb windows across chromosome 14 (F) and chromosome 2 (right). Purple dots represent the RS for each gene. Blue boxes indicate maximum RS window.

(H-I) UCSC genome browser tracks showing the *Slc25a30* locus (H) and *Sdc4* locus (I) after induction of FL Xist or XistΔXR-PID respectively. Grey tracks represent no-dox control. Red and blue are two biological replicates with 72 h dox treatment. The active and inactive allele are indicated. Chromosomal location of *Slc25a30* and *Sdc4* is indicated with a red bar on the chromosome ideogram above each example.

Supplemental Tables

Table S3. Related to STAR Methods. List of Oligonucleotides.

REAGENT or RESOURCE	SOURCE	IDENTIFIER
**hnRNPK_F, LIC** tacttccaatccATGGAGACCGAACAGCCAGA	This study	LIC cloning
**hnRNPK_R, LIC** tatccacctttactTTAGAATCCTTCAACATCTGCAT	This study	LIC cloning
**hnRNPK_F1, LIC** tacttccaatccATGGAAATTCTGAAGAAAATCAT	This study	LIC cloning
**hnRNPK_F2, LIC** tacttccaatccATGAAACCTGATAGGGTTGTAGA	This study	LIC cloning
**hnRNPK_F3, LIC** tacttccaatccATGGGGTTCAGTGCTGATGAAAC	This study	LIC cloning
**hnRNPK_F4, LIC** tacttccaatccATGGAACCACAGGGTGGTTCTG G	This study	LIC cloning
**hnRNPK_R2, LIC** tacttccaatccATGTTGACCAGGAAGATTAAAC	This study	LIC cloning
**MluIPacI_F, LIC** tacttccaatccGGAGAATTCGAGCTCGGT	This study	LIC cloning
**MluIPacI_R, LIC** tatccacctttactTTATTGCCAGAGTTTAAT	This study	LIC cloning
**BglG_F, Gibson assembly** tcatcattttggcaaagaattccATGAACATGCAAATCA CCAAA	This study	Gibson assembly
**BglG_R, Gibson assembly** ggattggaagtacaggttctccACGGTGGCGACCGGT AGCAA	This study	Gibson assembly
**ColA1_CrisprF_T1 , gRNA** caccgTAGAAGGGGCCTTCTAATGC	This study	CRISPR HR
**ColA1_CrisprF_T2, gRNA** caccGAGGTTCATGAGCCCTCAAA	This study	CRISPR HR
**ColA1_CrisprF_T3, gRNA** caccGCCCCTTCTATACTAAATTA	This study	CRISPR HR
**MEDS-A, Tn5 assembly** Alexa594- GTCTCGTGGGCTCGGAGATGTGTATAAGAG ACAG	This study	Tn5 assembly
**MEDS-B, Tn5 assembly** Alexa594- TCGTCGGCAGCGTCAGATGTGTATAAGAGA CAG	This study	Tn5 assembly
**MEDS-REV, Tn5 assembly** Phos-CTGTCTCTTATACACATCT	Chen et al., 2016	Tn5 assembly

Table S4. Related to STAR methods. List of Antibodies.

REAGENT or RESOURCE	SOURCE	IDENTIFIER
H2AK119u1, Rabbit, IF	Cell Signaling	cat#8240
H3K27me3, Mouse, IF	Active Motif	cat#61017

GFP, Chicken, IP, IF	Abcam	cat#ab13970
GFP, Rabbit, IF	Abcam	cat# Ab290
RYBP, Rabbit, IF	Millipore	cat# AB3637
RING1B, Mouse, WB, IF	Gift from H. Koseki	N/A
RYBP, Rabbit, WB	Millipore	cat# AB3637
EZH2, Rabbit, WB, IF	Cell Signaling	cat# 5246
EED, Mouse, WB, IF	Gift from A. Otte	N/A
Histone H3, Rabbit, WB	Abcam	cat# ab1791
hnRNPK, Rabbit, WB, IP	NEB	cat# 4675
Neutravidin-HRP, Rabbit, WB	Life Technologies	cat# 31030
Anti-mouse Ig, HPR, Donkey, WB	Amersham	cat# NA934V
Anti-mouse Ig, HPR, Donkey, WB	Amersham	cat# NA931V
Alexa 568 anti-mouse IgG, Goat, IF	Life Technologies	cat# A11031
Alexa 488 anti-mouse IgG , IF	Life Technologies	cat# A11029
Alexa 568 anti-rabbit IgG, Goat, IF	Life Technologies	cat# A11034
Alexa 488 anti-rabbit IgG, Goat, IF	Life Technologies	cat# A11008
Alexa 488 anti-chicken IgG, Goat , IF	Life Technologies	cat# A11039
