# Supplementary material for: hnRNPK Recruits PCGF3/5-PRC1 to the Xist RNA B-Repeat to Establish Polycomb-Mediated Chromosomal Silencing
Source: Mol Cell. 2017 Dec 7;68(5):955–969.e10. doi: 10.1016/j.molcel.2017.11.013 (PMC5735038; doi:10.1016/j.molcel.2017.11.013)
Supplement: Document S2. Article plus Supplemental Information [file mmc4.pdf]

Article

Molecular Cell

hnRNPK Recruits PCGF3/5-PRC1 to the Xist RNA B-Repeat to Establish Polycomb-Mediated Chromosomal Silencing

Graphical Abstract

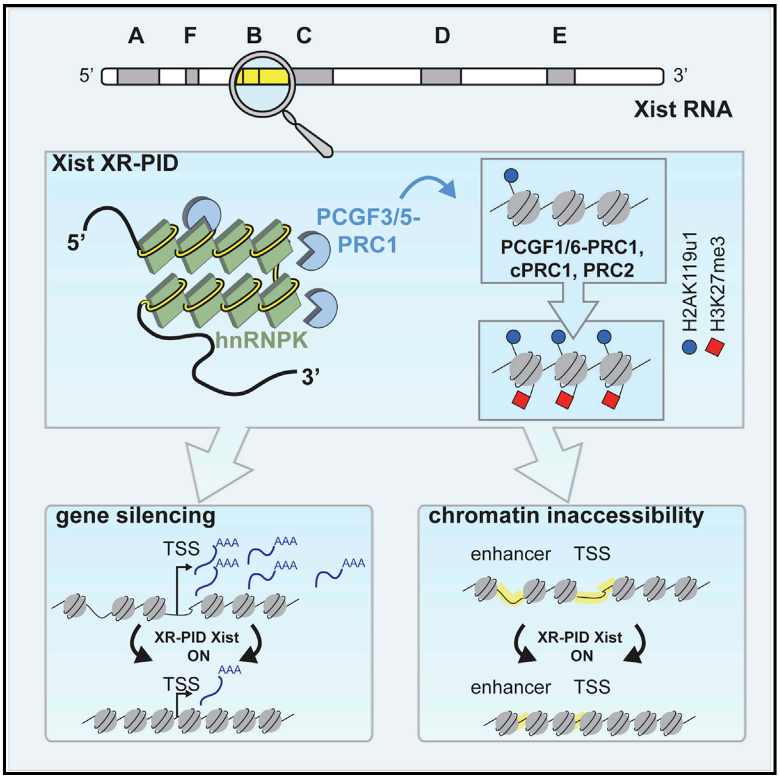

Highlights

- A 600 nt element in Xist RNA, XR-PID, is required for Polycomb recruitment
- Deletion of XR-PID abrogates Xist-mediated chromosome silencing
- hnRNPK binds XR-PID to recruit the Polycomb-initiating complex PCGF3/5-PRC1
- Tethering hnRNPK to Xist RNA bypasses the requirement for XR-PID

Authors

Greta Pintacuda, Guifeng Wei, Chloë Roustan, ..., Benoît Moindrot, Tatyana B. Nesterova, Neil Brockdorff

Correspondence

neil.brockdorff@bioch.ox.ac.uk

In Brief

This study advances our understanding of the molecular mechanism of X chromosome inactivation in mammals, defining XR-PID, the critical element in Xist RNA that recruits Polycomb complexes to the inactive X chromosome, and further demonstrating that the RNA binding protein hnRNPK bridges XR-PID with the initiating Polycomb complex, PCGF3/5-PRC1.

Pintacuda et al., 2017, Molecular Cell 68, 955–969
December 7, 2017 © 2017 The Authors. Published by Elsevier Inc.
https://doi.org/10.1016/j.molcel.2017.11.013

hnRNPK Recruits PCGF3/5-PRC1 to the Xist RNA B-Repeat to Establish Polycomb-Mediated Chromosomal Silencing

Greta Pintacuda,[1,7] Guifeng Wei,[1,7] Chloë Roustan,[1] Burcu Anil Kirmizitas,[1] Nicolae Solcan,[1,4] Andrea Cerase,[1,5] Alfredo Castello,[2] Shabaz Mohammed,[3] Benoît Moindrot,[1,6] Tatyana B. Nesterova,[1] and Neil Brockdorff[1,8,*]
[1]Developmental Epigenetics
[2]Posttranscriptional Networks in Infection and Cell Cycle Progression
[3]Proteomics Technology Development and Application
Department of Biochemistry, University of Oxford, South Parks Road, Oxford OX1 3QU, UK
[4]Present address: Heptares Therapeutics, BioPark, Broadwater Road, Welwyn Garden City AL7 3AX, UK
[5]Present address: EMBL Monterotondo, Adriano Buzzati-Traverso Campus, Via Ramarini 32, 00015 Monterotondo, Italy
[6]Present address: Institute for Integrative Biology of the Cell (I2BC), CEA, CNRS, Université Paris Sud, University Paris-Saclay, 1 Avenue de la Terrasse, 91198 Gif-sur-Yvette, France
[7]These authors contributed equally
[8]Lead Contact
*Correspondence: neil.brockdorff@bioch.ox.ac.uk
https://doi.org/10.1016/j.molcel.2017.11.013

SUMMARY

The Polycomb-repressive complexes PRC1 and PRC2 play a key role in chromosome silencing induced by the non-coding RNA Xist. Polycomb recruitment is initiated by the PCGF3/5-PRC1 complex, which catalyzes chromosome-wide H2A lysine 119 ubiquitylation, signaling recruitment of other PRC1 complexes, and PRC2. However, the molecular mechanism for PCGF3/5-PRC1 recruitment by Xist RNA is not understood. Here we define the Xist RNA Polycomb Interaction Domain (XR-PID), a 600 nt sequence encompassing the Xist B-repeat element. Deletion of XR-PID abolishes Xist-dependent Polycomb recruitment, in turn abrogating Xist-mediated gene silencing and reversing Xist-induced chromatin inaccessibility. We identify the RNA-binding protein hnRNPK as the principal XR-PID binding factor required to recruit PCGF3/5-PRC1. Accordingly, synthetically tethering hnRNPK to Xist RNA lacking XR-PID is sufficient for Xist-dependent Polycomb recruitment. Our findings define a key pathway for Polycomb recruitment by Xist RNA, providing important insights into mechanisms of chromatin modification by non-coding RNA.

INTRODUCTION

X inactivation is an epigenetic mechanism that evolved in mammals to equalize the dosage of X-linked genes in XX females relative to XY males. One of the two X chromosomes in early XX embryos is randomly selected and modified to form a transcriptionally repressed heterochromatic structure, the Barr body. Once established, the inactive state is stably inherited through subsequent cell divisions (Lyon, 1961).

Key features of the inactive X chromosome (Xi) include the acquisition (or loss) of specific histone post-translational modifications, DNA methylation of X-linked gene promoters, and changes in the higher-order chromosome folding, resulting in compaction and inaccessibility of the underlying chromatin (reviewed in Gendrel and Heard, 2014). The establishment of Xi modifications is initiated by a 17 kb long non-coding (lnc) RNA, Xist (X Inactive Specific Transcript), which localizes in *cis* along the length of the Xi elect (reviewed in Cerase et al., 2015). Accordingly, the X inactivation model serves as an important paradigm for understanding the role of chromatin modifications and non-coding RNA in the regulation of gene expression in development.

A key goal toward understanding the mechanism of X inactivation has been to define chromatin-modifying factors that are directly recruited by Xist RNA. The best-studied example to date is the Polycomb system, comprising the Polycomb-repressive complexes PRC1 and PRC2. PRC1 and PRC2, respectively, catalyze the histone modifications H2A lysine 119 ubiquitylation (H2AK119u1) and H3 lysine 27 di-/tri-methylation (H3K27me2/3), both of which are highly enriched on Xi (de Napoles et al., 2004; Plath et al., 2003; Silva et al., 2003; Wang et al., 2001). Early studies proposed that PRC2 complexes are directly bound by the A-repeat of Xist RNA (Zhao et al., 2008), a critical element required for chromosome silencing (Wutz et al., 2002). PRC1 recruitment was attributed to recognition of PRC2-mediated H3K27me3 by the canonical PRC1 subunit CBX according to the classical model for hierarchical recruitment of Polycomb complexes (Cao et al., 2002). However, building on the recent discovery that both PRC1 and PRC2 complexes can bind to pre-existing H2AK119u1 (Arrigoni et al., 2006; Blackledge et al., 2014; Cooper et al., 2014, 2016; Kalb et al., 2014), we have found that a specific non-canonical PRC1 complex, PCGF3/5-PRC1, initiates Xist-dependent Polycomb recruitment

This is an open access article under the CC BY license (http://creativecommons.org/licenses/by/4.0/).

(Almeida et al., 2017). Thus, H2AK119u1 catalyzed by PCGF3/5-PRC1 recruits PRC2, and also other non-canonical PRC1 complexes in response to accumulation of Xist RNA. Deletion of PCGF3/5-PRC1 results in female-specific embryo lethality and attenuates Xist-mediated silencing (Almeida et al., 2017).

An important question that remains unresolved is how Xist RNA recruits PCGF3/5-PRC1 and whether this is direct or mediated by co-factors. In this study, we map the critical sequence for Polycomb recruitment to a 600 nt element, the Xist RNA Polycomb Interaction Domain (XR-PID), which encompasses the Xist RNA B-repeat. We show that XR-PID is required for Xist-dependent deposition of H2AK119u1 and H3K27me3, efficient Xist-mediated gene silencing, and inactive chromosome inaccessibility. We identify hnRNPK as the critical XR-PID binding protein that directs the recruitment of PCGF3/5-PRC1. Conclusively, we demonstrate that in the absence of XR-PID, synthetic tethering of hnRNPK is sufficient for Polycomb recruitment by Xist RNA.

RESULTS

XR-PID—A Minimal Region of Xist RNA Required for Polycomb Recruitment

Previous work defined a 3.9 kb region of mouse Xist RNA, designated XN, required for recruitment of PRC1 and PRC2 (da Rocha et al., 2014; Almeida et al., 2017). The XN region includes three tandem repeat sequence clusters, the F, B, and C repeats (Figure 1A). Analysis of Xist sequences in different mammalian species indicated that the B-repeat, being highly conserved (Nesterova et al., 2001), is a candidate for mediating Polycomb recruitment. To test this possibility, we deleted a 0.6 kb region spanning the B-repeats in a doxycycline inducible Xist transgene. The deletion, XistΔXEv, encompasses the entire B-repeat array and a small part of the C-repeat located immediately downstream (Figures 1A and S1A). Stable cell lines were derived by transfecting the XistΔXEv or full-length (FL) Xist into P4D7, a *Mus domesticus* (129S1) x *Mus castaneus* F1 hybrid mouse embryonic stem cell (mESC) line (Almeida et al., 2017). Use of a hybrid genetic background facilitated analysis of Xist-mediated silencing (see below). Xist transgene expression was induced concurrent with initiation of mESC differentiation, in this case for 24 hr. ImmunoFISH analysis revealed complete loss of Xist-dependent recruitment of H2AK119u1 and H3K27me3 deposition (Figures 1B, 1C, and S1B). Enrichment of PRC1 and PRC2 subunits was also undetectable, as determined by immunofluorescence (IF) analysis (Figures S1D and S1E). These findings were confirmed using an independent XistΔXEv mESC cell line (data not shown). Henceforth we refer to this critical element as the Xist RNA Polycomb Interaction Domain (XR-PID).

To further define the XR-PID, we established a simplified assay, making use of EvXist, a short form of Xist RNA that corresponds to the first 3.9 kb of Xist exon I, and which encompasses the A-, F-, and B-repeat and a small part of the C-repeat present in XR-PID (Figure 1D). A previous study demonstrated that EvXist is sufficient to form Xist RNA domains, albeit less robustly than FL-Xist RNA (Wutz et al., 2002). We obtained similar results and found that EvXist recruits both PRC1 and PRC2, as determined by IF for H2AK119u1 and H3K27me3, respectively (Figures 1E, 1F, and S1C). We then analyzed a series of modified EvXist constructs, as summarized in Figure 1D. Deletion of the critical XR-PID sequence abolished PRC1/PRC2 recruitment, again with no effect on Xist RNA domain formation (Figures 1D–1F and S1C). Replacement of the XR-PID with an inverted fragment also abolished Polycomb recruitment (Figures 1D and 1F), indicating that the observed effect is not attributable to a reduction in the spacing of flanking elements. Deletions of EvXist spanning other regions, notably the F-repeats, did not affect Polycomb recruitment (Figures 1D and 1F). Deletions spanning the A-repeat, or a short region between the A-repeat and F-repeat (Figure 1D), disrupted Xist localization and therefore could not be tested for Polycomb recruitment (data not shown).

The XR-PID has a 0.3 kb proximal region comprising 32 tandem copies of the B-repeat motif, and a 0.3 kb distal region spanning most of the first three of 14 copies of the C-repeat (Figure S1A). We therefore analyzed the effect of deleting the proximal or distal XistΔXEv regions in the context of the EvXist construct (Figure 1D). As shown, deletion of the proximal region entirely abolished Polycomb recruitment (Figure 1F). Conversely, Polycomb recruitment occurred, albeit at a moderately reduced level, in the absence of the distal region (Figure 1F). These observations indicate that the B-repeat array has a central role in Polycomb recruitment.

XR-PID Is Required for Xist-Mediated Chromosome Silencing

We went on to assess the contribution of the XR-PID to Xist-mediated silencing by comparing allelic expression ratios in cell lines expressing either FL-Xist, XistΔXR-PID, XistΔXN (the large deletion previously shown to abolish Polycomb recruitment; da Rocha et al., 2014; Almeida et al., 2017), or XistΔSX (a deletion of the A-repeat element previously shown to be required for Xist-mediated silencing; Wutz et al., 2002). To account for variations in silencing related to different transgene integration sites (each mESC line has a unique integration event that can occur on any chromosome), we analyzed replicates for two independent cell lines for each of the different constructs (only a single line was analyzed for XistΔSX). We applied 4-thiouridine labeling followed by sequencing (4sU-seq) (Rabani et al., 2011), to enrich for nascent RNA in order to exploit *Mus castaneus* versus 129S1 single nucleotide polymorphisms (SNPs) in introns. Typically, we observed ~40% of reads overlapping with introns (data not shown). To quantify Xist-mediated silencing, we developed a computational method to generate a repression score (RS) for each gene, or a median RS value for genes within a defined window or whole chromosome, based on normalized allelic ratios (see materials and methods). RS analysis of 4sU-seq data revealed the chromosome and allele on which the Xist transgene was integrated (see, for example, Figure 2A), verified in each case by DNA FISH analysis of metaphase spreads (Figures S2A and S2B).

Time course experiments over 24–72 hr using a cell line expressing FL-Xist RNA indicated that chromosome silencing is highly correlated between individual time-points (Figures S2C–S2E). There was a marginal increase in repression over

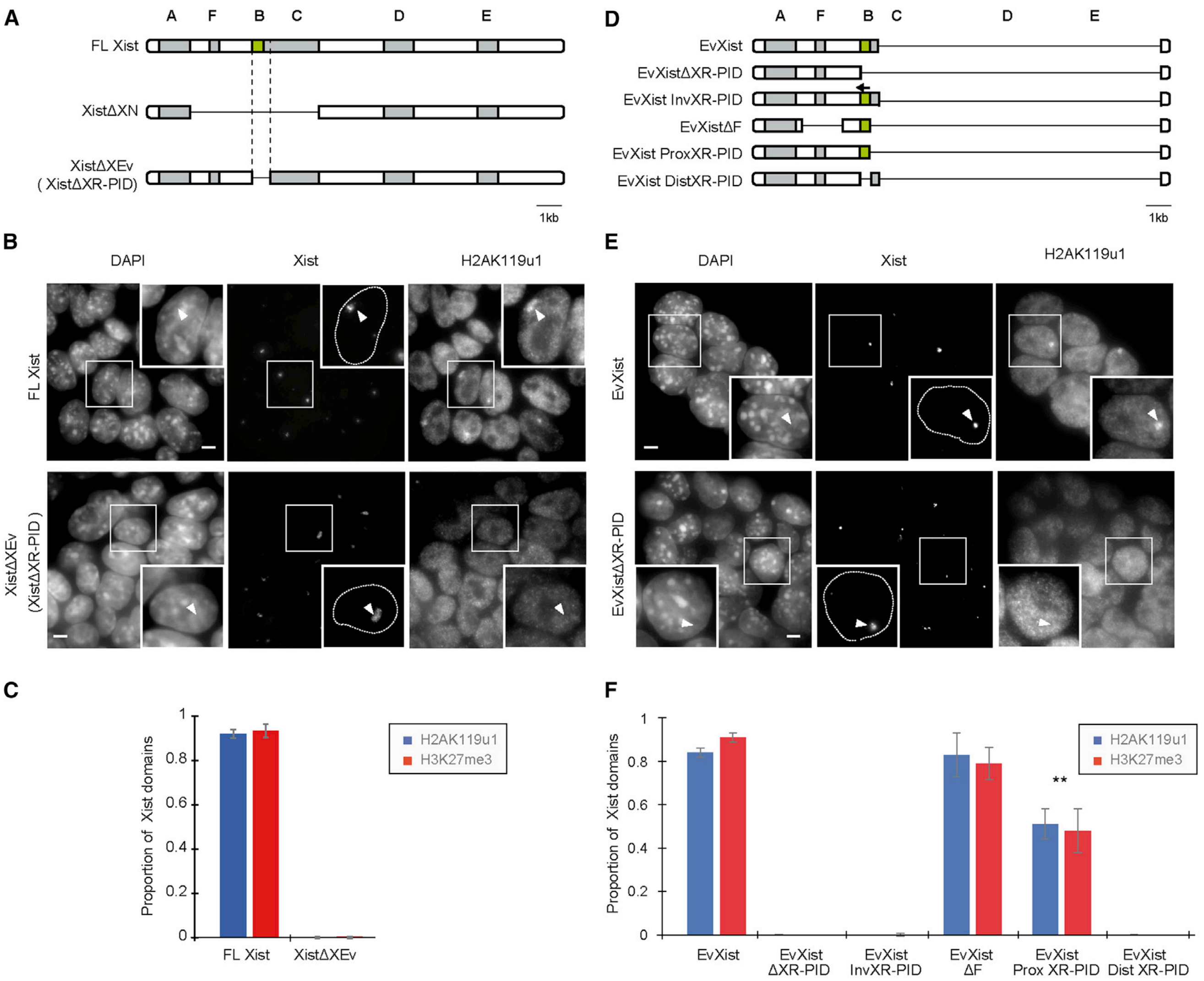

Figure 1. A 0.6 kb Element, XR-PID, Mediates Polycomb Recruitment by Xist RNA

(A) Schematic illustrating the XistΔXEv deletion (encompassing XR-PID) relative to FL-Xist. The location of Xist tandem repeats A–F is indicated. The XistΔXN transgene described previously is shown for comparison.

(B) Examples of immunoFISH analysis illustrating presence or absence of H2AK119u1 foci (arrowheads), following 24 hr induction of Xist transgenes. Images are stacks of six consecutive Z sections with insets showing a single section. Nuclei outlines determined from DAPI stain are indicated with dashed line in inset. Scale bar indicates 5 μm.

(C) Quantitative analysis of a single FL-Xist and XistΔXEv cell line based on scoring Xist RNA domains for presence or absence of H2AK119u1 or H3K27me3. Error bars represent SD for at least three biological replicates with a sample of n > 100 for each replicate.

(D) As in (A). Green shading highlights B-repeat element.

(E) As in (B).

(F) As in (C). **p < 0.001 relative to FL-Xist (2 tailed Student's t test).

time, with maximal levels seen at 72 hr (Figures S2C and S2D), and this time point was therefore used for subsequent experiments. We went on to compare chromosome silencing induced by FL-Xist, XistΔXN, and XistΔXR-PID (Figures 2B–2E). Silencing by FL-Xist was, as expected, efficient, and extended along the length of the chromosome. An example, P4D7F4, in which the transgene integrated on the *Mus castaneus* allele of chromosome 3, showed significant repression of the majority of genes (~96%; 630 out of 659) (Figures 2B and 2C). XistΔXN and XistΔXR-PID lines, however, showed reduced repression efficiency. Thus, in a representative XistΔXR-PID cell line (Figures 2B and 2C), significant repression was seen for approximately half of the genes on the chromosome (~49%; 565 out of 1,144).

To quantify the silencing efficiency of different constructs, we compared the RS within defined windows in which highest levels of silencing were observed (Figure 2D). We further determined the proportion of genes with RS in the range 0.1–1 within the maximal 10 Mb window for each cell line (Figure 2E). The results

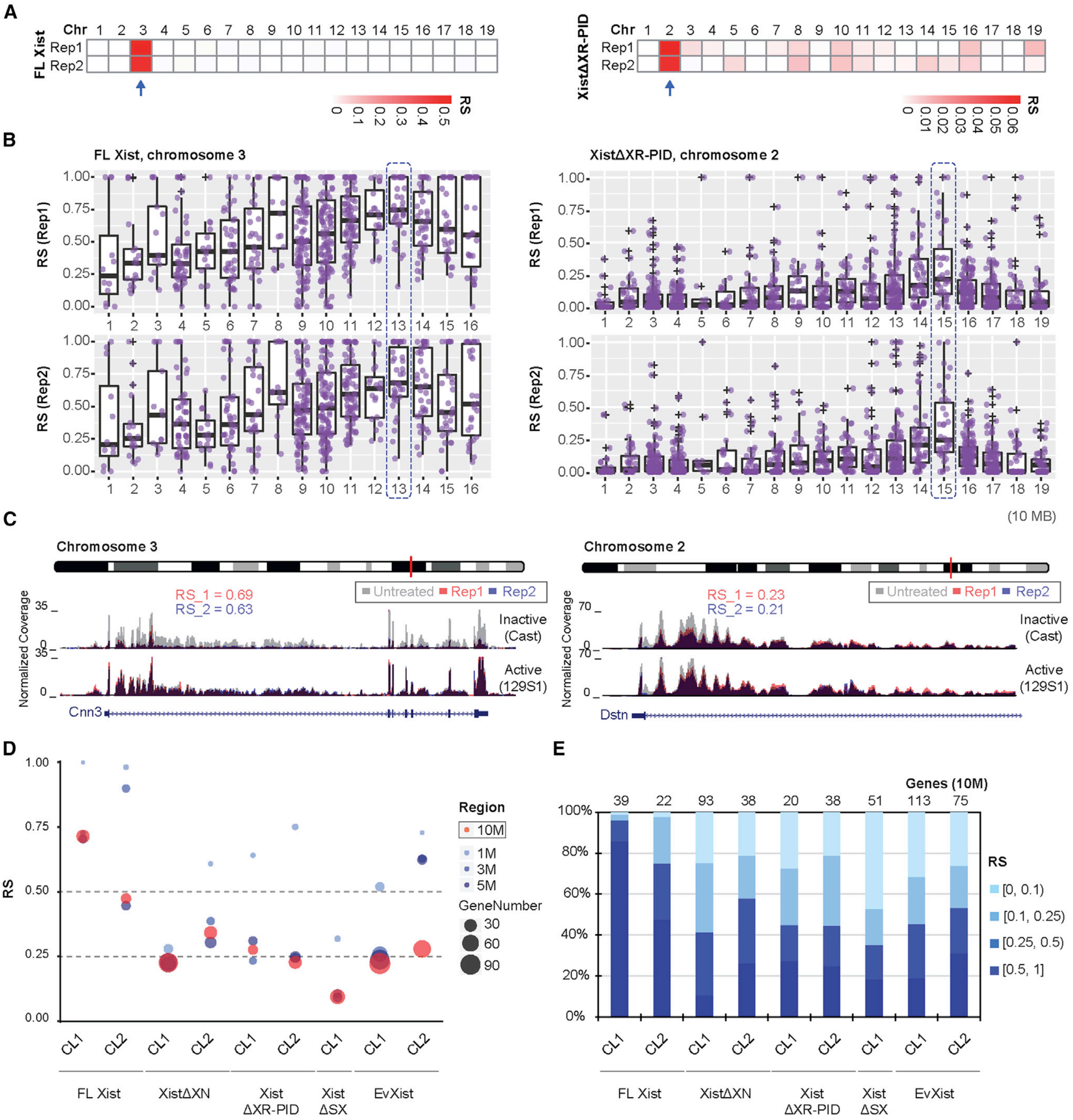

Figure 2. The XR-PID Element Contributes to Transcriptional Silencing Mediated by Xist RNA

(A) RS distribution across all autosomes in two biological replicates, for two cell lines expressing FL-Xist (left) versus XistΔXR-PID (right), following a 72 hr induction of the transgenes.

(B) RS distribution in continuous 10 Mb windows across chromosome 3 for FL-Xist (left) and chromosome 2 for XistΔXR-PID (right). Purple dots represent the RS for each gene. Blue boxes indicate maximum RS window.

(C) UCSC genome browser tracks showing the *Cnn3* locus (left) and *Dstn* locus (right) after induction of FL-Xist or XistΔXR-PID, respectively. Gray tracks represent no-dox control. Red and blue are two biological replicates with 72 hr dox treatment. The active allele (129S1) and inactive allele (Cast) are indicated. Chromosomal location of *Cnn3* and *Dstn* is indicated with a red bar on the chromosome ideogram above each example.

(D) Comparison of RS in windows centered on maximal RS for cells harboring FL-Xist and Xist transgenes as indicated. The color and area of circles indicate the size and gene number within the region, respectively.

(E) RS comparison (% of genes) in the 10 Mb of maximum silencing windows depicted in (B). RS range is as indicated.

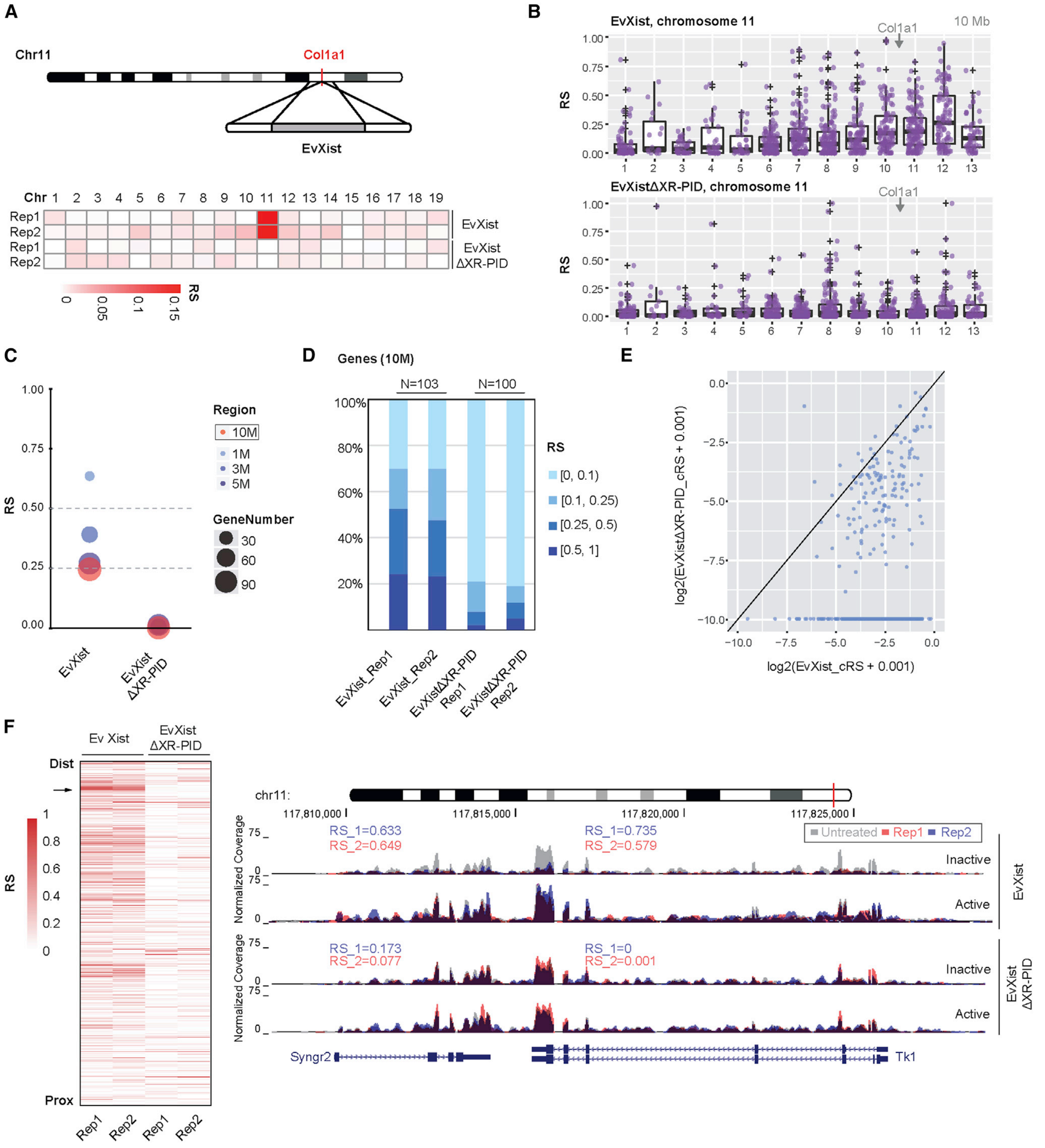

Figure 3. XR-PID-Mediated Silencing Analyzed using EvXist, a Truncated Xist Transgene

(A) Schematic for site-specific integration of a single copy of EvXist or EvXistΔXR-PID into the *Col1a1* locus (in red) on chromosome (chr) 11 (top). Heatmap shows the RS in each chromosome for both replicates (rep), using EvXist or EvXistΔXR-PID (bottom).

(B) RS distribution in continuous 10 Mb windows across chromosome 11, following 72 hr induction of single-copy EvXist (top) or EvXistΔXR-PID (bottom) transgenes. Purple dot represents the RS for each gene. The position of the *Col1a1* locus is indicated.

(C) RS comparison between EvXist and EvXistΔXR-PID in windows centered on the region of maximum silencing. The color and area of dots indicate the region size and gene number, respectively.

(legend continued on next page)

from these analyses clearly illustrate reduced silencing using XistΔXN and XistΔXR-PID compared to FL-Xist transgenes. Given that deletion of PCGF3/5-PRC1 attenuates silencing to a similar degree (Almeida et al., 2017), we infer that the observed effect is attributable to the requirement for XR-PID for Xist-dependent PRC1/PRC2 recruitment. For XistΔSX, in which the critical silencing element, the A-repeat, is deleted, we were unable to identify the transgene-bearing chromosome on the basis of allelic expression ratios, consistent with expectations. We did, however, identify the integration site of the single XistΔSX cell line using DNA FISH (Figure S2B), and here we were able to retrospectively discern a region with a low level of allelic repression (Figures 2D and 2E). This observation is consistent with a previous report indicating that XistΔSX transgenes repress to a limited degree (Pullirsch et al., 2010). Levels of induced Xist RNA using different constructs/time-points were comparable (Figures S2F and S2G), indicating that this variable does not contribute significantly to observed differences in silencing efficiency.

A similar analysis of cell lines carrying the truncated EvXist transgenes revealed chromosome-wide silencing, but at a reduced level, similar to XistΔXN/XistΔXR-PID (Figures 2D and 2E). For cell lines expressing EvXistΔXR-PID, we were unable to identify the transgene-bearing chromosome before DNA-FISH analysis (Figure S2B), indicating that silencing was strongly abrogated. To test this more definitively, we used homologous recombination to target a single copy of EvXist or EvXistΔXR-PID transgenes into the *Col1a1* homing site on chromosome 11 (Beard et al., 2006) in P4D7 mESCs, and performed 4sU-seq and RS analysis (Figures 3A and S3A). Because the EvXist and EvXistΔXR-PID transgenes are at the same site, we were able to derive a calibrated (c) RS score, which precisely accounts for the statistical significance of RS, over random mono-allelic expression of genes located on the same chromosome (see STAR Methods). We made use of cRS to directly compare EvXist and EvXistΔXR-PID-mediated silencing. For EvXist, 45% of genes (454 out of 1,010) were significantly repressed, with the majority (373 out of 454) being located on the distal half of chromosome 11 (Figure 3B). The cRS within the 10 Mb maximum silencing region was similar to the RS seen with EvXist random integrants (Figure 2D). However, for EvXistΔXR-PID, silencing was strongly reduced, with only 10% of genes (100 out of 1,010) being significantly repressed (Figures 3B–3F and S3B–S3D). Moreover, in the majority of cases, repression by EvXistΔXR-PID was at a lower level than that seen in EvXist-expressing cells (Figures 3E, S3C, and S3D). Examples of EvXist and EvXistΔXR-PID-mediated silencing of specific loci, *Syngr2-Tk1* and *Sept9*, located in the 10 Mb maximum silencing region, are illustrated in Figures 3F and S3E. Transgenic Xist RNA levels were similar (Figure S3F).

Deletion of XR-PID Abrogates Xist-Dependent Loss of Chromatin Accessibility

Polycomb complexes mediate gene silencing either directly, by inhibiting gene transcription (Stock et al., 2007; Zhou et al., 2008), or indirectly, by modulating higher-order chromatin structure or compaction (Eskeland et al., 2010; Isono et al., 2013; Lau et al., 2017). High-resolution mapping of Xist-dependent H3K27me3 shows equal enrichment over gene promoters, gene bodies, and intergenic regions (Calabrese et al., 2012; Marks et al., 2009; Pinter et al., 2012), which is difficult to reconcile with direct effects on transcription. With this in mind, we investigated whether expression of the Xist transgenes lacking the XR-PID element affected parameters associated with higher-order chromatin organization, specifically chromatin accessibility. Initially we measured chromatin accessibility using the Assay for Transposase-Accessible Chromatin followed by ImmunoFISH (ATAC-see) (Chen et al., 2016). Expression of FL-Xist transgenes established inaccessible chromatin domains (Figure 4A), as reported previously (Chen et al., 2016). In marked contrast, we failed to detect loss of chromatin accessibility in response to expression of the XistΔXR-PID transgene (Figure 4B). Equivalent results were obtained using cell lines with independent transgene integration sites, indicating that the difference relates to the functional properties of the different transgenes rather than the site of integration.

To increase the resolution of our analysis, we performed allelic ATAC-seq (Buenrostro et al., 2013; Giorgetti et al., 2016) on representative cell lines with either FL-Xist or XistΔXR-PID transgenes, located on chromosomes 3 and 2, respectively. As illustrated in Figures 4C–4F and S4A, FL-Xist expression significantly reduces chromatin accessibility at sites across the entire chromosome, similar to previous reports (Giorgetti et al., 2016). In contrast, expression of the XistΔXR-PID transgene resulted in little or no change in chromatin accessibility. This effect was evident for all key regulatory elements, promoters, enhancers, and CTCF-binding sites (Figures S4B–S4D). Together, these results suggest that Xist-dependent Polycomb activity is critical for chromatin compaction to facilitate transcriptional repression.

A Proteomic Screen Identifies hnRNPK as a B-Repeat RNA-Binding Protein

We went on to investigate the mechanism for Polycomb recruitment by the B-repeat element. Thus, we established a proteomic screening strategy using *in vitro*-transcribed and biotinylated RNA templates to identify RNA-binding proteins (RBPs) in mESC extracts that interact directly with Xist B-repeat RNA (Figures 5A, 5B, and S5A–S5E). We engineered a 0.8 kb construct that includes both the A-repeat and B-repeat elements, together with flanking sequences, and additionally, mutant constructs in which either the A-repeat or B-repeat was replaced with non-

(D) RS comparison (% of genes) between EvXist and EvXistΔXR-PID cell lines in the 10 Mb of maximum silencing windows depicted in (B). The RS was grouped into four categories, indicated by the graded color.

(E) Scatterplot showing the differences of the calibrated (c) RS for 454 repressed genes in EvXist and EvXistΔXR-PID. The axis represents log2-transformed cRS.

(F) Heatmap illustrating RS of each gene ranked by genomic coordinates on chromosome 11 for both biological replicates of EvXist and EvXistΔXR-PID cells (left). The *Syngr2-Tk1* locus, indicated by red lines, is shown in the upper chromosome ideogram. RS is indicated, and q value for both *Syngr2* and *Tk1* is 0 in EvXist cells and 0.036 and 0.611, respectively, in EvXistΔXR-PID cells. Data for no dox (untreated) and for two replicates (rep) of 72 hr induction (red and blue) are shown. Active (129S1) and inactive (Cast) alleles are labeled (right).

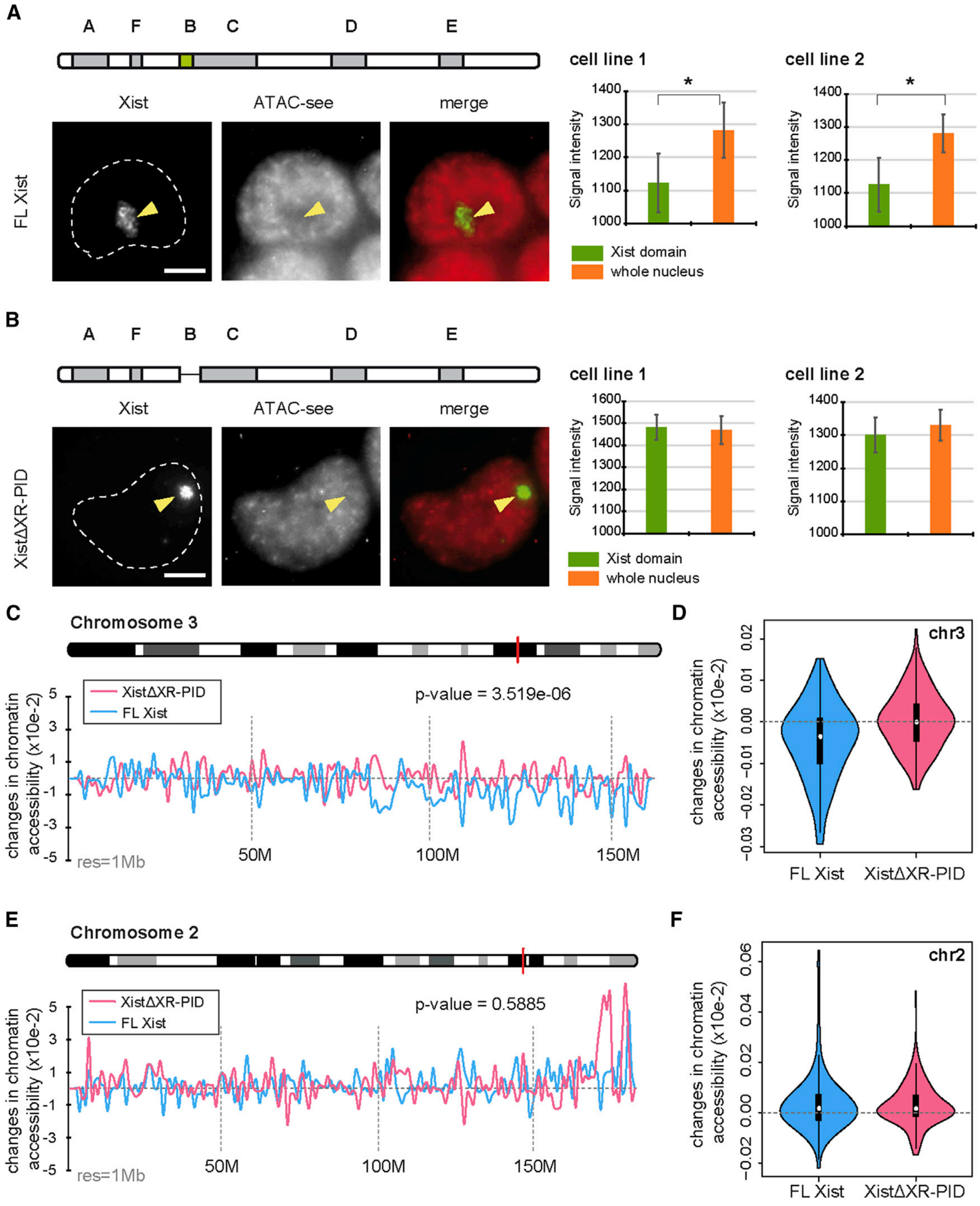

Figure 4. XR-PID Is Required for Reduced Chromatin Accessibility over Xist Domains

(A and B) Individual examples illustrating Xist RNA-FISH combined with ATAC-see. The nuclei (DAPI staining) are outlined with a dotted line with Xist domains indicated (arrowhead). Scale bar indicates 5 μm. Fluorescence intensity of the ATAC-see signal overlapping Xist and total nuclear signal were measured for two independent cell lines for FL-Xist (A) and XistΔXR-PID transgenes. Bar graphs represent average values for three biological replicates (n > 20) for each independent cell line. Error bars represent SD. *p < 0.05 (paired Student t test).

(C) Chromatin accessibility changes upon FL-Xist induction on chromosome 3. The red bar on the ideogram indicates maximum silencing regions. The chr3-wide accessibility changes from XistΔXR-PID expressing cells, in which the Xist transgene is on chr2, serves as a control. The p value was calculated using Mann-Whitney test.

(D) Chromatin accessibility changes in the presence (blue) or absence (red) of FL-Xist expression.

(E) Chromatin accessibility changes upon XistΔXR-PID induction on chromosome 2. Red bar on the ideogram indicates maximum silencing regions. The chr2-wide accessibility changes from FL-Xist transgene cells (chr3) provide a control. The p value was calculated using Mann-Whitney test.

(F) As in (D) for XistΔXR-PID.

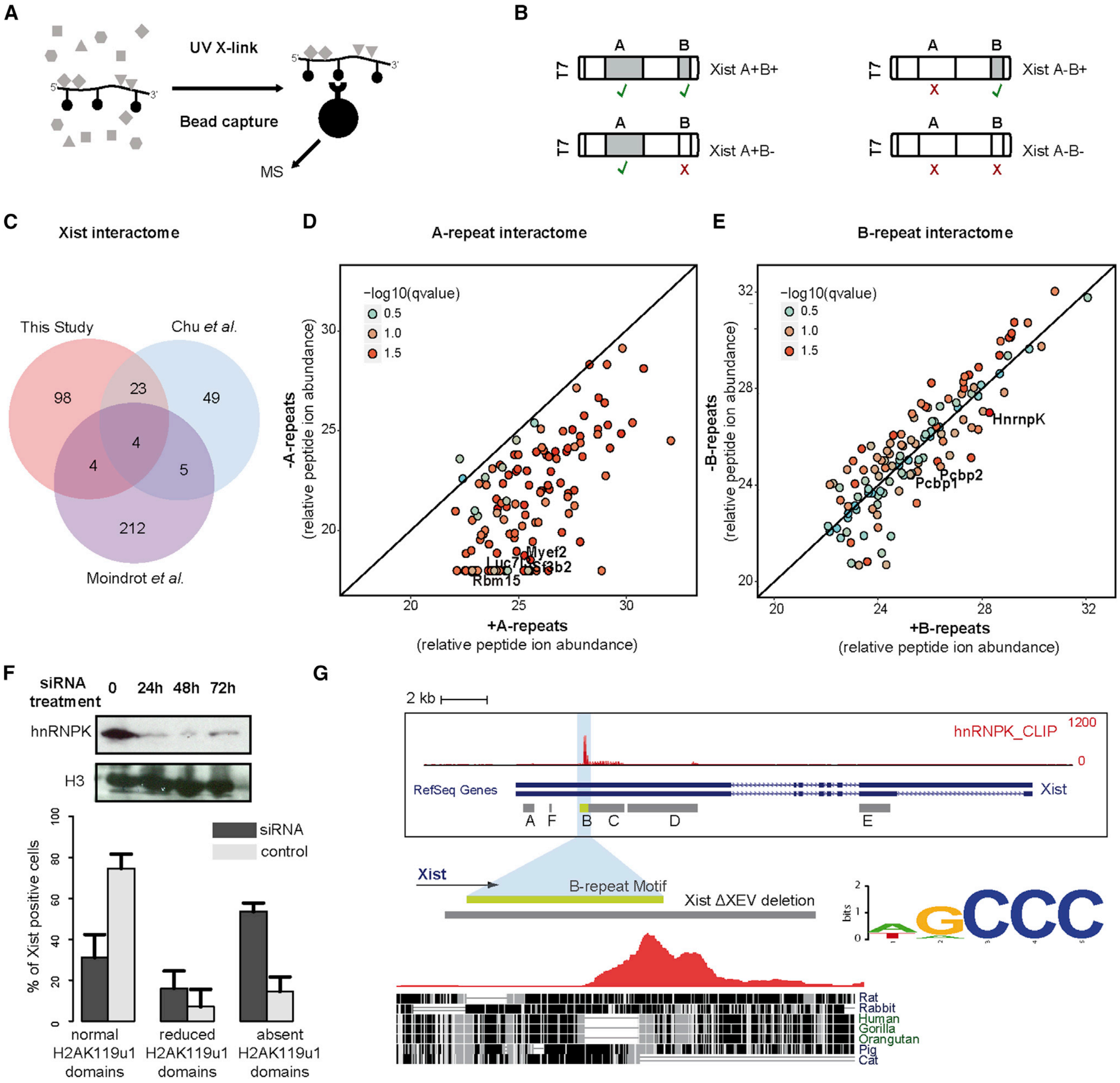

Figure 5. hnRNPK Binds Xist B-Repeat

(A and B) Schematic (A) of the proteomic screening strategy using different *in vitro* transcribed Xist constructs, indicated in (B). Proteins in nuclear extract (gray shapes) that bind *in vitro*-transcribed RNA with incorporated biotin (filled lollipop) were captured by UV cross-linking and then purified using streptavidin beads (large filled circle) prior to analysis by MS.

(C) Overlap with Xist-interacting partners identified in independent biochemical (Chu et al., 2015) and genetic (Moindrot et al., 2015) screens.

(D and E) Scatterplots representing abundance of factors interacting preferentially with the A-repeat (D) or the B-repeat (E) elements of Xist by quantitative MS. Q value was calculated across two biological replicates.

(F) Western blot shows levels of hnRNPK before and after 24, 48, and 72 hr siRNA treatment. Histone H3 was used as a loading control. Bar chart quantifies immunoFISH analysis of H2AK119u1 and Xist RNA co-localization in cells treated with hnRNPK or scrambled (control) siRNA. Data are from two independent siRNA transfections, each with three biological replicates (n > 50). Error bars represent SD across all biological replicates.

(G) UCSC track displaying hnRNPK iCLIP data (Cirillo et al., 2016) aligned to Xist RNA sequence. The correspondence with the XR-PID and specifically with the B-repeat core motif is highlighted in light blue. The most prominent peak of hnRNPK binding is enlarged below (red) and aligned to the genomic sequence of the XR-PID across several mammalian species. The consensus B-repeat core sequence spanning the region is also represented.

functional sequences. Thus, in the case of the A-repeat we used a synthetic mutant construct previously shown to disrupt A-repeat specific function (Wutz et al., 2002), whereas for the B-repeat we used the non-functional inverted B-repeat sequence (Figure 1D).

We performed an MS proteomic analysis to identify bound RBPs following UV crosslinking and purification of the biotinylated RNA with its covalently bound proteins. High-confidence interactors obtained using the unmodified template (Table S1) showed significant overlap with Xist-interacting proteins defined in prior *in vivo* proteomic (Chu et al., 2015) and genetic (Moindrot et al., 2015) screens (Figure 5C). We went on to define A-repeat- and B-repeat-specific binding proteins by performing label-free comparative MS analysis (Figures 5D and 5E). Thus, we identified several proteins that bound to RNA templates in an A-repeat-specific manner (Figure 5D; Table S1). Among these were four factors identified in Xist genetic and proteomic screens as above: Rbm15, an RBP that interacts specifically with the A-repeat to function in Xist-mediated silencing (Moindrot et al., 2015; Patil et al., 2016); the transcriptional repressor Myef2; and the splicing factors Luc7l3 and Sf3. We also identified proteins that bound preferentially to template RNA in the presence of functional B-repeat (Figure 5E; Table S1). Notably, we identified hnRNPK, an Xist-binding protein previously implicated both in Xist-mediated silencing and recruitment of PRC1 and PRC2 (Chu et al., 2015). hnRNPK binds preferentially to cytidine tracts in RNA (Swanson and Dreyfuss, 1988), which is a characteristic of the conserved short tandem B-repeat consensus (Figure S1A). Consistent with this finding, PCBP1/2, two related RNA-binding proteins with a preference for cytidine tracts were also among the factors that bind preferentially to B-repeat RNA (Figure 5E).

We went on to further validate hnRNPK as a candidate for mediating PRC1/PRC2 recruitment. Consistent with prior work, we found that RNAi-mediated knockdown of hnRNPK reduces H2AK119u1 deposition in response to induction of a FL Xist RNA transgene (Figures 5F and S5F). Additionally, a previously published iCLIP analysis of hnRNPK binding sites mapped a major peak in Xist exon I (Cirillo et al., 2016). Re-analysis of these data relative to the location of tandem repeats in Xist RNA reveals close overlap with the B-repeat element (Figure 5G). A low level of hnRNPK enrichment extends into the C-repeat region, consistent with our observation that the C-repeat elements present in XR-PID contribute to Polycomb recruitment to a limited degree (Figure 1F).

Biochemical Interaction of hnRNPK with PCGF3/5-PRC1

The aforementioned experiments suggest that hnRNPK plays an important role in Polycomb recruitment by the B-repeat of Xist RNA. hnRNPK is a multi-functional protein implicated in several processes, including chromatin modification, transcription, splicing, and translation (reviewed in Bomsztyk et al., 2004). Interaction studies have identified a large number of hnRNPK partner proteins, including the PRC2 protein EED (Bomsztyk et al., 2004). We were interested to determine if hnRNPK interacts with PRC1 proteins, notably the PCGF3/5-PRC1 complex that initiates Polycomb recruitment by Xist RNA (Almeida et al., 2017). To investigate this possibility, we performed co-immunoprecipitation (coIP) analysis in mESCs. Thus, we established mESC lines stably expressing eGFP-hnRNPK (Figures S6A and S6B) and then carried out coIP analysis using a high-specificity single-chain *Lama alpaca* antibody against eGFP to detect subunits of either PRC1 or PRC2. As illustrated in Figure 6A, RING1B and RYBP, core subunits of PRC1, co-immunoprecipitated with hnRNPK, whereas the PRC2 core subunits EED and EZH2 did not. This result accords with our recent findings linking PRC1 to Xist-dependent Polycomb recruitment (Almeida et al., 2017).

hnRNPK has three KH domains that mediate RNA-binding and a KI domain, located between KH2 and KH3 (Figure 6B), which has been implicated in binding to diverse interaction partners (Bomsztyk et al., 2004). To test which region of hnRNPK interacts with PRC1, we established mESC lines stably expressing eGFP-hnRNPK constructs with overlapping deletions (Figures S6C–S6E) and then performed coIP for RING1B. As shown in Figure 6B, the minimal region required for specific coIP of RING1B maps to the KI domain of hnRNPK.

We predicted that the interaction of hnRNPK and PRC1 would be specific to the PCGF3/5-PRC1 complex, required to initiate Polycomb recruitment by Xist RNA. Consistent with this idea, we were able to demonstrate coIP of PCGF5 with GFP-hnRNPK (Figure S6F). To further validate this finding, and to determine if the interaction is direct, we established a pull-down assay using recombinant proteins expressed in *E. coli*. Thus, we purified hnRNPK as a GST fusion protein (Figure S6G) and assembled PRC1 complexes with the core subunits RING1B and RYBP, and then either PCGF3, representative of PCGF3/5-PRC1, or PCGF2 and PCGF6, representative of other canonical and non-canonical PRC1 complexes (Figures S6H–S6J). Using stringent conditions, we observed robust interaction between hnRNPK and PCGF3-PRC1, but not PCGF2-PRC1 (Figure 6C) or PCGF6-PRC1 (Figure S6J). Given that RING1B and RYBP are present in all three recombinant PRC1 complexes, we conclude that specificity of the interaction with hnRNPK is conferred by the presence of the PCGF3 subunit.

Tethering hnRNPK to XistΔXR-PID Restores Polycomb Recruitment

Together, our results suggest that hnRNPK binds to XR-PID and then directly recruits PCGF3/5-PRC1 to initiate chromosome-wide Polycomb recruitment. To further test this model, we established an experimental system to complement the XR-PID deletion by synthetically tethering hnRNPK. Thus, we made use of the BglG/Bgl stem loop (SL) tether, which has been used previously to tag Xist RNA with mCherry for imaging experiments (Moindrot et al., 2015). We developed a construct for expressing BglG-hnRNPK-eGFP fusion protein, and as a control BglG-eGFP fusion protein, in P4D7 mESCs, co-transfected with an inducible XistΔXR-PID transgene tagged with an array of 18 copies of the BglSL (Figure 7A). We derived several independent cell lines expressing either BglG-eGFP or BglG-hnRNPK-eGFP. IF analysis of GFP demonstrated that both fusion proteins localize efficiently to single nuclear foci following induction of Xist RNA (Figure S7A).

To test the ability of the fusion proteins to mediate Polycomb recruitment, we analyzed co-localization of Xist transgene RNA and H2AK119u1 (Figures 7B–7D) or H3K27me3 (Figures 7C

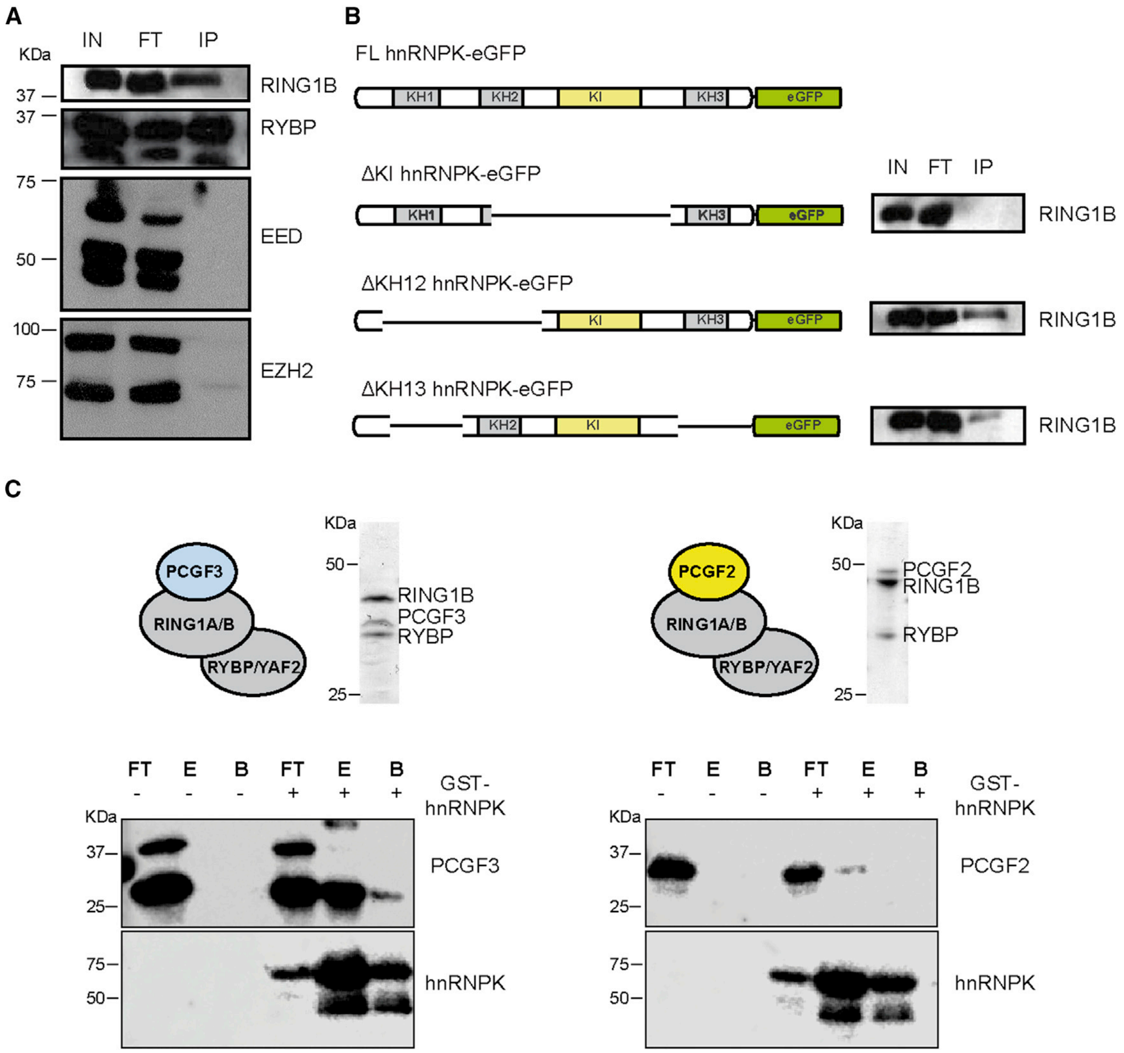

Figure 6. hnRNPK Interacts Directly with PCGF3/5-PRC1

(A) Western blots illustrating eGFP pull-down of PRC1 and PRC2 proteins (as indicated) in cells expressing hnRNPK-eGFP. Loaded were 0.1% of the input (IN), 0.1% of the flowthrough (FT), and 10% of the pull-down (IP).

(B) RING1B pull-down repeated using cell lines expressing hnRNPK-eGFP deletion variants as indicated in schematic. Loading is as in (A).

(C) Western blots illustrating pull-down of recombinant PCGF3-PRC1, but not PCGF2-PRC1, using recombinant GST-hnRNPK. A Coomassie brilliant blue-stained gel of the complexes used in the assay is shown above. Loaded were 0.1% of flowthrough (FT), 20% of eluate (E), and 33% of bead bound (B).

and S7B–S7D), 24 hr after transgene induction. Strikingly, localization of BglG-hnRNPK-eGFP fusion resulted in Xist-dependent deposition of both H2AK119u1 and H3K27me3 (Figures 7B, 7C, and S7B). Detection of H2AK119u1/H3K27me3 domains varied somewhat in different hnRNPK-BglG cell lines (Figure 7C), and this appeared to correlate with the relative size of Xist RNA domains (Figure S7E). Recruitment of the BglG-eGFP fusion protein alone did not lead to either H2AK119u1 (Figures 7B and 7C) or H3K27me3 (Figures S7C and 7C) deposition, also confirmed in independently derived mESC lines (Figure 7C).

As a further test of our findings, we engineered a deletion of the hnRNPK KI domain, implicated in interaction with PCGF3/5-PRC1 (Figure 6B), into the BglG-hnRNPK-eGFP fusion protein. Stable mESC lines generated by co-transfecting this construct, together with an inducible BglSL-XistΔXR-PID transgene, were then analyzed for eGFP localization (Figure S7A) and for Xist-dependent H2AK119u1 (Figures 7C and 7D) and H3K27me3 (Figures S7D and 7C). Polycomb recruitment to Xist domains was undetectable in independently derived mESC lines.

Finally, to determine if tethering hnRNPK to BglSL-XistΔXR-PID RNA enhances Xist-mediated silencing, we performed allelic 4sU RNA-seq analysis on two of these cell lines. As shown in Figures 7E, 7F, and S7F–S7I, the RS values obtained were similar to those found for FL-Xist and were significantly higher than with XistΔXR-PID Xist transgenes. Together, these results substantiate that hnRNPK binding to the B-repeat recruits PCGF3/5-PRC1 to initiate Xist-dependent recruitment of Polycomb complexes (Figure 7G).

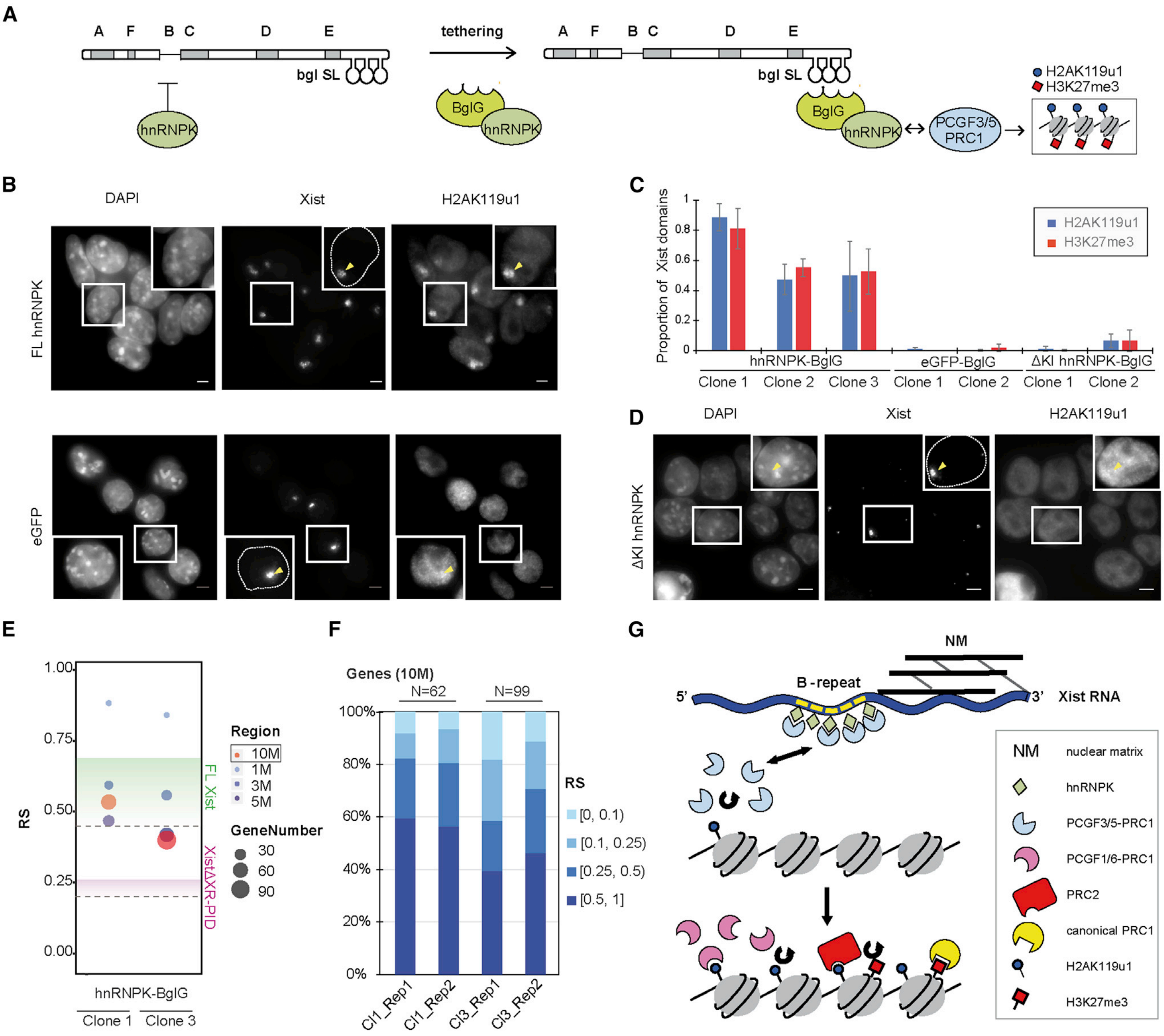

Figure 7. Tethering hnRNPK Is Sufficient for Xist-Dependent Polycomb Recruitment

(A) Schematic illustrating the hnRNPK-tethering experiment.

(B) Examples of ImmunoFISH detection of Xist RNA and H2AK119u1 using BglG-hnRNPK or BglG-eGFP. Wide-field images represent stacks of 10 consecutive Z sections. Arrows indicate Xist domains. Insets showing enlarged individual cells are single z sections. Scale bar indicates 5 μm.

(C) Bar graphs illustrate quantification for H2AK119u1 and H3K27me3 domains overlapping Xist in independent cell lines (clones) of each named construct. Error bars represent SD across at least three biological replicates (n > 100).

(D) Example as in (B) for BglG-ΔKI hnRNPK.

(E) RS comparison between two independent BglG-hnRNPK cell lines in windows centered on the region of maximum silencing. The color and area of dots indicate the region size and gene number, respectively. Green and pink gradients represent the range of RS previously measured for FL and XistΔXR-PID lines, respectively.

(F) RS comparison between two independent BglG-hnRNPK cell lines in the 10 Mb of maximum silencing windows. The RS was grouped into four categories, indicated by the graded color.

(G) Model illustrating molecular pathway for Polycomb recruitment by Xist RNA.

DISCUSSION

The results of this study reveal key steps in the pathway for recruitment of Polycomb-repressive complexes by Xist RNA, illustrated in Figure 7G. We suggest that tandemly arranged copies of the cytidine rich B-repeat element recruit multiple hnRNPK subunits. hnRNPK, through its KI domain, interacts directly with the PCGF3/5 subunit of the PCGF3/5-PRC1

complex, which initiates the Polycomb cascade. hnRNPK-bound PCGF3/5-PRC1 may act directly to modify underlying chromatin, or alternatively may dissociate and diffuse to nearby sites. Given the evidence that Xist RNA is anchored to the nuclear matrix (Clemson et al., 1996; Hasegawa et al., 2010), and as a consequence localizes to a compartment that is spatially separated from chromatin (Smeets et al., 2014), we favor the latter proposal. Subsequent steps, involving recognition of PCGF3/5-PRC1-mediated H2AK119u1 by other PRC1 complexes and by PRC2 (Cooper et al., 2016; Almeida et al., 2017), and downstream of this, recognition of H3K27me3 by canonical PRC1 complexes (Fischle et al., 2003; Min et al., 2003), occur as described previously.

Our findings are broadly in accord with prior analysis of Polycomb recruitment by Xist RNA. Thus, the pathway described herein accounts for the close correlation between Polycomb-mediated chromatin modifications and sites of enrichment of Xist RNA, reported in several previous studies (Calabrese et al., 2012; Duthie et al., 1999; Mak et al., 2002; Marks et al., 2009; Pinter et al., 2012). Additionally, the requirement for ongoing Xist expression (Kohlmaier et al., 2004; Mak et al., 2004; Plath et al., 2003) can be attributed to PCGF3/5-PRC1 recruitment through interaction with hnRNPK bound to the B-repeat element of Xist RNA. Although this link was suggested previously, based on identification of hnRNPK and PRC1 proteins among factors that bind Xist RNA either directly or indirectly (Chu et al., 2015), our findings define the key sequence elements and underlying molecular interactions. It should be noted that our experiments were performed using in tissue culture models with Xist transgenes located on autosomes, and we cannot rule out that additional factors make a contribution in the context of X inactivation *in vivo*.

The consensus motif of the Xist B-repeat has a tract of two to four consecutive cytidines, which accords with the preferred binding site for hnRNPK, comprising three separate cytidine patches bound by each of three KH domains (Paziewska et al., 2004). There are 32 copies of the B-repeat motif, which could theoretically allow simultaneous binding of up to ten hnRNPK subunits on a single molecule of Xist RNA. High hnRNPK occupancy may serve to amplify PCGF3/5-PRC1 recruitment to a level sufficient to initiate the Polycomb cascade. This proposal is supported by hnRNPK iCLIP analysis (Cirillo et al., 2016), which defines a strong peak precisely corresponding to the B-repeat. The iCLIP data also indicate a broad region marked by a low-level hnRNPK occupancy, correlating with the location of the C-repeat. Accordingly, we find that deletion of the three copies of the C-repeat present in the XR-PID region marginally reduces Polycomb recruitment in the context of the short EvXist transgenes. It is interesting to note that the 115 nt C-repeat consensus includes two separate 3–5 nt cytidine tracts that could potentially mediate hnRNPK binding, albeit less optimally, given their frequency and spacing. Unlike the B-repeat, the core C-repeat consensus is not amplified in the Xist gene in human and other mammals (Nesterova et al., 2001), indicating that the B-repeat is the key conserved element for Polycomb recruitment by Xist RNA.

Polycomb recruitment in X inactivation has been reported to occur in marsupial mammals (Chaumeil et al., 2011; Mahadevaiah et al., 2009), despite the fact that an independently evolved non-coding RNA, Rsx, functions as the master regulator (Grant et al., 2012). Rsx also comprises several tandemly repeated sequence elements, and it is plausible that one of these has evolved to bind hnRNPK. There may also be other examples of non-coding RNAs, or for that matter coding RNAs, that utilize hnRNPK to concentrate Polycomb-repressive complexes in *cis*. Thus, in future studies it will be important to examine the relationship between hnRNPK RNA-binding sites and Polycomb occupancy across the genome.

In our recent study defining the role of PCGF3/5-PRC1 in the Xist Polycomb recruitment pathway, we reported that chromosome silencing is significantly reduced in the absence of both PRC1 and PRC2 (Almeida et al., 2017). Accordingly, in this study we observed a similar reduction in silencing following deletion of the XR-PID element. We were able to examine this effect in detail by using hybrid mESCs, and by analyzing the EvXist construct with or without XR-PID, targeted in single copy into a homing site on chromosome 11. Chromosome silencing by EvXist was seen to be relatively robust and widespread, albeit reduced relative to FL Xist transgenes. Deletion of XR-PID strongly reduced silencing, an observation that was somewhat surprising, given that the A-repeat, previously defined as the principal element required for Xist-mediated silencing (Wutz et al., 2002), is still present. A possible explanation for this result is that in the absence of functions conferred by Xist elements downstream of the EvXist region, Polycomb recruitment plays a more critical role in supporting A-repeat-mediated silencing. This may involve a role for Polycomb in stabilizing A-repeat-mediated silencing or, alternatively, may relate to a role for Polycomb in compacting the chromosome to facilitate the spread of A-repeat-mediated silencing, supported by our observation that deletion of XR-PID reduces loss of chromatin accessibility associated with Xist expression.

Given that significant chromosome silencing is detected in the absence of XR-PID, presumably due to the presence of the A-repeat and associated factors, it is perhaps surprising that deletion of this element results in such a dramatic loss of the decrease in chromosome accessibility. Indeed, a previous study showed that Xist-dependent loss of chromatin accessibility is abrogated, albeit less dramatically, when the A-repeat is deleted (Giorgetti et al., 2016). Thus, in future studies it will be important to further investigate the interplay of A-repeat and XR-PID pathways, both in Xist-dependent gene silencing and in chromatin accessibility changes.

In conclusion, this study, together with our recent findings, provides key insights into the molecular mechanism for Polycomb recruitment by Xist RNA and moreover highlights a key role for Polycomb in Xist-mediated chromosome silencing. Given the potential importance of lncRNA in guiding chromatin modification, this mechanism provides a model for further studies on lncRNA function both in development and in disease.

STAR★METHODS

Detailed methods are provided in the online version of this paper and include the following:

- KEY RESOURCES TABLE
- CONTACT FOR REAGENT AND RESOURCE SHARING

- EXPERIMENTAL MODEL AND SUBJECT DETAILS
- METHODS DETAILS
  - Plasmids
  - Conventional cloning
  - Ligation independent cloning (LIC)
  - Gibson assembly
  - Bacterial transformation
  - Mouse lines and ESC line derivation
  - Generation of stable cell lines expressing transgenes
  - siRNA knock-down
  - Metaphase spreads
  - Genomic DNA extraction
  - Nuclear extraction and immunoblotting
  - Co-immunoprecipitation (CoIP) assays
  - Expression and purification of PRC1 complexes and hnRNPK
  - PRC1-hnRNPK pull-down assays
  - Immunofluorescence
  - RNA-FISH
  - DNA-FISH on metaphase spreads
  - ImmunoFISH
  - Hyperactive Tn5 production and ATAC-see
  - ATAC-seq sample preparation and sequencing
  - Microscopy
  - 4sU-RNA Immunoprecipitation
  - 4sU-seq library preparation and sequencing
  - Xist *in vitro* transcription
  - RNA purification
  - RNA slot blot
  - Isolation of RNA templates and bound proteins
  - Mass spectrometry
- QUANTIFICATION AND STATISTICAL ANALYSIS
  - Polymorphic sites between 129S1 and CAST mouse genomes
  - 4sU-seq analysis
  - Definition and Calculation of the Repression Score (RS)
  - Microscopy and ATAC-See analysis
  - ATAC-seq data analysis
  - Mass spectrometry analysis
- DATA AND SOFTWARE AVAILABILITY

SUPPLEMENTAL INFORMATION

Supplemental Information includes seven figures and four tables and can be found with this article at https://doi.org/10.1016/j.molcel.2017.11.013.

AUTHOR CONTRIBUTIONS

N.B., T.B.N., G.P., G.W., A. Cerase, A. Castello, S.M., and B.M. conceived and designed experiments. G.P., G.W., C.R., B.A.K., N.S., and T.B.N. conducted experiments; G.W. performed computational analysis; and N.B., G.P., and G.W. prepared and wrote the manuscript.

ACKNOWLEDGMENTS

We would like to thank colleagues in the Brockdorff and Klose labs and Chris Ponting for discussion and comments; Dr. Svenja Hester and the University of Oxford Biochemistry Department Advanced Proteomics Facility for the MS analysis; and the EMBL Gene Core, Dinko Pavlinic, and Vladimir Benes for RNA-seq. Work in the N.B. lab was funded by grants from the European Research Council (340081) and the Wellcome Trust (103768 and 091911). G.P. was funded by a Wellcome Trust PhD studentship (099682), and A. Castello was funded by an MRC Career Development Award (#MR/L019434/1).

Received: June 22, 2017
Revised: September 15, 2017
Accepted: November 10, 2017
Published: December 7, 2017

REFERENCES

Almeida, M., Pintacuda, G., Masui, O., Koseki, Y., Gdula, M., Cerase, A., Brown, D., Mould, A., Innocent, C., Nakayama, M., et al. (2017). PCGF3/5-PRC1 initiates Polycomb recruitment in X chromosome inactivation. Science *356*, 1081–1084.

Anders, S., Pyl, P.T., and Huber, W. (2015). HTSeq—a Python framework to work with high-throughput sequencing data. Bioinformatics *31*, 166–169.

Arnold, P., Schöler, A., Pachkov, M., Balwierz, P.J., Jørgensen, H., Stadler, M.B., van Nimwegen, E., and Schübeler, D. (2013). Modeling of epigenome dynamics identifies transcription factors that mediate Polycomb targeting. Genome Res. *23*, 60–73.

Arrigoni, R., Alam, S.L., Wamstad, J.A., Bardwell, V.J., Sundquist, W.I., and Schreiber-Agus, N. (2006). The Polycomb-associated protein Rybp is a ubiquitin binding protein. FEBS Lett. *580*, 6233–6241.

Beard, C., Hochedlinger, K., Plath, K., Wutz, A., and Jaenisch, R. (2006). Efficient method to generate single-copy transgenic mice by site-specific integration in embryonic stem cells. Genesis *44*, 23–28.

Blackledge, N.P., Farcas, A.M., Kondo, T., King, H.W., McGouran, J.F., Hanssen, L.L., Ito, S., Cooper, S., Kondo, K., Koseki, Y., et al. (2014). Variant PRC1 complex-dependent H2A ubiquitylation drives PRC2 recruitment and polycomb domain formation. Cell *157*, 1445–1459.

Bomsztyk, K., Denisenko, O., and Ostrowski, J. (2004). hnRNP K: one protein multiple processes. BioEssays *26*, 629–638.

Buenrostro, J.D., Giresi, P.G., Zaba, L.C., Chang, H.Y., and Greenleaf, W.J. (2013). Transposition of native chromatin for fast and sensitive epigenomic profiling of open chromatin, DNA-binding proteins and nucleosome position. Nat. Methods *10*, 1213–1218.

Calabrese, J.M., Sun, W., Song, L., Mugford, J.W., Williams, L., Yee, D., Starmer, J., Mieczkowski, P., Crawford, G.E., and Magnuson, T. (2012). Site-specific silencing of regulatory elements as a mechanism of X inactivation. Cell *151*, 951–963.

Cao, R., Wang, L., Wang, H., Xia, L., Erdjument-Bromage, H., Tempst, P., Jones, R.S., and Zhang, Y. (2002). Role of histone H3 lysine 27 methylation in Polycomb-group silencing. Science *298*, 1039–1043.

Cerase, A., Pintacuda, G., Tattermusch, A., and Avner, P. (2015). Xist localization and function: new insights from multiple levels. Genome Biol. *16*, 166.

Chaumeil, J., Waters, P.D., Koina, E., Gilbert, C., Robinson, T.J., and Graves, J.A. (2011). Evolution from XIST-independent to XIST-controlled X-chromosome inactivation: epigenetic modifications in distantly related mammals. PLoS ONE *6*, e19040.

Chen, X., Shen, Y., Draper, W., Buenrostro, J.D., Litzenburger, U., Cho, S.W., Satpathy, A.T., Carter, A.C., Ghosh, R.P., East-Seletsky, A., et al. (2016). ATAC-see reveals the accessible genome by transposase-mediated imaging and sequencing. Nat. Methods *13*, 1013–1020.

Chu, C., Zhang, Q.C., da Rocha, S.T., Flynn, R.A., Bharadwaj, M., Calabrese, J.M., Magnuson, T., Heard, E., and Chang, H.Y. (2015). Systematic discovery of Xist RNA binding proteins. Cell *161*, 404–416.

Cirillo, D., Blanco, M., Armaos, A., Buness, A., Avner, P., Guttman, M., Cerase, A., and Tartaglia, G.G. (2016). Quantitative predictions of protein interactions with long noncoding RNAs. Nat. Methods *14*, 5–6.

Clemson, C.M., McNeil, J.A., Willard, H.F., and Lawrence, J.B. (1996). XIST RNA paints the inactive X chromosome at interphase: evidence for a novel RNA involved in nuclear/chromosome structure. J. Cell Biol. *132*, 259–275.

Cooper, S., Dienstbier, M., Hassan, R., Schermelleh, L., Sharif, J., Blackledge, N.P., De Marco, V., Elderkin, S., Koseki, H., Klose, R., et al. (2014). Targeting polycomb to pericentric heterochromatin in embryonic stem cells reveals a role for H2AK119u1 in PRC2 recruitment. Cell Rep. *7*, 1456–1470.

Cooper, S., Grijzenhout, A., Underwood, E., Ancelin, K., Zhang, T., Nesterova, T.B., Anil-Kirmizitas, B., Bassett, A., Kooistra, S.M., Agger, K., et al. (2016). Jarid2 binds mono-ubiquitylated H2A lysine 119 to mediate crosstalk between Polycomb complexes PRC1 and PRC2. Nat. Commun. *7*, 13661.

da Rocha, S.T., Boeva, V., Escamilla-Del-Arenal, M., Ancelin, K., Granier, C., Matias, N.R., Sanulli, S., Chow, J., Schulz, E., Picard, C., et al. (2014). Jarid2 Is Implicated in the Initial Xist-Induced Targeting of PRC2 to the Inactive X Chromosome. Mol. Cell *53*, 301–316.

de Napoles, M., Mermoud, J.E., Wakao, R., Tang, Y.A., Endoh, M., Appanah, R., Nesterova, T.B., Silva, J., Otte, A.P., Vidal, M., et al. (2004). Polycomb group proteins Ring1A/B link ubiquitylation of histone H2A to heritable gene silencing and X inactivation. Dev. Cell *7*, 663–676.

Dignam, J.D., Lebovitz, R.M., and Roeder, R.G. (1983). Accurate transcription initiation by RNA polymerase II in a soluble extract from isolated mammalian nuclei. Nucleic Acids Res. *11*, 1475–1489.

Dobin, A., Davis, C.A., Schlesinger, F., Drenkow, J., Zaleski, C., Jha, S., Batut, P., Chaisson, M., and Gingeras, T.R. (2013). STAR: ultrafast universal RNA-seq aligner. Bioinformatics *29*, 15–21.

Duthie, S.M., Nesterova, T.B., Formstone, E.J., Keohane, A.M., Turner, B.M., Zakian, S.M., and Brockdorff, N. (1999). Xist RNA exhibits a banded localization on the inactive X chromosome and is excluded from autosomal material in cis. Hum. Mol. Genet. *8*, 195–204.

Eskeland, R., Leeb, M., Grimes, G.R., Kress, C., Boyle, S., Sproul, D., Gilbert, N., Fan, Y., Skoultchi, A.I., Wutz, A., and Bickmore, W.A. (2010). Ring1B compacts chromatin structure and represses gene expression independent of histone ubiquitination. Mol. Cell *38*, 452–464.

Fischle, W., Wang, Y., Jacobs, S.A., Kim, Y., Allis, C.D., and Khorasanizadeh, S. (2003). Molecular basis for the discrimination of repressive methyl-lysine marks in histone H3 by Polycomb and HP1 chromodomains. Genes Dev. *17*, 1870–1881.

Gendrel, A.V., and Heard, E. (2014). Noncoding RNAs and epigenetic mechanisms during X-chromosome inactivation. Annu. Rev. Cell Dev. Biol. *30*, 561–580.

Giorgetti, L., Lajoie, B.R., Carter, A.C., Attia, M., Zhan, Y., Xu, J., Chen, C.J., Kaplan, N., Chang, H.Y., Heard, E., and Dekker, J. (2016). Structural organization of the inactive X chromosome in the mouse. Nature *535*, 575–579.

Grant, J., Mahadevaiah, S.K., Khil, P., Sangrithi, M.N., Royo, H., Duckworth, J., McCarrey, J.R., VandeBerg, J.L., Renfree, M.B., Taylor, W., et al. (2012). Rsx is a metatherian RNA with Xist-like properties in X-chromosome inactivation. Nature *487*, 254–258.

Hasegawa, Y., Brockdorff, N., Kawano, S., Tsutui, K., Tsutui, K., and Nakagawa, S. (2010). The matrix protein hnRNP U is required for chromosomal localization of Xist RNA. Dev. Cell *19*, 469–476.

Inoue, H., Nojima, H., and Okayama, H. (1990). High efficiency transformation of Escherichia coli with plasmids. Gene *96*, 23–28.

Isono, K., Endo, T.A., Ku, M., Yamada, D., Suzuki, R., Sharif, J., Ishikura, T., Toyoda, T., Bernstein, B.E., and Koseki, H. (2013). SAM domain polymerization links subnuclear clustering of PRC1 to gene silencing. Dev. Cell *26*, 565–577.

Kalb, R., Latwiel, S., Baymaz, H.I., Jansen, P.W., Müller, C.W., Vermeulen, M., and Müller, J. (2014). Histone H2A monoubiquitination promotes histone H3 methylation in Polycomb repression. Nat. Struct. Mol. Biol. *21*, 569–571.

Kohlmaier, A., Savarese, F., Lachner, M., Martens, J., Jenuwein, T., and Wutz, A. (2004). A chromosomal memory triggered by Xist regulates histone methylation in X inactivation. PLoS Biol. *2*, E171.

Langmead, B., and Salzberg, S.L. (2012). Fast gapped-read alignment with Bowtie 2. Nat. Methods *9*, 357–359.

Lau, M.S., Schwartz, M.G., Kundu, S., Savol, A.J., Wang, P.I., Marr, S.K., Grau, D.J., Schorderet, P., Sadreyev, R.I., Tabin, C.J., and Kingston, R.E. (2017). Mutation of a nucleosome compaction region disrupts Polycomb-mediated axial patterning. Science *355*, 1081–1084.

Li, H., Handsaker, B., Wysoker, A., Fennell, T., Ruan, J., Homer, N., Marth, G., Abecasis, G., and Durbin, R. (2009). The Sequence Alignment/Map format and SAMtools. Bioinformatics *25*, 2078–2079.

Lyon, M.F. (1961). Gene action in the X-chromosome of the mouse (Mus musculus L.). Nature *190*, 372–373.

Mahadevaiah, S.K., Royo, H., VandeBerg, J.L., McCarrey, J.R., Mackay, S., and Turner, J.M. (2009). Key features of the X inactivation process are conserved between marsupials and eutherians. Curr. Biol. *19*, 1478–1484.

Mak, W., Baxter, J., Silva, J., Newall, A.E., Otte, A.P., and Brockdorff, N. (2002). Mitotically stable association of polycomb group proteins eed and enx1 with the inactive x chromosome in trophoblast stem cells. Curr. Biol. *12*, 1016–1020.

Mak, W., Nesterova, T.B., de Napoles, M., Appanah, R., Yamanaka, S., Otte, A.P., and Brockdorff, N. (2004). Reactivation of the paternal X chromosome in early mouse embryos. Science *303*, 666–669.

Marks, H., Chow, J.C., Denissov, S., Françoijs, K.J., Brockdorff, N., Heard, E., and Stunnenberg, H.G. (2009). High-resolution analysis of epigenetic changes associated with X inactivation. Genome Res. *19*, 1361–1373.

Min, J., Zhang, Y., and Xu, R.M. (2003). Structural basis for specific binding of Polycomb chromodomain to histone H3 methylated at Lys 27. Genes Dev. *17*, 1823–1828.

Moindrot, B., Cerase, A., Coker, H., Masui, O., Grijzenhout, A., Pintacuda, G., Schermelleh, L., Nesterova, T.B., and Brockdorff, N. (2015). A pooled shRNA screen identifies Rbm15, Spen, and Wtap as factors required for Xist RNA-mediated silencing. Cell Rep. *12*, 562–572.

Nesterova, T.B., Slobodyanyuk, S.Y., Elisaphenko, E.A., Shevchenko, A.I., Johnston, C., Pavlova, M.E., Rogozin, I.B., Kolesnikov, N.N., Brockdorff, N., and Zakian, S.M. (2001). Characterization of the genomic Xist locus in rodents reveals conservation of overall gene structure and tandem repeats but rapid evolution of unique sequence. Genome Res. *11*, 833–849.

Patil, D.P., Chen, C.K., Pickering, B.F., Chow, A., Jackson, C., Guttman, M., and Jaffrey, S.R. (2016). m(6)A RNA methylation promotes XIST-mediated transcriptional repression. Nature *537*, 369–373.

Paziewska, A., Wyrwicz, L.S., Bujnicki, J.M., Bomsztyk, K., and Ostrowski, J. (2004). Cooperative binding of the hnRNP K three KH domains to mRNA targets. FEBS Lett. *577*, 134–140.

Pinter, S.F., Sadreyev, R.I., Yildirim, E., Jeon, Y., Ohsumi, T.K., Borowsky, M., and Lee, J.T. (2012). Spreading of X chromosome inactivation via a hierarchy of defined Polycomb stations. Genome Res. *22*, 1864–1876.

Plath, K., Fang, J., Mlynarczyk-Evans, S.K., Cao, R., Worringer, K.A., Wang, H., de la Cruz, C.C., Otte, A.P., Panning, B., and Zhang, Y. (2003). Role of histone H3 lysine 27 methylation in X inactivation. Science *300*, 131–135.

Pullirsch, D., Härtel, R., Kishimoto, H., Leeb, M., Steiner, G., and Wutz, A. (2010). The Trithorax group protein Ash2l and Saf-A are recruited to the inactive X chromosome at the onset of stable X inactivation. Development *137*, 935–943.

Quinlan, A.R., and Hall, I.M. (2010). BEDTools: a flexible suite of utilities for comparing genomic features. Bioinformatics *26*, 841–842.

Rabani, M., Levin, J.Z., Fan, L., Adiconis, X., Raychowdhury, R., Garber, M., Gnirke, A., Nusbaum, C., Hacohen, N., Friedman, N., et al. (2011). Metabolic labeling of RNA uncovers principles of RNA production and degradation dynamics in mammalian cells. Nat. Biotechnol. *29*, 436–442.

Sheardown, S.A., Duthie, S.M., Johnston, C.M., Newall, A.E., Formstone, E.J., Arkell, R.M., Nesterova, T.B., Alghisi, G.C., Rastan, S., and Brockdorff, N. (1997). Stabilization of Xist RNA mediates initiation of X chromosome inactivation. Cell *91*, 99–107.

Silva, J., Mak, W., Zvetkova, I., Appanah, R., Nesterova, T.B., Webster, Z., Peters, A.H., Jenuwein, T., Otte, A.P., and Brockdorff, N. (2003). Establishment of histone h3 methylation on the inactive X chromosome requires transient recruitment of Eed-Enx1 polycomb group complexes. Dev. Cell *4*, 481–495.

Smeets, D., Markaki, Y., Schmid, V.J., Kraus, F., Tattermusch, A., Cerase, A., Sterr, M., Fiedler, S., Demmerle, J., Popken, J., et al. (2014). Three-dimensional super-resolution microscopy of the inactive X chromosome territory reveals a collapse of its active nuclear compartment harboring distinct Xist RNA foci. Epigenetics Chromatin *7*, 8.

Stock, J.K., Giadrossi, S., Casanova, M., Brookes, E., Vidal, M., Koseki, H., Brockdorff, N., Fisher, A.G., and Pombo, A. (2007). Ring1-mediated ubiquitination of H2A restrains poised RNA polymerase II at bivalent genes in mouse ES cells. Nat. Cell Biol. *9*, 1428–1435.

Swanson, M.S., and Dreyfuss, G. (1988). Classification and purification of proteins of heterogeneous nuclear ribonucleoprotein particles by RNA-binding specificities. Mol. Cell. Biol. *8*, 2237–2241.

Tang, Y.A., Huntley, D., Montana, G., Cerase, A., Nesterova, T.B., and Brockdorff, N. (2010). Efficiency of Xist-mediated silencing on autosomes is linked to chromosomal domain organisation. Epigenetics Chromatin *3*, 10.

Wang, J., Mager, J., Chen, Y., Schneider, E., Cross, J.C., Nagy, A., and Magnuson, T. (2001). Imprinted X inactivation maintained by a mouse Polycomb group gene. Nat. Genet. *28*, 371–375.

Wutz, A., Rasmussen, T.P., and Jaenisch, R. (2002). Chromosomal silencing and localization are mediated by different domains of Xist RNA. Nat. Genet. *30*, 167–174.

Zhao, J., Sun, B.K., Erwin, J.A., Song, J.J., and Lee, J.T. (2008). Polycomb proteins targeted by a short repeat RNA to the mouse X chromosome. Science *322*, 750–756.

Zhou, W., Zhu, P., Wang, J., Pascual, G., Ohgi, K.A., Lozach, J., Glass, C.K., and Rosenfeld, M.G. (2008). Histone H2A monoubiquitination represses transcription by inhibiting RNA polymerase II transcriptional elongation. Mol. Cell *29*, 69–80.

STAR★METHODS

KEY RESOURCES TABLE

REAGENT or RESOURCE	SOURCE	IDENTIFIER
Antibodies		
Antibodies for immunofluorescence assays	Commercial	See Table S4
Antibodies for western blotting	Commercial	See Table S4
Antibodies for immunoprecipitation assays	Commercial	See Table S4
Bacterial and Virus Strains		
DH5α Competent Cells	This study	N/A
Biological Samples		
DMEM	Life Technology	cat# 12634-010
OPTI-MEM	Life Technologies	cat# 11058021
Fetal Calf Serum	Seralab	N/A
Chicken Serum	Life Technologies	cat# 16110082
LIF	In house produced	N/A
Chemicals, Peptides, and Recombinant Proteins		
KaryoMAX Colcemid	Life Technologies	cat# 15210-040
Dimethyl sulfate (DMS)	Sigma Aldrich	cat# D186309
4-thiouridine (4sU)	Sigma Aldrich	cat# T4509
Doxycycline	Clonetech	cat# 631311
EZ-Link HPDP-Biotin	Sigma Aldrich	cat# PI21341
Critical Commercial Assays		
Nick translation kit	Abbott Diagnostics	cat# 7J0001
HiScribe T7 High Yield RNA Synthesis Kit	NEB	cat# E2040S
uMacs Streptavidin KiT	Miltenyi	cat# 130-074-101
RNeasy MinElute Cleanup Kit	QIAGEN	cat# 74204
TruSeq Stranded Total RNA Sample Preparation Kit with Ribo-Zero Gold	Illumina	cat# RS-122-2301
Deposited Data		
4sU-seq, ATAC-seq	This study	GEO: GSE103370
4sU-seq, ATAC-seq summary	This study	See Table S2
Mendeley Data, part I	This study	https://doi.org/10.17632/p8835bsb8g.1
Mendeley Data, part II	This study	https://doi.org/10.17632/dfrbvsdcrf.1
Experimental Models: Cell Lines		
Mouse ESC: C57BL/6JJcl x 129/SvJcl (P4D7)	Moindrot et al., 2015	N/A
Mouse ESC: C57BL/6JJcl x 129/SvJcl expressing Xist WT and mut	This study	See Figure S2
Mouse ESC: C57BL/6JJcl x 129/SvJcl expressing Xist-bgl	Moindrot et al., 2015	N/A
Mouse ESC: pgk 12.1	Sheardown et al., 1997	N/A
Oligonucleotides		
Oligonucleotides for conventional and LIC cloning of hnRNPK and Xist cDNA	This study	See Table S3
gRNA for CRISPR-mediated HR	This study	See Table S3
Oligonucleotides for Tn5 assembly	This study; Chen et al., 2016	See Table S3
Recombinant DNA		
pTRE-tight-Xist	Moindrot et al., 2015	N/A
pCAG-IRES-Puro	This study	N/A

(*Continued on next page*)

Continued		
REAGENT or RESOURCE	SOURCE	IDENTIFIER
pSpCas9(BB)-2A-Puro (PX459) V2.0	Addgene	cat# 62988
pCol1a1-EvXist	This study	N/A
Software and Algorithms		
Samtools	Li et al., 2009	http://samtools.sourceforge.net/
Bedtools	Quinlan and Hall, 2010	http://bedtools.readthedocs.io/en/latest/
Bowtie2	Langmead and Salzberg, 2012	http://bowtie-bio.sourceforge.net/bowtie2/index.shtml
SNPsplit	Babraham Institute	http://www.bioinformatics.babraham.ac.uk/projects/SNPsplit/
STAR	Dobin et al., 2013	https://github.com/alexdobin/STAR
Htseq	Anders et al., 2015	http://www-huber.embl.de/HTSeq/doc/overview.html
RS Analysis	This study	https://github.com/guifengwei/XCI

CONTACT FOR REAGENT AND RESOURCE SHARING

Further information and requests for resources and reagents should be directed to and will be fulfilled by the Lead Contact, Neil Brockdorff (neil.brockdorff@bioch.ox.ac.uk).

EXPERIMENTAL MODEL AND SUBJECT DETAILS

ES cells were grown in ES cell medium, which consisted of Dulbecco's Modified Eagle Medium (DMEM, from Life Technologies) supplemented with 9% fetal calf serum (FCS, from Seralab), 2 mM L-glutamine, 1x non-essential amino acids, 50 μM 2-mercaptoethanol, 50 g/mL penicillin/streptomycin (all from Life Technologies) and LIF- conditioned medium, made in house, at a concentration equivalent to 1000U/mL. Non-differentiating ES cells were grown on tissue culture dishes coated with PBS + 0.1% gelatine. EC10 medium consisted of DMEM, supplemented with 9% FCS, 2 mM L-glutamine, 1x non-essential amino acids, 50 μM 2-mercaptoethanol and 50 g/mL penicillin/streptomycin. Cells were grown at 37°C in a humid atmosphere with 5% CO_2. Cells were passaged using 0.05% trypsin-EDTA (Life Technologies) with 2% Chicken Serum (Life Technologies) and frozen in FCS + 10% DMSO.

Xist expression driven by TetOn promoter was induced by adding doxycycline (1.5-2 μg/mL) to the culture medium for 24 hr to 3 days depending on the experiment. To achieve differentiation, ES cells were plated at a low density (0.6·10^6 cells) in 14cm non-gelatineised tissue culture dishes in EC10 medium, and grown in LIF-depleted conditions for 72 hr.

METHODS DETAILS

Plasmids

Xist cDNA was inserted into a pTRE-tight vector as previously described (Moindrot et al., 2015). Deletions of Xist DNA were obtained by digesting the plasmid with relevant restriction enzymes, and, where required, re-inserting restriction fragments derived from the same plasmid. In detail, ΔSX Xist was generated using MluI (cDNA position after TSS: 1bp) and XhoI (cDNA position after TSS position: 1026bp); ΔXN Xist was generated using XhoI (cDNA position after TSS: 1026bp) and NcoI (cDNA position after TSS position: 4882bp); ΔXEv Xist was generated using XbaI (cDNA position after TSS: 2884bp) and EcoRV (cDNA position after TSS position: 3560bp); EvXist was generated truncating Xist after EcoRV (cDNA position after TSS: 3560bp); ΔXR-PID EvXist was generated cutting EvXist with XbaI (cDNA position after TSS: 2684bp) and EcoRV (cDNA position after TSS position: 3560bp); Prox XR-PID EvXist was generated by re-inserting the XbaI fragment (cDNA position after TSS: 2684bp-2884bp) into ΔXR-PID EvXist; Dist XR-PID EvXist was generated by re-inserting the XbaI-EcoRV fragment (cDNA position after TSS: 2884bp-3560bp) into ΔXR-PID EvXist; Inv XR-PID EvXist was generated by re-inserting the XbaI fragment (cDNA position after TSS: 2684bp-2884bp) into ΔXR-PID EvXist, but with inverted orientation; ΔF EvXist was generated by re-ligation of the vector following digestion of EvXist with BlpI (cDNA position after TSS: 1256bp) and AccI (cDNA position after TSS position: 2198bp). For the ΔXEv-Xist-BgI system, 18 repeats of the BgI stem loop motif were fused to Xist, as in (Moindrot et al., 2015).

Coding sequences for hnRNPK (full-length and truncated) were amplified from cDNA obtained from 129S1 wt cell line using primers listed in the Resource Table. These sequences were inserted by LIC cloning into a modified pCAG-IRES-Puro mammalian expression plasmid between the coding sequences for a C- or N-terminal enhanced GFP (eGFP) and a 3′ IRES sequence that precedes a puromycin resistant cassette. For the hnRNPK-BglG fusion, the same coding sequences were inserted by LIC cloning into

the pCAG-IRES-Puro mammalian expression plasmid between the coding sequence for a C-terminal enhanced GFP (eGFP) fused to the BglG protein, and a 3′ IRES sequence that precedes puromycin resistant gene. BglG was PCR-amplified from a previously described vector (Moindrot et al., 2015) using the primers listed in in the Resource Table, and inserted in the pCAG vector by Gibson Assembly (NEB). For *in vitro* transcription studies, functional versus non-functional A-repeats described in (Wutz et al., 2002) were kindly provided by Anton Wutz, cloned into a pBluescript plasmid (Stratagene) and fused to the B-repeat sense and antisense sequence cut from Xist cDNA using restriction sites. In detail, the vector was digested with XhoI, and the B-repeat fragment, excised with XbaI, was blunt-ended and inserted in either orientation. For CRISPR-Cas9-facilitated homologous recombination of Xist into the *Col1a1* locus (Arnold et al., 2013), the targeting vector described in (Beard et al., 2006) was adapted by shortening the homology arms to 0.8 kb on either side, adding MluI/PacI sites in the MCS (primers listed in in the Resource Table) from pTre-tight, and inserting EvXist in the MluI/PacI restriction sites, in the presence or absence of the XR-PID region. Correct targeting was verified by Southern Blot, as in (Beard et al., 2006).

Conventional cloning

Assembly of vectors and inserts was obtained by either sticky-end or blunt-end cloning after gel extraction of purified plasmid-cut DNA using the Zymoclean Gel DNA Recovery Kit (Zymoresearch). Ligation was performed at 16°C for 2 hr, 10°C for 2 hr, and 4°C overnight with T4 DNA Ligase (Promega). In case of non-directional sticky-end cloning, vector DNA was treated with 1U of CIAP (GIBCO) at 37°C for 5 min prior to gel extraction. Blunt-ending was performed by treating DNA with 20U of T4 DNA Polymerase (ThermoScientific) for 30 min at 16°C.

Ligation independent cloning (LIC)

5 μg of vector plasmid was linearized with the appropriate restriction enzyme. The vector DNA was isolated by gel extraction using the Zymoclean Gel DNA Recovery Kit (Zymoresearch). Insert DNA was amplified by PCR and purified by gel extraction using the same kit. Vector and insert were processed by T4 DNA polymerase (ThermoScientific) in the presence of 2 mM dGTP (vector) and 2 mM dCTP (insert) for 30 min at 22°C. After heat-inactivation of the polymerase (20 min, 75°C), samples were purified using the DNA Clean & Concentrator-5 kit (Zymoresearch). Vector and insert were incubated using a range of ratios (3:1-1:6) in 2 μL total volume for 30 min at 25°C and introduced into bacteria as below.

Gibson assembly

Depending on the specific cloning, either two different PCR products, or a PCR product and a linearized vector were isolated by gel extraction using the Zymoclean Gel DNA Recovery Kit (Zymoresearch). 50 ng of donor vector was incubated in a 1:3 ratio with the insert in the presence of 10 μL of Gibson Assembly Master Mix (NEB) in 20 μL total volume at 25°C for 60 min. 5 μL of the reaction were introduced into bacteria.

Bacterial transformation

Competent DH5α (fhuA2 lac(del)U169 phoA glnV44 80’ lacZ(del)M15 gyrA96 recA1 relA1 endA1 thi-1 hsdR17) cells were generated in-house using a protocol based on (Inoue et al., 1990). Bacteria were thawed on ice and 2 μL DNA of ligation mixture was added. DNA was mixed with bacteria by gentle flicking of the tube and incubated on ice for 25-30 min. Bacteria were heat-shocked at 42°C for 30 s and allowed to recover on ice for 2 min, before being incubated in 10 volumes of LB solution at 37°C for 1 hr. Bacteria were then spread on high salt (10 mg NaCl/mL) LB agar plates containing the appropriate antibiotic for selection.

Mouse lines and ESC line derivation

A modified version of pR26/P’nlsrtTA construct in which the puromycin resistance cassette was replaced by a hygromycin resistance cassette, was electroporated into hybrid C57BL/6JJcl x 129/SvJcl cells, to target rtTA into the constitutively active ROSA26 locus as previously described (Tang et al., 2010). Targeting was verified by Southern blot analysis using unique sequence probes. All cell lines carrying an inducible Xist randomly integrated into the genome were generated by co-transfection into rtTA-expressing cells with either a puromycin or neomycin expression cassette, as explained below. Positive clones were verified by RNA-FISH for Xist RNA upon doxycycline treatment. hnRNPK-eGFP fusion cell lines were obtained by transfection of the relevant construct into PGK 12.1 cells, and positive clones verified by western blot. hnRNPK-eGFP-BglG, ΔKI-hnRNPK-eGFP-BglG, and BglG-eGFP cell lines, were obtained by co-transfection into rtTA-expressing cells of either vector in combination with Xist-Bgl, and selected by GFP/Xist ImmunoFISH after doxycycline induction. Single copy EvXist and EvXist ΔXR-PID were obtained by co-transfection of targeting vector expressing a neomycin expression cassette and three different gRNAs (see Resource Table).

Generation of stable cell lines expressing transgenes

All transgenic ES cells in this study are stable expressing lines. To generate stable ES cell lines, cells were plated in 6-well plates on feeders at a density of 1-2$\cdot 10^6$ cells/well, a day before transfection. Cells were transfected using Lipofectamine 2000 (Life Technologies, Invitrogen), according to the manufacturer’s instructions. After 24 hr, transfected cells were passaged to 90 mm gelatinised Petri dishes with feeders. Puromycin (1.5-2.5 μg/mL) or G418 (350-500 μg/mL) selections were applied 48 hr after lipofection. Cells were grown for 10-12 days under selection, with medium being changed every day. Individual ES colonies were picked and

expanded for further screening. For CRISPR-assisted homologous recombination, 1 μg of each gRNA was added to the lipofection cocktail, and antibiotic selection applied 24 hr after transfection for 48 hr.

siRNA knock-down

iGENOME SMARTpools siRNA against hnRNPK were purchased from Dharmacon. Transfection into cells was performed on cover-slips with RNAiMAX (Invitrogen), according to the manufacturer's instructions. Xist was induced at the same time as transfection for the 24 hr time-point, otherwise 24 or 48 hr after transfection.

Metaphase spreads

Cells were grown in T25 flasks until nearly confluent, and incubated at 37°C with fresh medium containing 1.5 μg/mL ethidium bromide (Roche). After 1 hr and 20 min, colchicine (KaryoMAX Colcemid, Life Technologies) was added to a final concentration of 0.1 μg/mL and incubated for a further 40 min. Cells were then washed in PBS and harvested by trypsinisation at room temperature. After inactivation of the trypsin by addition of medium, cells were pelleted by centrifugation (400 g, 3 min, 25°C). A pellet of 1-2 mm thickness was carefully resuspended in 1 mL hypotonic solution (75 mM KCl) for no more than 5 min. Then 200 μL freshly prepared fixative (75% methanol, 25% Acetic Acid, 4°C) was added drop-wise. The tube was not agitated, but carefully placed in the centrifuge for pelleting (400 g, 3 min, 25°C). Supernatant was removed leaving about 100 μL, used to re-suspend the cells in by gentle flicking. 1 mL of fixative was added to the resuspended cells and incubated overnight at 4°C without agitation. The following day, the cells were carefully resuspended in the same fixative and pelleted as before. The pellet was resuspended in 1.5 mL fixative and pelleted again. This step was repeated twice more. The cell suspension was then dropped onto clean microscope slides and air-dried.

Genomic DNA extraction

Cells from a confluent T75 were harvested and resuspended in 1-3 mL lysis buffer (10 mM NaCl, 10 mM Tris-HCl pH 7.5, 10 mM EDTA-NaOH pH 8.0, 0.5% Sodium lauroyl sarcosinate) with proteinase K added to a final concentration of 200 μg/mL. Samples were incubated overnight at 55°C. 1/25 volume of 5 M NaCl and 2.5 volume of ice-cold 100% ethanol were added. After mixing, a visible white cloud of DNA was extracted using a bent pipette tip and transferred to a clean tube containing 1 mL 70% ethanol. DNA was pelleted (16,100 g, 5 min, 4°C) and air-dried. Subsequently the pellet was resuspended in 300-400 μL 10 mM Tris pH 8.5 and the concentration measured by Nanodrop.

Nuclear extraction and immunoblotting

Nuclear cell extracts were prepared by harvesting cells and either processing them immediately or snap-freezing them on dry ice and storing them at −80°C, essentially as described in (Dignam et al., 1983). In both cases, cell pellets were washed with PBS and re-suspended in 10 packed cell volume (PCV) buffer A (10 mM HEPES-KOH pH 7.9, 1.5 mM $MgCl_2$, 10 mM KCl, with 0.5 mM DTT, 0.5 mM PMSF, and complete protease inhibitors (Roche) added fresh). After a 10 min incubation at 4°C, cells were collected by centrifugation (1500 g, 5 min, 4°C) and resuspended in 3 PCV of buffer A + 0.1% NP-40 (Sigma). After another 10 min incubation at 4°C, nuclei were collected by centrifugation (400 g, 5 min, 4°C) and resuspended in 1 PCV buffer C (250 mM NaCl, 5 mM HEPES-KOH (pH 7.9), 26% glycerol, 1.5 mM $MgCl_2$, 0.2mM EDTA-NaOH, pH 8.0 with complete protease inhibitors (Roche) + 0.5 mM DTT added fresh). 5 M NaCl was added drop-wise to bring the concentration to 350 mM and the mixture was incubated for 1 hr at 4°C with occasional agitation. After centrifugation (16,100 g, 20 min, 4°C), the concentration of the supernatant was quantified using the Bio-Rad Bradford assay and stored at −80°C.

Samples for immunoblotting were diluted in 6xSMASH buffer (50 mM Tris HCl pH 6.8, 10% Glycerol, 2% SDS, 0.02% bromophenol blue, 1% β-mercaptoethanol), boiled for 10 min at 95°C, separated on a polyacrylamide gel, and transferred onto a nitrocellulose membrane by semi-dry transfer (15 V for 50 min). Membranes were blocked by incubating them for 1 hr at room temperature in 10 mL TBS, 0.1% Tween (TBST) with 5% w/v Marvel milk powder. Blots were incubated overnight at 4°C with the primary antibody, washed 4 times for 10 min with TBST and incubated for 40 min with secondary antibody conjugated to horseradish peroxidase. After washing 4 times for 5 min with TBST, bands were visualized using ECL (GE Healthcare).

Co-immunoprecipitation (CoIP) assays

In-house GFP nanobodies were prepared by washing 165 μL of M-280 Tosyl-activated Dynabeads (ThermoScientific) twice in buffer A (0.1 M H_2BO_3 pH 9.5), before adding 100 μL of GFP-nanobodies (in-house His-affinity purified from pET21b_pelB_VHH24 expression vector, kind gift of Michael Root, Rockefeller University), for a total volume of 150 μL in buffer A. After addition of 100 μL buffer C (0.1 M H_2BO_3, 3M $(NH_4)_2SO_4$), the mixture was incubated at 37°C overnight. The day after, 1 mL of buffer D (0.01 M BSA in PBS) was added, and beads incubated at 37°C for a further 1 hr. Coupled beads were washed twice in buffer E (0.005 M BSA in PBS), resuspended in buffer E to a final concentration of 20 mg/mL, and stored at 4°C.

For the coIP assays, 500 μg of nuclear extract were used and the salt concentration was adjusted to 150 mM NaCl in a 1 mL total reaction volume. Extracts were treated with 250 U Benzonase nuclease (Millipore) for 30 min at 4°C. 100 μg of nanobodies were added and incubated at 4°C overnight. The flowthrough was collected and beads were washed 3 times with 1 mL wash buffer (5mM HEPES-KOH pH 7.9, 1.5 mM $MgCl_2$, 26% glycerol, 0.2 mM EDTA, with complete protease inhibitors (Roche) + 0.5 mM

DTT added fresh) with 150 mM NaCl, and 3 times with the same wash buffer with 450 mM NaCl. Subsequently beads were boiled in 50 μL SMASH buffer (50 mM Tris HCl pH 6.8, 10% Glycerol, 2% SDS, 0.02% bromophenol blue, 1% β-mercaptoethanol) for 10 min at 95°C. 5 μL of the supernatant was loaded as the IP sample.

Expression and purification of PRC1 complexes and hnRNPK

PRC1.2/ PRC1.6 (RING1B C-term His/Strep, RYBP, PCGF2/6) and PRC1.3 (RING1B, RYBP, PCGF3 C-term His/Flag) complexes were cloned into a psT44 vector, co-expressed in ArcticExpress DE3 bacteria cells (Agilent), and purified by nickel affinity chromatography and gel filtration. For each complex: 2 L of 2xTY media was divided into 3 flasks (700 mL each) and each flask was inoculated with 21 mL overnight pre-culture and 50 μg/mL Ampicillin (700 μL). Once inoculated, flasks were placed in an incubator at 30°C (shaker: 220 rpm) and cells are grown until they reached a 0.8-0.9 OD at 600 nm. Cells were then placed at 12°C for 20 min. Expression was induced by adding 0.5 mM IPTG and cells were then left in the incubator at 12°C for 20-24 hr. After incubation, cells were centrifuged for 15 min at 6,000 rpm at 5°C and pellets re-suspended using 20 mL/L (buffer/culture) lysis buffer (20 mM HEPES pH8, 250mM NaCl, 10% glycerol, 0.1% Triton X-100, 1 tablet of protease inhibitors (1/100 mL), 0.25 mg/mL lysozyme). After re-suspension, cells were placed in a 50 mL falcon tube, flash-frozen in liquid nitrogen and stored at −80°C.

For subsequent extraction of each complex, tubes were placed in a water bath at RT. Once cells were defrosted, 10mM $MgCl_2$, 5 μg DNase1, 1mM Benzamidine were added and each tube was left to shake gently in the cold for 20 min. Cells were then sonicated 8 times at 80%, 30 s on/30 s off, and centrifuged for 1 hr at 20,000 rpm at 4°C. Supernatant was collected and filtrated into a new 50 mL Falcon tube. 10 mM Imidazole and 2 mL of pre-washed Ni Sepharose 6 Fast Flow beads (GE Healthcare) were added into the supernatant and incubated for 2 hr at 4°C. Supernatant was then placed into a glass column. Flowthrough was collected and tube and beads were washed with 10 CV His buffer A (20 mM HEPES pH7.5, 5% glycerol, 500 mM NaCl, 2mM fresh 2-Mercaptoethanol), 2%–6% His Buffer B (20 mM HEPES pH7.5, 5% glycerol, 500 mM NaCl, 2mM fresh β-mercaptoethanol, 500mM Imidazole), and eluted with 2CV in 10%–60% His Buffer B. Collected fractions were loaded on a 12% Acrylamide SDS-PAGE gel and those containing the complex were pooled together, concentrated to 500 μL using a Sartorius Vivaspin 20 50K Centrifugal Concentrator and run on a gel filtration Superdex 200 Increase 10/300 GL column (GE Healthcare) using an Äktapurifier system (GE Healthcare) (Gel filtration buffer: 20 mM HEPES pH7.5, 5% glycerol, 300 mM NaCl, 2 mM fresh β-mercaptoethanol).

GST-hnRNPK was cloned into a pGEX-KT vector, and similarly expressed and extracted. The protein was then isolated using a GST-Trap HP column (GE Healthcare) on an Äktastart system (GE Haelthcare). In detail, 5 μg/mL DNaseI, 10 mM $MgCl_2$, 1 mM benzamidine were added to the cell culture, with shaking at 4°C for 20 min. Cells were then lysed with a French Press at 30 kpsi, and centrifuged at 16,000 rpm for 1 hr at 4°C. The cell lysate was loaded onto the column using a peristatic pump, the column then connected to an Äktastart system (GE Healthcare), was washed with 10 CV Buffer A (20 mM HEPES pH7.5, 5% glycerol, 300 mM NaCl, 500 mM Glucose, 2 mM fresh β-mercaptoethanol, 1 mM EDTA; 0.1% Triton) and eluted with 7 CV Buffer B (20 mM HEPES pH7.5, 5% glycerol, 300 mM NaCl, 500 mM Glucose, 2 mM fresh β-mercaptoethanol; 1 mM EDTA, 0.1% Triton, 10 mM reduced glutathione).

Further purification by Ion Exchange was achieved using a 1 mL HiTrap Q HP column (GE Healthcare). In detail, the sample was diluted to 100 mM NaCl and loaded onto the column, subsequently washed with 5 CV 10% Buffer B, eluted with 10 CV 10 to 100% Buffer B and washed again with 5 CV 100% Buffer B. Final concentration and gel filtration step of the sample were performed as above.

PRC1-hnRNPK pull-down assays

PRC1 complexes sample and hnRNPK sample were mixed together to a final concentration of 30 μg of each protein and incubated overnight at 4°C with gentle shaking. The next day 10 μL of pre-washed Glutathione Sepharos HP beads (GE Helathcare) were added to each samples in pull-down buffer (20 mM HEPES, 300 mM NaCl, 5% Glycerol, 0.1% Triton X-100, 250 mM Sucrose, 2 mM β-mercaptoethanol), and incubated for 2 hr at 4°C on a wheel. Beads were spun 2 min at 2,000 rpm at 4°C, and the supernatant (FT) collected. Two wash steps were performed by adding 100 μL of pull-down buffer and spinning for 2 min at 2,000 rpm at 4°C. Supernatant was collected (W1 and W2). The elution was performed by adding 20 μL elution buffer (20 mM HEPES, 300 mM NaCl, 5% Glycerol, 0.1% Triton X-100, 250 mM Sucrose, 2 mM β-mercaptoethanol, 10 mM reduced glutathione), incubating the samples in the same conditions as above for 15-30 min with gentle mixing. Supernatant (E) was collected after spinning for 2 min at 3,000 rpm at 4°C, and a further 20 μL of elution buffer was added, spun for 2 min at 3,000 rpm at 4°C and supernatant collected and added with the previous elution fraction. Beads (B) were also collected by adding 40 μL of pull-down buffer. All collected fractions were diluted in 6xSmash buffer (50 mM Tris.HCl pH 6.8, 10% Glycerol, 2% SDS, 0.02% bromophenol blue, 1% β-mercaptoethanol), incubated for 5min at 95°C and kept at −20°C until western blot assays were performed.

Immunofluorescence

Cells were plated on slides or coverslips (13 mm diameter coverslips from VWR), at least a day before the experiment. On the day of the experiment, cells on slides were washed with PBS and then fixed with 2% formaldehyde for 15 min, followed by 5 min of permeabilisation in 0.4% Triton X-100. Cells were briefly washed with PBS before blocking with a 0.2% w/v PBS-based solution of fish gelatine (Sigma) for three periods of 10 min. Primary antibody dilutions were prepared in fish gelatine solution with 5% normal goat or

normal donkey serum depending on the secondary antibody used. Primary antibody dilutions are listed in the Resource Table. Cells on slides were incubated with primary antibodies for 1.5 hr in a humid chamber at room temperature. Slides were washed three times in fish gelatine solution. Secondary antibodies were diluted in fish gelatine solution and incubated with cells on slides for 45 min in a humid chamber at 37°C. After incubation, slides were washed twice with fish gelatine and one time with PBS before mounting using Vectashield mounting medium with 4,6-diamidino-2-phenylindole (DAPI). Excess mounting medium was removed and the coverslips were sealed to slides using nail varnish.

RNA-FISH

Cells were plated on either slides or coverslips. After washing twice with PBS, cells were fixed for 10 min with 2.6% formaldehyde followed by permeabilisation with 0.4% Triton X-100 for 5 min at 4°C. After a quick PBS wash, cells were incubated with probes in a humid chamber overnight at 37°C. Xist RNA probes were generated from an 18 kb fragment spanning the whole Xist transcript using a nick translation kit (Abbott Molecular) as previously described (Moindrot et al., 2015). Labeled RNA probes (1.5 μL) were co-precipitated with 10 μg salmon sperm DNA, 1/10 volume 3 M sodium acetate (pH 5.2) and 3 vol ethanol. After washing in 75% ethanol, the pellet was dried, resuspended in 6 μL formamide and denatured at 75°C for 7 min before flash cooling on ice. Probes were diluted in 6 μL 2x hybridization buffer (5x SSC, 12.5% dextran sulfate, 2.5 mg/mL BSA (NEB)), added to the slide/coverslips and incubated overnight at 37°C in a humid chamber. After incubation, slides/coverslips were washed three times with a solution of 2xSSC/50% formamide followed by three washes with 2xSSC in a water bath at 42°C. Slides/coverslips were mounted and sealed as for immunofluorescence.

DNA-FISH on metaphase spreads

Metaphase spreads prepared on slides were de-hydrated in 75%, 80%, and 100% ethanol for 2 min at RT, denatured in 50% formamide/2xSSC at 65°C for 5 min, and de-hydrated again in ice-cold 75% ethanol for 2 min, followed by another 2 min in 80%, and 100% at 25°C. Xist probes were prepared as for RNA-FISH. Once the probes were added to the slides, another 3 min of denaturation at 65°C were performed before over-night incubation at 37°C in a humid chamber. After incubation, slides were washed three times with a solution of 2xSSC/50% formamide followed by three washes with 2xSSC in a water bath at 42°C. Slides were mounted and sealed as for immunofluorescence.

ImmunoFISH

RNA FISH was performed essentially as previously described. Cells grown on slides or coverslips were washed in PBS, and fixed with 4% formaldehyde for 10 min before permeabilisation in 0.4% Triton X- 100 for 5 min. After washing in PBS, slides were incubated with 50% formamide in 2xSSC for 2 hr. Washes were performed twice in 50% formamide in 2x SSC for 3 min at 42°C, twice in 2xSSC for 3 min at 42°C and twice in PBS at room temperature. Slides were subsequently blocked in 0.2% fish gelatine (Sigma) three times for 10 min each, and primary antibodies were added in 0.2% fish gelatine and 5% normal goat serum in PBS. The slides were then processed in an identical manner to the immunofluorescence protocol detailed above.

Hyperactive Tn5 production and ATAC-see

Hyperactive Tn5 protein production was achieved as previously described (Chen et al., 2016). The fluorophore-conjugated adaptors (Life Technology) carry an Alexa-594 fluorophore (Resource Table). The assembly of Tn5 transposome was obtained with the following components: 0.1 vol annealed oligos (50 μM each double-stranded), 0.12 vol 2x Tn5 Dialysis buffer containing DTT, 0.34 vol Glycerol (100%), 0.1 vol purified Tn5 transposases (20 μM in stock) and 0.34 vol Nuclease-Free water. The solution was left on the bench at room temperature for 1 hr. Cells for ATAC-see were seeded on coverslips, and induced with doxycycline for 24 hr. Subsequently, a modified protocol for Xist RNA-FISH (described above) was employed, where immediately after permeabilisation, coverslips were washed once in PBS and incubated at 37°C for 1 hr with a transposase mixture solution (10 μL 5 × TD buffer, final concentration of 100 nM Tn5-Alexa, adding dH2O up to 50 μL). After the transposase reaction, slides were washed with PBS three times, and RNA-FISH continued as above.

ATAC-seq sample preparation and sequencing

Chromatin accessibility was assayed using an adapted version of the Assay for Transposase Accessible Chromatin- (ATAC)-seq. Briefly, 10 million cells treated with doxycycline and the same number of matching untreated controls were harvested, washed with PBS and nuclei were isolated in 1 mL HS Lysis buffer (50 mM KCl, 10 mM $MgSO_4 \cdot 7H_2O$, 5 mM HEPES, 0.05% NP40, 1 mM PMSF, 3 mM DTT) containing protease inhibitors (Roche) for 1 min at room temperature and on ice for further 3 min. Nuclei were centrifuged at 1000 g for 5 min at 4°C, and washed three times with ice-cold RSB buffer (10 mM NaCl, 10 mM Tris (pH 7.4), 3 mM $MgCl_2$). 50,000 nuclei were resuspended in Tn5 reaction buffer (10 mM TAPS, 5 mM MgCl2, 10% dimethylformamide), 2.5 μL of home-made Tn5 transposase (20 μM in stock), and incubated at 37°C for 30 min. The tagmented DNA was purified with ChIP DNA Clean & Concentrator. ATAC-seq libraries were prepared by PCR amplification (12-13 cycles), using custom Illumina barcodes as previously described (Buenrostro et al., 2013) and NEBNext High-Fidelity 2X PCR Master Mix. Libraries were purified with

three rounds of Agencourt AMPure XP bead cleanup (0.5X, 1.1X, 1.1X) to remove out-of-size fragments, and quantified by qPCR using KAPA Library Quantification DNA standards. ATAC-seq libraries were sequenced on Illumina NextSeq500 using 80 bp paired-end reads in biological duplicates.

Microscopy

Z stack images were acquired with a DeltaVision system (Applied Precision) with a 1003/1.40 NA objective (Olympus) and deconvolved using the SoftWorx software algorithm (Applied Precision, conservative ratio method, 10 iterations). Best exposure time for each field and channel was manually determined and used for all subsequent experiments. Further image editing and refinement was achieved through Fiji/ImageJ.

4sU-RNA Immunoprecipitation

4sU-RNA was generated and isolated essentially as described in (Rabani et al., 2011). In detail, 4-thiouridine (4sU, Sigma, T4509) was dissolved in sterile PBS and stored at −20°C. 4sU was thawed just before use and added to the cells in the growing media at a concentration of 500 μM. Cells were incubated with the 4sU-supplemented medium for 12 min. Cell culture medium was rapidly removed from cells and 5 mL of Trizol reagent (Life Technologies) was added. Total RNA was extracted as described above, treated with DNase using the Ambion DNA-free DNase Treatment kit (Life Technologies) according to the manufacturer's instructions, and resuspended in water. For each μg of total RNA, 2 μL of Biotin-HPDP (Pierce, 50mg EZ-Link Biotin-HPDP), previously dissolved in DMF at a concentration of 1 mg/mL, and 1 μL of 10xBiotinylation buffer (100 mM Tris HCl pH 7.4, 10 mM EDTA), was added. The reaction was incubated with rotation for 15 min at 25°C. RNA was transferred to Phase Lock Gel Heavy Tubes (Eppendorf), and an equal volume of chloroform was added. After vigorously mixing, tubes were left incubating for 3 min at 25°C and then centrifuged at 13,000rpm for 5 min at 4°C. The upper phase was transferred to new Phase Lock Gel Heavy Tubes, and chloroform added again. After further centrifugation, the upper phase was transferred to a tube containing an equal volume of isopropanol and 1/10 volume of 5 M NaCl. After inversion, the tubes were centrifuged at 13,000 rpm for 20 min at 4°C. Pellet was washed in 75% ethanol and resuspended in water.

Biotinylated 4sU-RNA was recovered using the μMacs Streptavidin Kit (Miltenyi), with a modified protocol. Per μg of recovered biotynilated 4sU-RNA, 0.5 μL of streptavidin beads were added, in a total volume of 200 μL. Samples were incubated with rotation for 15 min at 25°C. μMacs columns supplied with the μMacs Streptavidin Kit were equilibrated in 1 mL of washing buffer (100 mM Tris HCl pH 7.5, 10 mM EDTA, 1 M NaCl, 0.1% Tween 20) at 65°C. Samples were added to the columns that were then washed 6 times with washing buffer, 3 times at 65°C and three times at 25°C. RNA was eluted in freshly-prepared 100 mM DTT. RNA was further purified using the RNA Mini Clean & Concentrator kit (Zymoresearch) according to the manufacturer's guidelines. 1 μL of 4sU-labeled RNA was quality-checked using the Agilent RNA 6000 Pico kit (Agilent technologies) according to the manufacturer's instructions, and run on a 2100 Bioanalyzer Instrument (Agilent).

4sU-seq library preparation and sequencing

Libraries for RNA-seq were constructed using TruSeq Stranded Total RNA Library Prep Kit (Illumina), with the incorporation of dUTP in the second strand synthesis, and sequenced at the GeneCore facility (EMBL, Heidelberg) using either Illumina HiSeq2000 or Illumina NextSeq500 sequencers.

Xist *in vitro* transcription

RNAs were transcribed from a T7 promoter, following linearization with excess of PvuI-HF (NEB). 1 μg of DNA was in vitro transcribed using the HiScribe T7 High Yield RNA Synthesis Kit (NEB), according to the manufacturer's guidelines. In the reaction 1 μL of Biotin-16-dUTP (Sigma) was added, at a concentration of 50 μM. The size of the RNA was checked on a 1% agarose gel electrophoresed in MOPS buffer (0.2 M MOPS pH 7.0, 0.05 M Na acetate, 0.005 M EDTA pH 8.0). Before loading on the gel, 0.5 μg of each RNA sample, were diluted into 5xMOPS buffer, in the presence of 20 μL of formamide and 7 μL of 37% formaldehyde, and heated at 68°C for 15 min.

RNA purification

In vitro transcribed RNA was diluted in a final volume of 100 μL, treated with DNase using the Ambion DNA-free DNase Treatment kit (Life Technologies) according to the manufacturer's instructions, and resuspended in water. DNA-free RNA was subsequently added to Phase Lock Gel Heavy Tubes (Eppendorf), with an equal volume of chloroform. After vigorously mixing, tubes were left incubating for 3 min at 25°C and then centrifuged at 13,000 rpm for 5 min at 4°C. The upper phase was transferred to normal tubes containing an equal volume of isopropanol and 1/10 volume of 5 M NaCl. After inversion, the tubes were centrifuged at 13,000 rpm for 20 min at 4°C. Supernatant was washed in 75% ethanol and resuspended in water.

RNA slot blot

500 ng of each RNA sample were diluted into 10 μL, and mixed with 20 μL of formamide, 7 μL of 37% formaldehyde, 2 μL of 20xSSC. Tubes were heated at 68°C for 15 min, chilled on ice and diluted in 2 volumes of 20xSCC. Samples were then loaded on a blotter, and transferred onto an Amersham Hybond-XL nylon membrane (GE Healthcare). RNA was then UV cross-linked to the membrane using the optimal crosslink setting on a Stratalinker (120,000 μJ/cm2). The membrane was blocked by incubation for 1 hr at room temper-

ature in 10 mL TBS, 0.1% Tween (TBST) with 5% w/v Marvel milk powder, and incubated for 1 hr at 25°C with Neutravidin-HRP antibody, washed 4 times for 10 min with TBST and visualized using ECL (GE Healthcare).

Isolation of RNA templates and bound proteins

Nuclear extracts (15 μg) from PGK12.1 cells were incubated with 10 μg of each RNA in the presence of 5 μL RNasin Ribonuclease Inhibitors overnight at 4°C in 1 mL total volume of incubation buffer (150 mM NaCl, 15 mM Tris HCl (pH 7.5), 0.5 mM EDTA-NaOH, pH 8.0, 0.025% NP-40, with complete protease inhibitors (Roche) + 1 mM DTT added fresh). Samples were transferred to a tissue culture 6-well plate on ice, and UV cross-linked using a Stratalinler at 250,000 mJ/cm2. Samples were transferred back to protein LoBind micro-centrifuge tubes (Eppendorf), and 50 μL of M-280 Streptavidin Dynabeads (ThermoFisher) were added to each of them. Tubes were incubated with rotation at 4°C overnight. Flowthrough was collected for further analysis, whereas beads were washed 2 times in wash buffer A (150 mM NaCl, 15 mM Tris HCl (pH 7.5), 0.5 mM EDTA-NaOH, pH 8.0 with complete protease inhibitors (Roche) + 1 mM DTT added fresh), 2 times in wash buffer B (450 mM NaCl, 15 mM Tris HCl pH 7.5, 0.5 mM EDTA-NaOH, pH 8.0, 0.025% NP-40, 0.05% SDS, with complete protease inhibitors (Roche) + 0.1 mM DTT added fresh), and 2 times in wash buffer C (150 mM NaCl, 15 mM Tris HCl pH 7.5, 0.5 mM EDTA-NaOH, pH 8.0, with complete protease inhibitors (Roche)). Elution was performed in elution buffer (15 mM Tris HCl pH 7.5, 0.02% SDS) for 15 min at 65°C.

Mass spectrometry

Peptides were resuspended in 5% formic acid and 5% DMSO and then trapped on a C18 PepMap100 pre-column (300 μm i.d. x 5 mm, 100 Å, Thermo Fisher Scientific) using 0.1% formic acid in water at a pressure of 500 bar and analyzed on an Ultimate 3000 UHPLC system (Thermo Fischer Scientific) coupled to a QExactive mass spectrometer (Thermo Fischer Scientific). The peptides were separated on an in-house packed analytical column (50 cm x 75 μm i.d. packed with ReproSil-Pur 120 C18-AQ, 1.9 μm, 120 Å) and then electrosprayed directly into an QExactive mass spectrometer (Thermo Fischer Scientific) through an EASY-Spray nano-electrospray ion source (Thermo Fischer Scientific) using a linear gradient (length: 60 min, 7% to 28% solvent B (0.1% Formic acid in acetonitrile), flow rate: 200 nL/minute). The raw data was acquired on the mass spectrometer in a data-dependent mode (DDA). Full scan MS spectra were acquired in the Orbitrap (scan range 350-2000 m/z, resolution 70000, AGC target 3xe6, maximum injection time 100 ms). After the MS scans, the 20 most intense peaks were selected for HCD fragmentation at 30% of normalized collision energy. HCD spectra were also acquired in the Orbitrap (resolution 17500, AGC target $5xe^4$, maximum injection time 120 ms) with first fixed mass at 180 m/z. The raw data files generated were processed using MaxQuant (Version 1.5.0.35), integrated with the Andromeda search engine as previously described. To identify protein groups, peak lists were searched against mouse database as well as list of common contaminants by Andromeda. Trypsin with a maximum number of missed cleavages of 2 was chosen. Acetylation (Protein N-term, i.e., only the n-terminus of the protein), Oxidation (M) and Phosphorylation (S, T and Y) were used as variable modifications while Carbamidomethylation (C) was set as a fixed modification. Protein and PTM false discovery rate (FDR) were set at 0.01 and a minimum score of 40 and localization probability of 0.7 for phospho-peptides. Match between runs was applied.

QUANTIFICATION AND STATISTICAL ANALYSIS

Polymorphic sites between 129S1 and CAST mouse genomes

Polymorphic sites (only SNPs, not indels) for 129S1 and Cast mouse strains were annotated based on the Sanger Mouse Genome Project, using the GRC38/mm10 genome assembly (dbSNP142). 5,878,742 and 22,603,978 high quality SNPs were reported for 129S1 and Cast genomes, respectively. The polymorphic sites between 129S1 and Cast were directly compared, resulting in a total of 23,005,850 polymorphic sites between the genomes. By comparing each genome with the C57BL/6J reference genome, 25,726,959 polymorphic sites were found to be present in both 129S1 and Cast genome sequences, and were therefore employed to generate an 'N-masked' genome sequence. Although only 41.17% (9471317) SNPs are annotated in genic regions (the mouse genome annotation was downloaded from GENOCDE_vM8), 96.74% of annotated transcripts contain at least one SNP site. Among the genic SNPs, 8,803,708 (92.95%) SNPs are located in intronic regions.

4sU-seq analysis

The raw fastq files of read pairs were first mapped to rRNA build, then the remaining unmapped reads were aligned to the 'N-masked' genome with STAR using the parameters '–outFilterMultimapNmax 1 –outFilterMismatchNmax 2 –alignEndsType EndToEnd' for all the 4sU-seq libraries (Dobin et al., 2013). Unique alignments were retained for further analysis. We employed the SNPsplit to separate the alignment into distinct alleles (Cast and 129S1) with the paired mode. The summary of the allelic split is listed in Table S2. To call the allelic gene expression, the number of unique mapped read pairs for each annotated gene (CPM) was counted using the HT-seq (Anders et al., 2015) with the parameters "-t transcript –s reverse," then normalizing to million mapped read pairs (library size) with edgeR R package, and then splitting the CPM value by the G1 (Cast.) or G2 (129S1) ratio. Cast Allelic expression is CPM * G1/(G1+G2); 129S1 allelic expression is CPM * G2/(G1+G2). Here G1 or G2 is the number of allele-specific read pairs. The genes whose expression level was on average above one in each sample were kept for further analysis (for libraries of time-course 4sU-seq of full-length Xist RNA, only the genes whose expression value sum over the complete time course from Cast and 129S1 alleles was more than 8 were kept; while for the experiments of various truncated Xist RNA, the threshold was set to 6). For visualization of RNA-seq

data in UCSC genome browser, the coverage was generated by Bedtools (v2.17.0) (Quinlan and Hall, 2010) and was normalized to 10 million mapped reads.

Definition and Calculation of the Repression Score (RS)

Based on the assumption that transcriptional variation affects the alleles equally, the Repression Score (RS) of each gene was defined as:

$$RS = \frac{Yi - Y0}{Y0} - \frac{Xi - X0}{X0}$$

Where, 'X' and 'Y' represent the value of gene expression for 129S1 or Cast alleles, respectively. '0' represents the untreated condition (no dox) of each experiment, while 'i' represents the treated condition (72 hr treatment or time-course treatment of 24, 48, or 72 h). Given that the repression score (RS) for the majority of genes is between 0 and 1, all repression scores larger than 1 were set to 1, while all repression scores lower than 0 were set to 0. In regards to Cast versus 129S1, we iterated our genome wide analysis of RS assuming that the integration event occurred in either the Cast or 129 genome, and then selected the best fit. The chromosome in which the largest number of genes showed significant allele-specific repression was defined as being subject to Xist-mediated silencing. RS for the silent alleles were subsequently compared pairwise to all other chromosomes.

For measuring silencing efficiency across the chromosome, sequential 10 Mb bin windows were used. The distribution of the RS was therefore compared between windows across the maximum-silenced chromosome. The maximum silencing region within the chromosome was determined as the region containing maximum repression score. The analysis was further refined by narrowing the windows to 5 Mb and 3 Mb windows. In order to minimize the gene expression variabilities in response to dox treatment, a calibrated Repression Score (cRS) was also defined. First, random Repression Score (rRS) was defined for the candidate allele either in independent control cell lines (used for precise silencing comparison for EvXist), or in all the other alleles of the analyzed cell line. cRS was calculated by subtraction of rRS from the RS: cRS = RS - rRS.

To evaluate the statistical significance of the RS for different cell lines harboring Xist transgenes, a p value based on a permutation method was designed and applied to each gene. Reads from the corresponding counts files were randomly sampled (NoDox and Dox treatment) and assigned to the Cast or 129S1 alleles on the basis of a binomial distribution. Repression Score (RS_{null}) was calculated based on the permutated reads counts, and permutated N (N = 10000) times. The p value was defined as the fraction of RS_{null} which was no less than RS_{obs} (*i.e.* sum(ifelse($RS_{null} >= RS_{obs}$,1,0))/N, N = 10000). The permutation scheme was applied chromosome-wide. The p value for two biological replicates was calculated separately and then combined based on Fisher's method. q-values represent combined p values after adjustment following the Benjamini-Hochberg multiple testing in R. Definition of a "silent gene" is based on both the calibrated RS and q-value ($q < 0.05$).

All the custom scripts, and pipeline used in this study are available at https://github.com/guifengwei/XCI. PCA analysis was based on the repression score of genes on the silenced chromosomes (e.g., Chr11 for EvXist comparison) with FactoMineR R package (v1.35). All the heatmaps generated in this study were plotted using pheatmap R packages, and bubble plots using ggplot2.

Microscopy and ATAC-See analysis

All images were edited and refined through Fiji/ImageJ, number of analyzed cells and biological replicates depend on the experiment are always referred to in the matching figure legend. For ATAC-see analysis, a minimum of 20 cells were analyzed in triplicate as follows: a ROI was manually designed around the Xist domain in the stack where its intensity was the most prominent. ATAC signal was subsequently averaged for 6 stacks around the selected stack, and intensity compared between the Xist ROI and the entire nucleus (segmented based on DAPI signal). Significance was calculated using a paired two tailed Student's t test.

ATAC-seq data analysis

The raw paired-end reads were mapped to 'N_masked' mm10 genome with STAR (2.4.2a) (Dobin et al., 2013) using the same parameters used for 4sU-seq analysis except "–alignIntronMax 10." The unique alignments were kept for the further analysis. SNPsplit was employed to assign the reads allele-specifically. The bedGraph files containing the library-size (10 million mapped reads) normalized differences between G1 (Cast.) and G2 (129S1) for each biological replicate were generated by Bedtools (Quinlan and Hall, 2010). The differences between Dox and NoDox treatment were defined as follows:

$$ddscore = [G1(Dox) - G2(Dox)] - [G1(NoDox) - G2(NoDox)]$$

The ddscore was stored in bedGraph format. For the functional *cis*-elements across the chromosomes, the pre-defined ChromHMM state (https://github.com/guifengwei/ChromHMM_mESC_mm10) across chr2 and chr3 was used. The average ddscores were calculated to represent the state across the corresponding chromosome.

Mass spectrometry analysis

For the Xist A+B+ interactome, all hits annotated as contaminants by the Andromeda research engine were rejected. Subsequently all identified hits were compared with those identified as Xist A-B- interactors. The reported protein intensity was subtracted between the lists, and only the hits showing a > 1 difference, were further analyzed as potential A- or B-repeat binders by comparing their

intensity to that measured in the Xist A-B+, and Xist A+B- experiments, respectively. Data before and after filtering can be found in Table S1.

DATA AND SOFTWARE AVAILABILITY

Original unprocessed gel images in this manuscript have been deposited to Mendeley Data and are available following these links: https://doi.org/10.17632/p8835bsb8g.1, https://doi.org/10.17632/dfrbvsdcrf.1.

The high-throughput data reported in this study have been deposited in GEO under accession number GSE103370.

Molecular Cell, Volume *68*

Supplemental Information

hnRNPK Recruits PCGF3/5-PRC1

to the Xist RNA B-Repeat to Establish

Polycomb-Mediated Chromosomal Silencing

Greta Pintacuda, Guifeng Wei, Chloë Roustan, Burcu Anil Kirmizitas, Nicolae Solcan, Andrea Cerase, Alfredo Castello, Shabaz Mohammed, Benoît Moindrot, Tatyana B. Nesterova, and Neil Brockdorff

Supplemental Figures

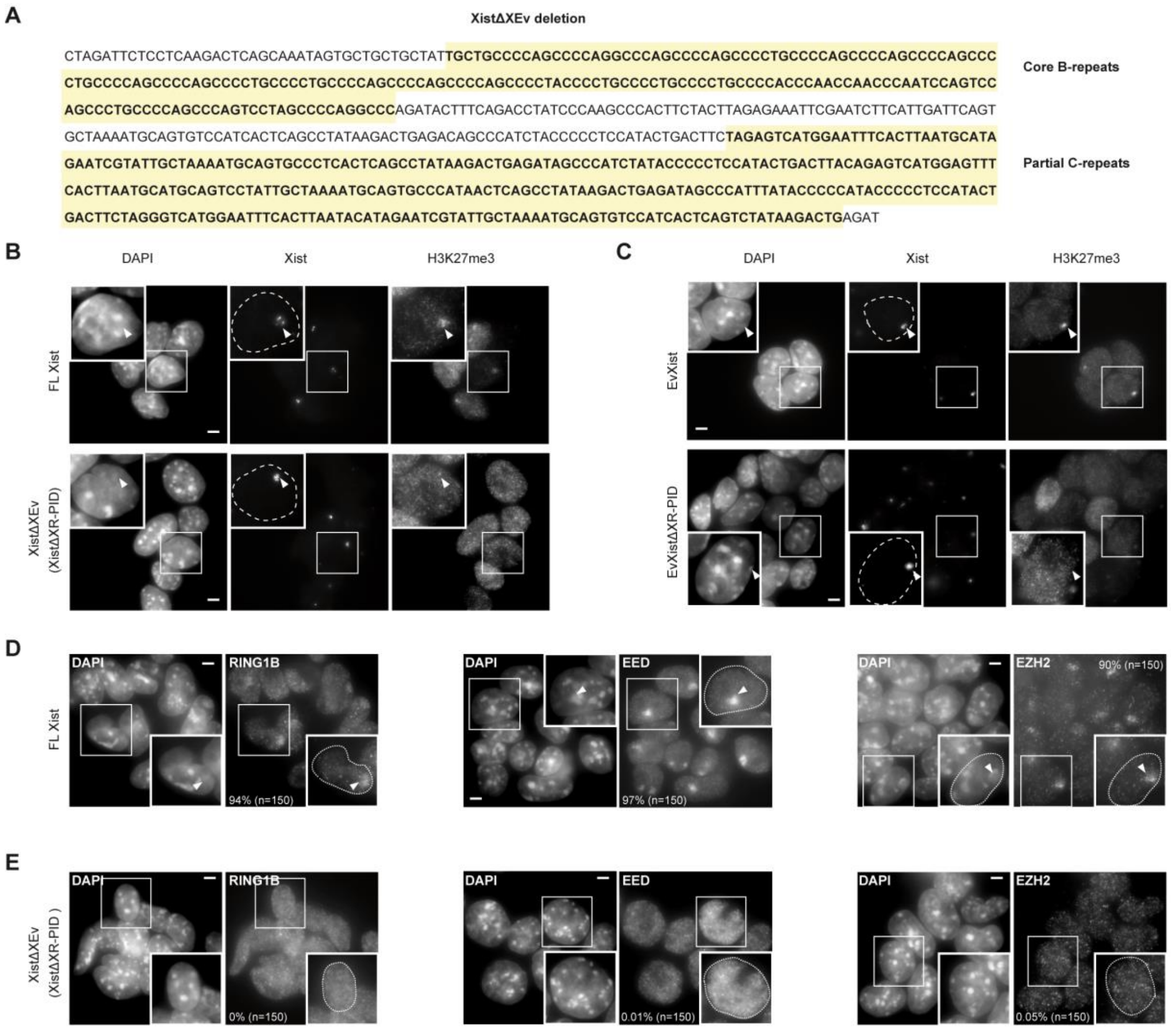

Figure S1. Related to Figure 1. Definition of Xist RNA elements required for Polycomb recruitment.

(A) The Xist sequence deleted in the XistΔXR-PID transgene is shown. The B-repeat and the partial C-repeat sequences are highlighted in yellow.

(B and C) Examples illustrating ImmunoFISH detection of Xist RNA and H3K27me3 after inducing cells with doxycycline for 24 h using the Xist transgene constructs indicated. Wide-field images represent average of 6 consecutive z-sections, cells in boxes were enlarged and represented as single z-sections. Arrows indicate Xist domains. DNA was counterstained with DAPI. Dotted lines in insets indicate outline of DAPI stained nuclei. Scale bar is 5 μm.

(D and E) Examples illustrating immunofluorescence detection of RING1B, EED, and EZH2 after inducing cells with doxycycline for 24 h using the Xist transgene constructs indicated. Scoring was for the number of cells with single nuclear domains. Wide-field images represent the average of 6 consecutive z-sections, cells in boxes were enlarged and represented as single z-sections. Arrows indicate domains of enrichment. DNA was counterstained with DAPI. Dotted lines in insets indicate outline of DAPI stained nuclei. Scale bar is 5 μm.

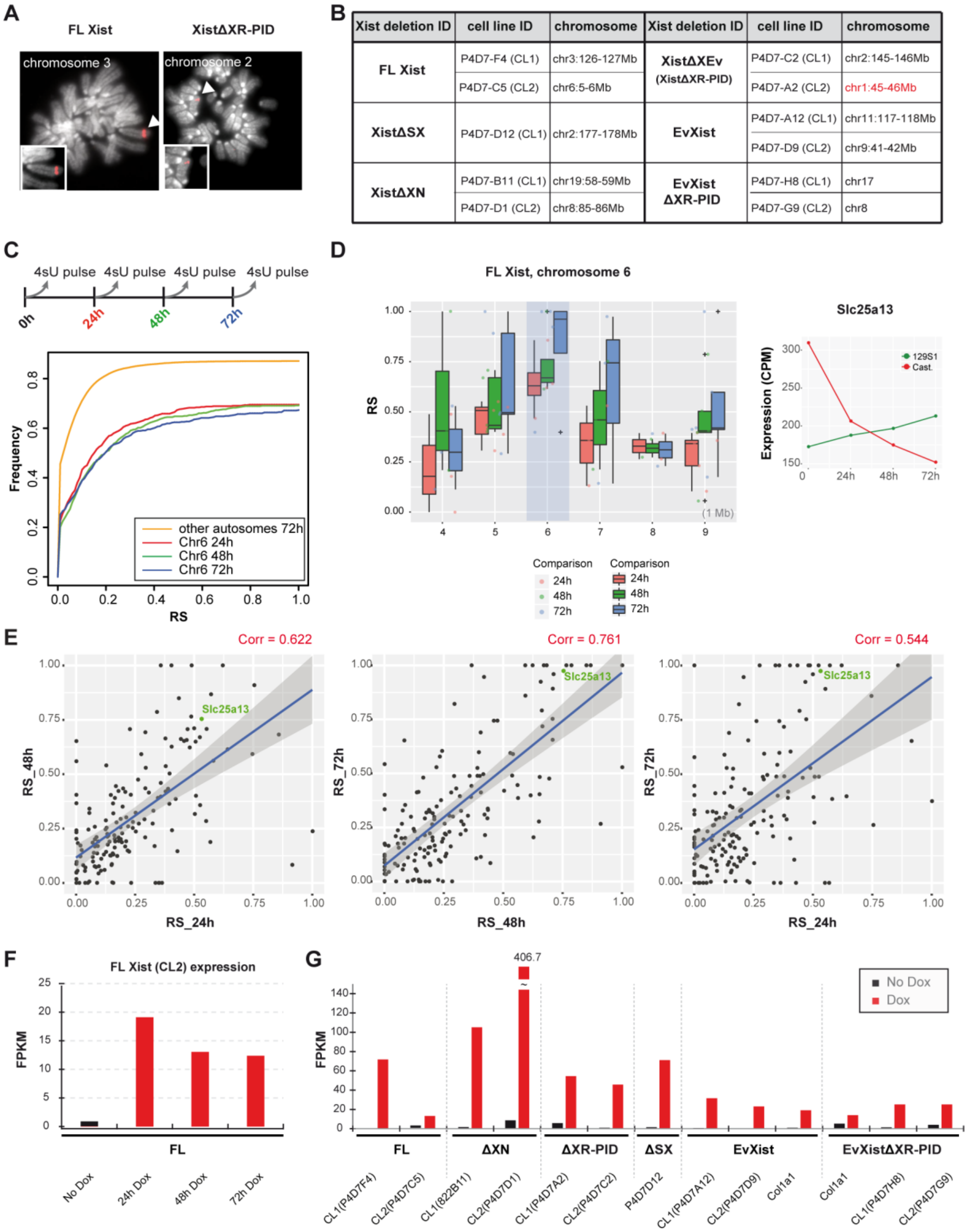

Figure S2. Related to Figure 2. Gene silencing analysis for Xist transgenes

(A) Examples of DNA-FISH analysis mapping Xist transgenes on metaphase spreads. Inset illustrates zoom in of chromosome with Xist transgene. Arrowhead indicates chromosome

with Xist transgene signal (red). DNA was counterstained with DAPI.

(B) Chromosome location of the mapped Xist transgenes in this study, as revealed by RS analysis and confirmed by DNA FISH. All Xist transgenes were integrated in the Cast allele, except for cell line P4D7-A2 where it is in the 129S1 allele (marked in red).

(C) Cumulative distribution function plot showing the RS distribution for genes on chr6 during a time-course experiment. The curves with red, green, blue colours indicate the RS score for 24 h, 48 h, 72 h, respectively. The orange line shows RS on all other autosomes at 72 h, attributable to stochastic effects.

(D) The boxplot (left) shows RS distributions for genes located in the proximal 10 Mb window of chr6, illustrating that within that window, the 6th Mb region shows the maximum silencing. The allelic expression for Slc25a13 gene through the time-course experiment is illustrated (right). The red and green lines are Cast (Inactive) and 129S1 (Active) allele, respectively.

(E) Scatterplot showing correlation of the RS for genes located in the proximal 20 Mb region of chromosome 6 during the time-course experiment. The 99% confidential interval is highlighted in grey. *Slc25a13* gene is located in the maximal silencing region. Pearson's correlation coefficient for each comparison is shown above.

(F) Xist expression levels during a time-course of doxycycline induction are represented as FPKM for each time point.

(G) Xist expression levels are represented as FPKM for each cell line listed in (B) before and after 72 h doxycycline treatment.

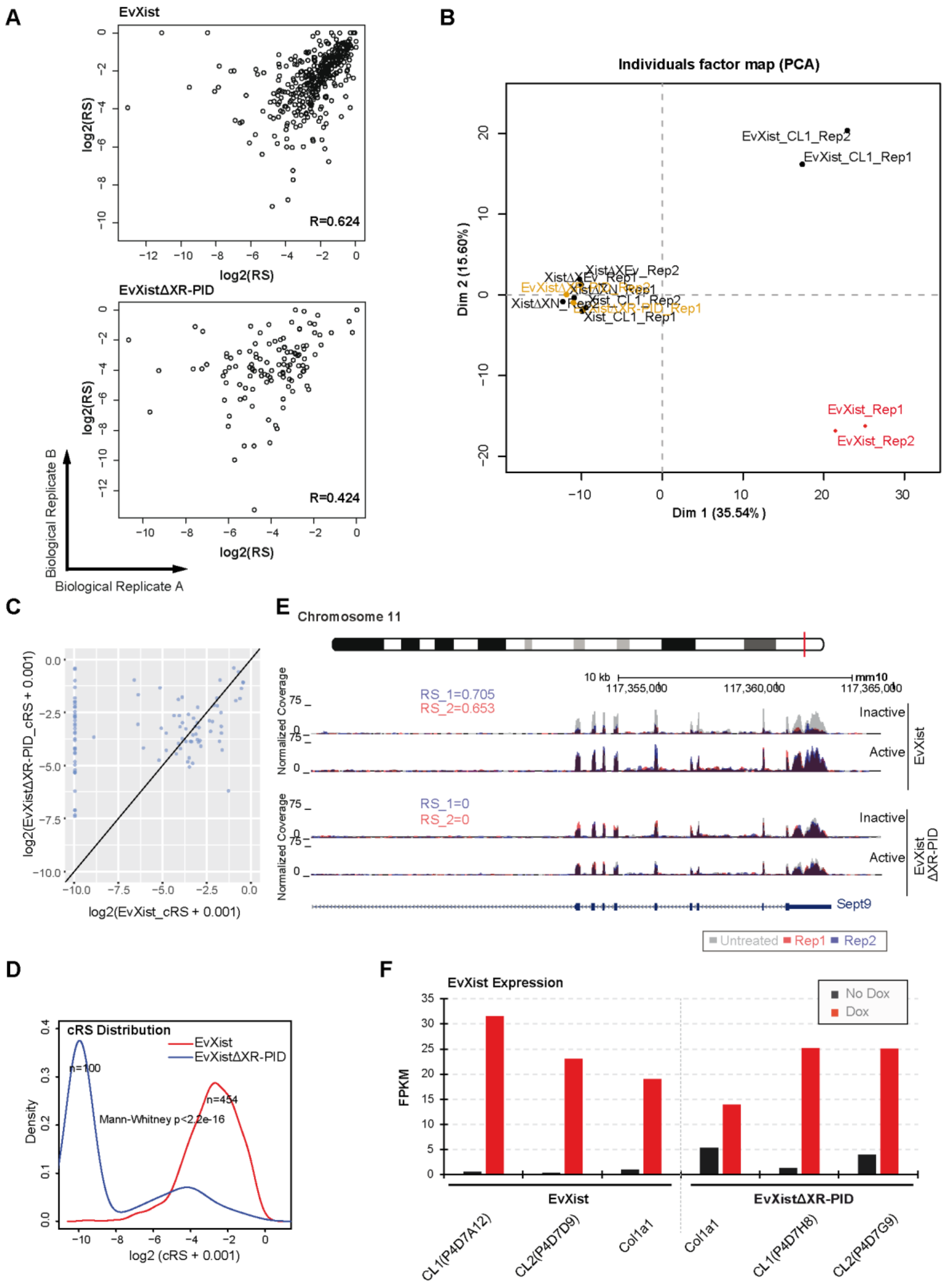

A
EvXist
log2(RS)
R=0.624
EvXistΔXR-PID
R=0.424
Biological Replicate B
Biological Replicate A
B
Individuals factor map (PCA)
EvXist_CL1_Rep2
EvXist_CL1_Rep1
EvXist_Rep1
EvXist_Rep2
Dim 2 (15.60%)
Dim 1 (35.54%)
C
log2(EvXistΔXR-PID_cRS + 0.001)
log2(EvXist_cRS + 0.001)
D
cRS Distribution
EvXist
EvXistΔXR-PID
n=100
n=454
Mann-Whitney p<2.2e-16
Density
log2 (cRS + 0.001)
E
Chromosome 11
10 kb
mm10
117,355,000
117,360,000
117,365,000
RS_1=0.705
RS_2=0.653
RS_1=0
RS_2=0
Normalized Coverage
Inactive
Active
EvXist
EvXist ΔXR-PID
Sept9
Untreated
Rep1
Rep2
F
EvXist Expression
No Dox
Dox
FPKM
EvXist
EvXistΔXR-PID
CL1(P4D7A12)
CL2(P4D7D9)
Col1a1
Col1a1
CL1(P4D7H8)
CL2(P4D7G9)

Figure S3. Related to Figure 3. Chromosome 11 silencing by targeted EvXist and EvXistΔXR-PID transgenes

(A) RS correlation between two biological replicates. Upper: EvXist; Bottom: EvXistΔXR-PID. X and Y axis indicate log2-transformed RS.

(B) PCA Analysis of RS for distal 60 Mb genes on chromosome 11. EvXist and EvXistΔXR-PID cells are indicated by red and yellow dots respectively. Another independent cell line, with an insertion of EvXist (EvXist_CL1) in a different region of chromosome 11 is also shown for comparison.

(C) Scatterplot showing differences of the calibrated (c) RS between EvXist and EvXistΔXR-PID for the 100 genes which are silenced in EvXistΔXR-PID cells. Axis values indicate log2-transformed cRS.

(D) The overall calibrated RS density distribution for genes silenced by EvXist (red curve) or EvXistΔXR-PID (blue curve). The x-axis represents the log2-transformed cRS.

(E) The overlay genome browser tracks for the *Sept9* locus, indicated by red lines in the upper chromosome ideogram, is shown. RS and q-value for EvXist and EvXistΔXR-PID is indicated in the Figure. Q-values are 0 and 0.612 respectively. The grey tracks represent control condition without dox treatment, red and blue are two biological replicates of 72 h dox treatment. The active allele (129S1) and inactive allele (Cast) are indicated (right).

(F) Xist expression levels are represented as FPKM for each cell line before and after 72 h doxycycline treatment. For comparison, Xist expression level is represented for all cell lines harbouring EvXist or EvXistΔXR-PID, either randomly integrated in the genome (P4D7A12, P4D7D9, P4D7H8, P4D7G9), or targeted to the *Col1a1* locus.

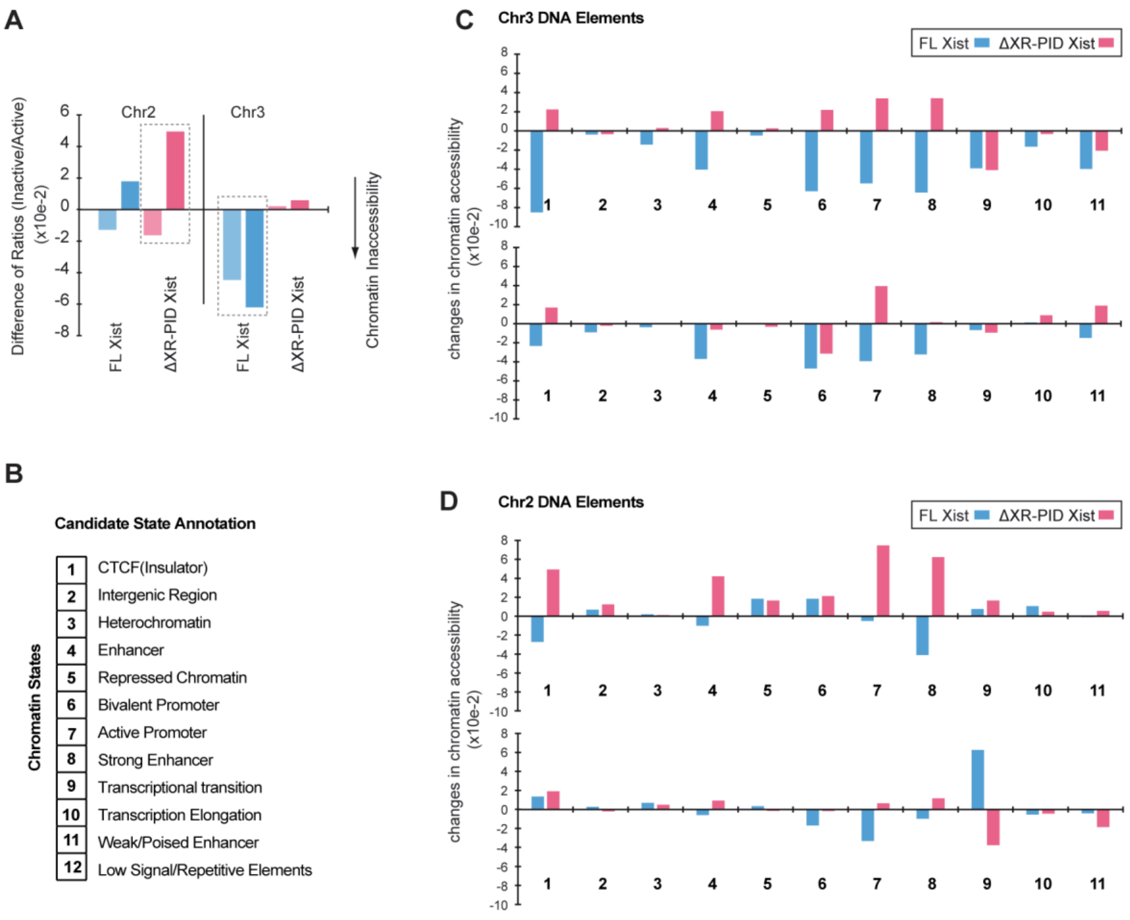

Figure S4. Related to Figure 4. XR-PID is specifically required for reduced chromatin accessibility of open chromatin regions over Xist domains

(A) Whole chromosome differences of allelic ratios (Inactive/Active) between Dox and NoDox cells expressing Xist (chr3) and XistΔXR-PID (chr2). Negative values indicate Xist-induced chromatin inaccessibility.

(B) 12 classes of ChromHMM states of mouse embryonic stem cells were employed to calculate the chromatin accessibility changes in each *cis*-element category upon Xist or XistΔXR-PID induction.

(C and D) Comparison of chromatin accessibility changes in DNA *cis*-elements on chr3 (C) and chr2 (D). The top and bottom part of each panel represents independent biological replicates. Blue and Red indicate FL Xist (integrated in chr3) and ΔXR-PID Xist expressing cells (integrated in chr2).

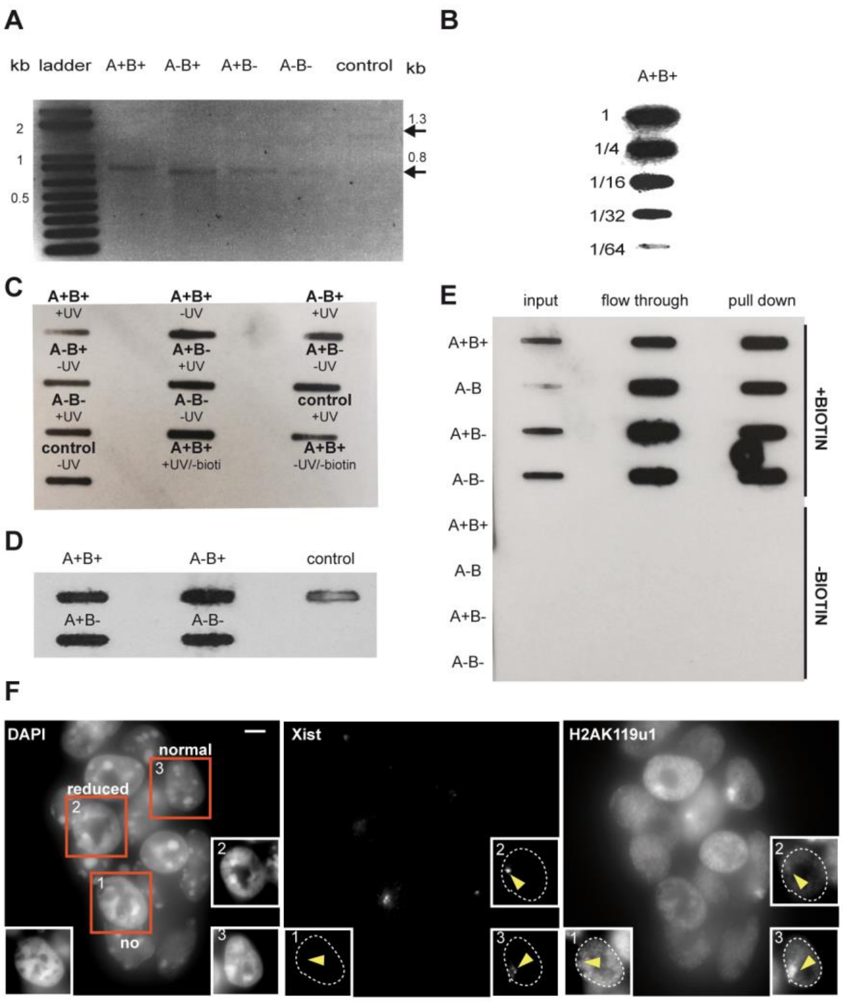

Figure S5. Related to Figure 5. Identification of hnRNPK as candidate B-repeat binding protein

(A) *In vitro* transcription products for each construct (labelled as in Fig.5A), analysed on denaturing gel (0.8% agarose). 1.3 kb FLuc RNA was used as a control.

(B) Titration of dUTP-biotin (starting concentration of 2.5 mM) to determine optimal incorporation for labelling of the same amount of *in vitro* transcribed Xist RNA, here shown for Xist A+B+. A 1/32 concentration was used for subsequent experiments.

(C) Slot blot assay of 1μg of RNA of each construct using biotin antibody. Biotin incorporation and detection was checked in the presence or absence of UV crosslinking. FLuc RNA was used as a control. Xist A+B+ without biotin was used as a negative control.

(D) Slot blot assay of constructs (named as in Fig.5A) using a biotin antibody for detection in the experimental conditions tested in B. 1 μg of RNA was loaded.

(E) Recovery of transcribed RNAs was verified by slot blot. 1 μg of the input, 1/10 of the flow-through, and 1/50 of the pull-down were loaded, determined by analysis of biotin levels. Non-biotinylated constructs were used as negative controls for the assay.

(F) Example of ImmunoFISH analysis of Xist RNA and H2AK119u1 after 24 h siRNA mediated knockdown of hnRNPK siRNA. Wide-field images represent average of 10 stacks. Insets show enlarged single stacks. Arrows point to Xist domains. Each box represents a different category of H2AK119ub1 domains as indicated, and quantified in Figure 5F. Scalebar is 5 μm.

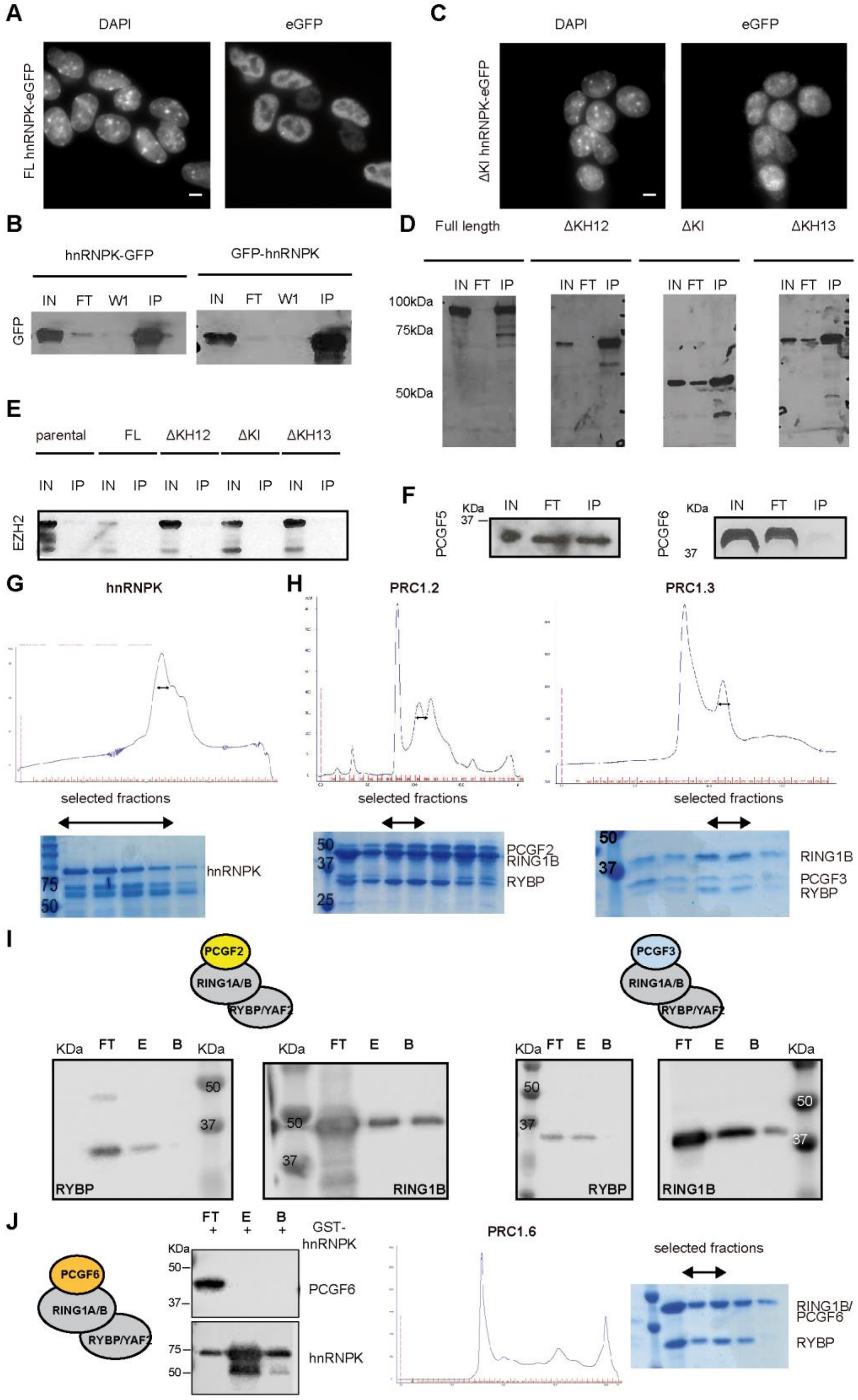
A
DAPI
eGFP
FL hnRNPK-eGFP
C
DAPI
eGFP
ΔKI hnRNPK-eGFP
B
hnRNPK-GFP
GFP-hnRNPK
IN FT W1 IP
IN FT W1 IP
GFP
D
Full length
ΔKH12
ΔKI
ΔKH13
IN FT IP
100kDa
75kDa
50kDa
E
parental
FL
ΔKH12
ΔKI
ΔKH13
IN IP IN IP IN IP IN IP IN IP
EZH2
F
KDa
IN FT IP
PCGF5
PCGF6
37
G
hnRNPK
selected fractions
75
50
H
PRC1.2
PRC1.3
selected fractions
PCGF2
RING1B
RYBP
RING1B
PCGF3
RYBP
50
37
25
I
PCGF2
RING1A/B
RYBP/YAF2
PCGF3
KDa FT E B KDa
RYBP
RING1B
J
FT E B
GST-hnRNPK
PCGF6
hnRNPK
PRC1.6
selected fractions
RING1B/PCGF6
RYBP

Figure S6. Related to Figure 6. hnRNPK interacts with PCGF3/5-PRC1.

(A) Example illustrating detection of hnRNPK-eGFP expression in stable mESC cell line. DNA was counterstained with DAPI. Images represent single stacks. Scale bar is 5 μm.

(B) IP of C- and N-terminus eGFP tagged hnRNPK mESCs using a GFP antibody. 0.1% of the input (IN), 0.1% of the flow-through (FT), 0.1% of the first wash (W1), and 10% of the pull down (IP) were loaded.

(C) As in (A) for ΔKIhnRNPK-eGFP transfected mESC cell line.

(D) IP of hnRNPK and deletion constructs in mESCs using a GFP antibody. 0.1% of the input (IN), 0.1% of the flow-through (FT), and 10% of the pull down (IP) were loaded for each of the named constructs.

(E) Control CoIP showing PRC2 protein EZH2 does not interact with hnRNPK or mutant hnRNPK proteins. Loading was as in (D).

(F) CoIP of PCGF5 by hnRNPK-eGFP. PCGF6 was used as a negative control (see also below). Loading was as in (D).

(G and H) Examples of gel filtration of recombinant hnRNPK-GST (G) and PCGF2-PRC1 and PCGF3-PRC1 (H). A280 traces are shown above with CBB stained gels highlighting proteins of interest below.

(I) Western blots illustrating presence of RING1B and RYBP in the pull-down of recombinant PCGF3-PRC1 but not PCGF2-PRC1, using recombinant GST-hnRNPK. 0.1% of flow through (FT), 20% of eluate (E) and 33% of bead bound (B) were loaded.

(J) Purification of PRC1.6 and western blot showing absence of interaction with hnRNPK-GST as in (G-H) above, and in Figure 6C. Loading of the western blot was as in Figure 6C.

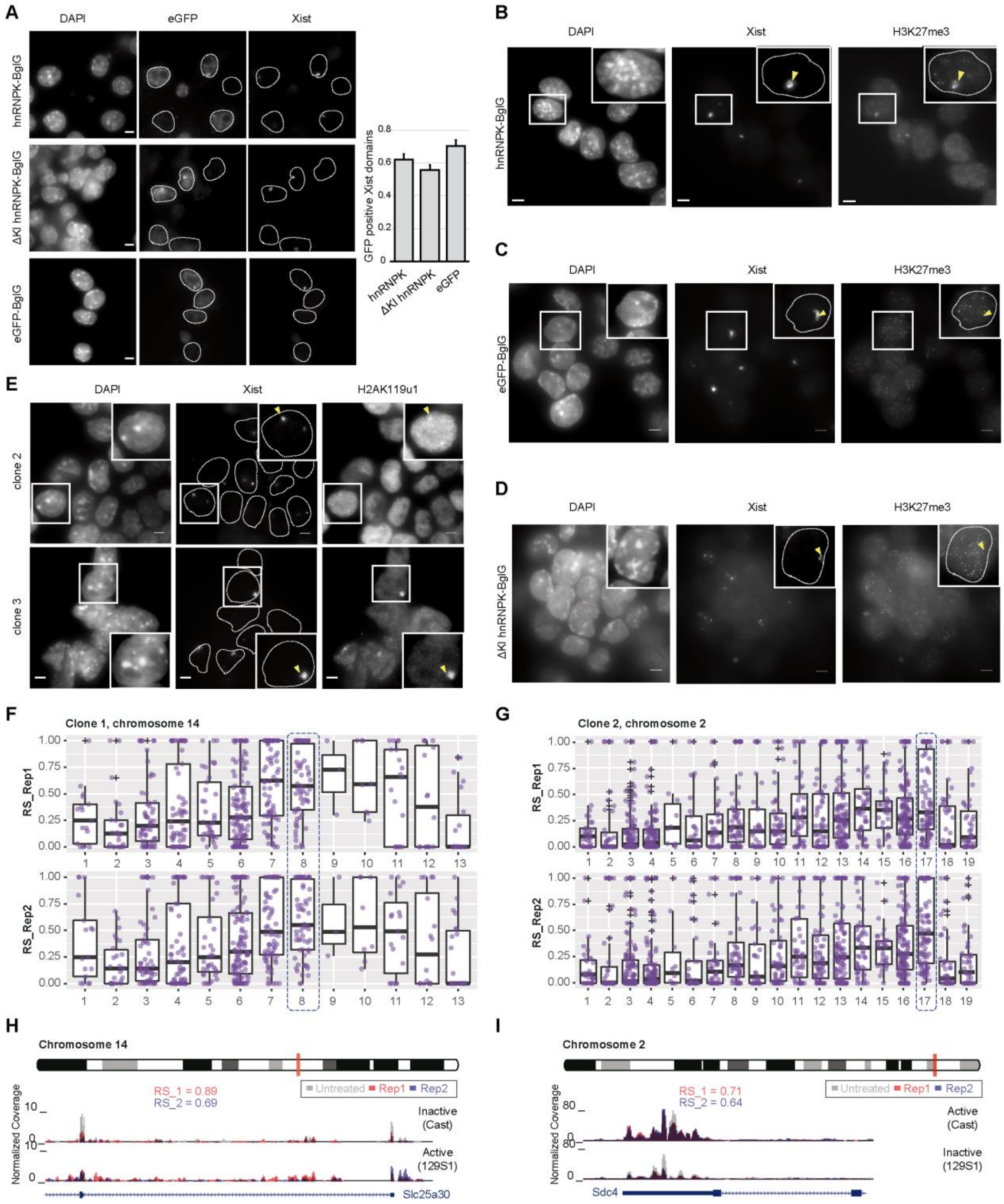

Figure S7. Related to Figure 7. Tethering hnRNPK is sufficient for Xist-dependent Polycomb recruitment

(A) Examples showing ImmunoFISH analysis of Xist RNA and eGFP-BglG fusion proteins 24 h after induction, illustrating that each of the named constructs accumulates over the

inactive chromosome. Wide-field images are average of 6 consecutive z-sections. DNA was counterstained with DAPI. Scale bar is 5 μm. Bar blots represent quantification for individual cell-lines, error bars represent sd across 3 biological replicates (n>100).

(B-D) Examples of ImmunoFISH detection of Xist and H3K27me3 with constructs expressing BglG-hnRNPK-eGFP (B), BglG-eGFP (C), BglG-ΔKIhnRNPK-eGFP (D). Wide-field images represent average of 6 consecutive z-sections. Arrows indicate Xist domains. Individual cells are shown enlarged as individual z-sections. Scale bar is 5 μm.

(E) Examples illustrating H2AK119u1 foci were detected in two independent cell lines (clone 2 and 3), expressing the BglG-hnRNPK-eGFP construct. Wide-field images represent average of 6 consecutive z-sections. Arrows indicate Xist domains. Individual cells are shown enlarged as individual z-sections. Scale bar is 5 μm. DNA was counterstained with DAPI.

(F-G) RS distribution in continuous 10 Mb windows across chromosome 14 (F) and chromosome 2 (right). Purple dots represent the RS for each gene. Blue boxes indicate maximum RS window.

(H-I) UCSC genome browser tracks showing the *Slc25a30* locus (H) and *Sdc4* locus (I) after induction of FL Xist or XistΔXR-PID respectively. Grey tracks represent no-dox control. Red and blue are two biological replicates with 72 h dox treatment. The active and inactive allele are indicated. Chromosomal location of *Slc25a30* and *Sdc4* is indicated with a red bar on the chromosome ideogram above each example.

Supplemental Tables

Table S3. Related to STAR Methods. List of Oligonucleotides.

REAGENT or RESOURCE	SOURCE	IDENTIFIER
**hnRNPK_F, LIC** tacttccaatccATGGAGACCGAACAGCCAGA	This study	LIC cloning
**hnRNPK_R, LIC** tatccacctttactTTAGAATCCTTCAACATCTGCAT	This study	LIC cloning
**hnRNPK_F1, LIC** tacttccaatccATGGAAATTCTGAAGAAAATCAT	This study	LIC cloning
**hnRNPK_F2, LIC** tacttccaatccATGAAACCTGATAGGGTTGTAGA	This study	LIC cloning
**hnRNPK_F3, LIC** tacttccaatccATGGGGTTCAGTGCTGATGAAAC	This study	LIC cloning
**hnRNPK_F4, LIC** tacttccaatccATGGAACCACAGGGTGGTTCTG G	This study	LIC cloning
**hnRNPK_R2, LIC** tacttccaatccATGTTGACCAGGAAGATTAAAC	This study	LIC cloning
**MluIPacI_F, LIC** tacttccaatccGGAGAATTCGAGCTCGGT	This study	LIC cloning
**MluIPacI_R, LIC** tatccacctttactTTATTGCCAGAGTTTAAT	This study	LIC cloning
**BglG_F, Gibson assembly** tcatcattttggcaaagaattccATGAACATGCAAATCA CCAAA	This study	Gibson assembly
**BglG_R, Gibson assembly** ggattggaagtacaggttctccACGGTGGCGACCGGT AGCAA	This study	Gibson assembly
**ColA1_CrisprF_T1 , gRNA** caccgTAGAAGGGGCCTTCTAATGC	This study	CRISPR HR
**ColA1_CrisprF_T2, gRNA** caccGAGGTTCATGAGCCCTCAAA	This study	CRISPR HR
**ColA1_CrisprF_T3, gRNA** caccGCCCCTTCTATACTAAATTA	This study	CRISPR HR
**MEDS-A, Tn5 assembly** Alexa594- GTCTCGTGGGCTCGGAGATGTGTATAAGAG ACAG	This study	Tn5 assembly
**MEDS-B, Tn5 assembly** Alexa594- TCGTCGGCAGCGTCAGATGTGTATAAGAGA CAG	This study	Tn5 assembly
**MEDS-REV, Tn5 assembly** Phos-CTGTCTCTTATACACATCT	Chen et al., 2016	Tn5 assembly

Table S4. Related to STAR methods. List of Antibodies.

REAGENT or RESOURCE	SOURCE	IDENTIFIER
H2AK119u1, Rabbit, IF	Cell Signaling	cat#8240
H3K27me3, Mouse, IF	Active Motif	cat#61017

GFP, Chicken, IP, IF	Abcam	cat#ab13970
GFP, Rabbit, IF	Abcam	cat# Ab290
RYBP, Rabbit, IF	Millipore	cat# AB3637
RING1B, Mouse, WB, IF	Gift from H. Koseki	N/A
RYBP, Rabbit, WB	Millipore	cat# AB3637
EZH2, Rabbit, WB, IF	Cell Signaling	cat# 5246
EED, Mouse, WB, IF	Gift from A. Otte	N/A
Histone H3, Rabbit, WB	Abcam	cat# ab1791
hnRNPK, Rabbit, WB, IP	NEB	cat# 4675
Neutravidin-HRP, Rabbit, WB	Life Technologies	cat# 31030
Anti-mouse Ig, HPR, Donkey, WB	Amersham	cat# NA934V
Anti-mouse Ig, HPR, Donkey, WB	Amersham	cat# NA931V
Alexa 568 anti-mouse IgG, Goat, IF	Life Technologies	cat# A11031
Alexa 488 anti-mouse IgG , IF	Life Technologies	cat# A11029
Alexa 568 anti-rabbit IgG, Goat, IF	Life Technologies	cat# A11034
Alexa 488 anti-rabbit IgG, Goat, IF	Life Technologies	cat# A11008
Alexa 488 anti-chicken IgG, Goat , IF	Life Technologies	cat# A11039
